# Digging into the 3D Structure Predictions of AlphaFold2 with Low Confidence: Disorder and Beyond

**DOI:** 10.3390/biom12101467

**Published:** 2022-10-13

**Authors:** Apolline Bruley, Jean-Paul Mornon, Elodie Duprat, Isabelle Callebaut

**Affiliations:** Sorbonne Université, Muséum National d’Histoire Naturelle, UMR CNRS 7590, Institut de Minéralogie, de Physique des Matériaux et de Cosmochimie, IMPMC, 75005 Paris, France

**Keywords:** long foldable segments, pyHCA, soluble domains, protein sequence, conditional order, hidden order, dark proteomes, intrinsically disordered domains

## Abstract

AlphaFold2 (AF2) has created a breakthrough in biology by providing three-dimensional structure models for whole-proteome sequences, with unprecedented levels of accuracy. In addition, the AF2 pLDDT score, related to the model confidence, has been shown to provide a good measure of residue-wise disorder. Here, we combined AF2 predictions with pyHCA, a tool we previously developed to identify foldable segments and estimate their order/disorder ratio, from a single protein sequence. We focused our analysis on the AF2 predictions available for 21 reference proteomes (AFDB v1), in particular on their long foldable segments (>30 amino acids) that exhibit characteristics of soluble domains, as estimated by pyHCA. Among these segments, we provided a global analysis of those with very low pLDDT values along their entire length and compared their characteristics to those of segments with very high pLDDT values. We highlighted cases containing conditional order, as well as cases that could form well-folded structures but escape the AF2 prediction due to a shallow multiple sequence alignment and/or undocumented structure or fold. AF2 and pyHCA can therefore be advantageously combined to unravel cryptic structural features in whole proteomes and to refine predictions for different flavors of disorder.

## 1. Introduction

AlphaFold2 [1] and RoseTTAfold [2] have recently achieved an impressive breakthrough in the field of structural biology, providing accurate models of three-dimensional (3D) structures of proteins based on only knowledge of their amino acid sequences alone. Based on deep-learning techniques, they take advantage of the vast existing knowledge of protein sequences and 3D structures, recently expanded through environmental genomics and structural genomics approaches. In particular, they extensively used evolutionary information to detect co-variation of residues (or correlated mutations), the underlying idea being that residues that have co-evolved are close in 3D space. The first version of the AlphaFold2 database (AFDB v1) [3] included predictions for a very large part of proteomes from 21 widely studied organisms. It has been extended to provide open access to over 200 million predictions, covering nearly every organism with protein sequence data. This provides the scientific community with a wealth of knowledge, which could accelerate the understanding of protein structure-function relationships and have a profound impact on many areas of biology, including human health and the environment.

Several studies have already been conducted to estimate the extent to which AlphaFold2 (AF2) improves the coverage in structural biology, as well as to analyze its current advantages and limitations (e.g., [4,5,6,7,8,9,10]). One striking feature of AF2 is that it provides a per-residue metric, reflecting confidence in the structural assignment (predicted local distance difference test (pLDDT)) [1]. High values of pLDDT are observed for folded domains, contrasting with low values typically associated with linkers and unstructured or disordered regions [11]. The relevance of pLDDT as a predictor of disorder has been supported on the CAID benchmark dataset [12] and compared to other state-of-the-art disorder predictors, such as SPOT-Disorder2 or IUPred2 [1,4,11].

At least two questions need to be considered when focusing on very low confidence regions (pLDDT < 50) in AF2 predictions, which are assumed to be globally disordered. The first question is whether it is possible to reveal conditional order within these intrinsically disordered regions (IDRs), from amino acid sequence information alone. Such IDRs may be involved in molecular recognition, to which hydrophobic interactions make major contributions [13,14]. In many cases, these regions undergo a disorder-to-order transition (induced folding) to a more structured state upon binding with a partner [15]. High-resolution multi-dimensional NMR studies have demonstrated that such IDRs, ranging in length from 10 up to 70 amino acids and referred to over time by different names ([16,17], molecular recognition elements [18], primary contact sites [19], preformed structural elements [20], pre-structured motifs [21]), can be pre-populated by transient local structural elements, presaging the target-bound conformation [21]. The plasticity of these IDRs can allow for a range of secondary structures in the bound state, as shown by the example of the p53 tumor suppressor protein [22]. Some IDRs are also able to retain a significant degree of structural heterogeneity in the bound states [23], leading to the definition of fuzzy complexes [24,25]. Some IDRs involved in molecular recognition consist of or incorporate short linear motifs (SLiMs), i.e., short conserved sequences, which enable low affinity, transient, and conditional interactions and are often located within disordered regions [26]. Specifying the structural unit in which these short interacting motifs are embedded should inform on the global features of the interaction (such as affinity, specificity, fuzziness). Regarding conditional order, another question to consider is whether it is possible to identify, within these very low confidence AF2 predictions, longer binding IDRs that meet the definition of intrinsically disordered domains (IDD) [27,28,29], which must be stabilized by a partner within protein complexes to adopt a stable fold?

The second issue related to very low pLDDT regions is to evaluate whether some might not be disordered and might still adopt a well-folded 3D structure, but AF2 cannot predict it (what we call “hidden order”). This hypothesis is conceivable as the co-evolutionary information, necessary to predict inter-residue contacts, is lacking for some protein sequences. These proteins, not predicted as disordered, escape any annotation coming from sequence or structure databases and constitute the dark proteome [30,31]. They still represent 10% of the human proteome after annotation with the AlphaFold2 predictions [7]. 

We recently analyzed the pLDDT values observed for the AF2 3D structure predictions on the 21 reference proteomes (AFDB v1) in light of another metric, called the HCA score (Bruley et al. [32]). The HCA score is based on Hydrophobic Cluster Analysis (HCA), a two-dimensional approach allowing the analysis of the content of an amino acid sequence in regular secondary structures (see [33] for a recent review of the methodology). Indeed, the hydrophobic clusters defined by this approach mainly correspond to the positions of regular secondary structures constituting the building blocks of folded domains [34,35,36]. The analysis of the composition of a sequence in hydrophobic clusters thus provides information on its architecture in domains and the disorder/order content of the delineated domains. A tool has been developed to automatically partition protein sequences into foldable segments based on a measure of hydrophobic cluster density [37]. The calculation of an HCA score provides information about the composition of the sequence in clusters and hydrophobic amino acids within the clusters, which thus reflects the overall order/disorder ratio of the foldable segments [32].Using this HCA score, we disentangled different types of disorder and appreciate disorder-to-order continuum. While residues with low-pLDDT values were enriched in non-foldable segments, a significant portion of foldable segments with HCA scores typical of well-folded domains also had low mean pLDDT values in AF2 3D structure predictions. This suggests that these regions carry specific functional information (corresponding to the two cases mentioned above) that remains unraveled by AF2 (Bruley et al. [32]).

Here, we further explored the source of this apparent inconsistency between foldability and low confidence AF2 prediction, which is widely assimilated to disorder in the literature. To this end, we analyzed, from the same 21 reference proteomes, the long soluble-like foldable segments as defined by the pyHCA tool, whose residues all have a very low AF2 pLDDT value (hereafter referred to as full-VL segments). Particular focus was on segments of length > 30 amino acids, which corresponds to the minimum length considered for globular domains [38]. Moreover, this minimal length excludes a large number of short motifs (SLiMs) undergoing induced folding and which are otherwise associated with higher HCA score values (Bruley et al. [32]). To analyze these long, soluble-like full-VL segments, we considered four features related to their amino acid sequences and 3D structures, as predicted by AF2. We described these 3D structure models by the proportion of residues involved in a regular secondary structure (RSS) and by the proportion of residues accessible to the solvent. In addition, we described the protein sequences on which these predictions were based, by the proportion of residues predicted as disordered by IUPred2 and by the average number of homologs per residue as found in the large environmental BFD database. The latter feature allowed us to consider co-evolution information, essential for the reliable prediction of amino acid contacts by AF2. We compared these features to their distribution for long soluble-like foldable segments whose residues all have a very high pLDDT values (hereafter referred to as full-VH segments), for the 21 proteomes included in AFDB v1. 

## 2. Material and Methods

### 2.1. Proteomes from AlphaFold Protein Structure Database v1

Amino acid (aa) sequences and predicted 3D structures were downloaded from the AlphaFold Protein Structure database (AFDB) v1 ([3], https://alphafold.ebi.ac.uk, accessed on 21 July 2021) for the 21 reference model-organism proteomes. The per-residue model confidence values (pLDDT) were extracted from the 3D coordinate files (B-factor column in PDB format). 

### 2.2. Delineation of Soluble-Like Foldable Segments within Protein Sequences

The *segment* function of the pyHCA tool (provided at https://github.com/DarkVador-HCA/pyHCA, accessed on 14 September 2022) was used to automatically delineate foldable segments (FS), i.e., segments with a high density of hydrophobic clusters (HC), as defined by the Hydrophobic Cluster Analysis (HCA) [33]. HC consist of strong hydrophobic amino acids (V,I,L,M,F,Y,W) and are separated from each other by at least four other amino acids or a proline. For FS delineation, cysteine (C) is integrated into the hydrophobic alphabet and HC consist of only one or two consecutive hydrophobic amino acid(s) are not considered, as they are mainly associated with coils [36].

The HCA score, which measures the density of hydrophobic clusters and strong hydrophobic amino acids of foldable segments (Bruley et al. [32]), was calculated using the *segment* function (pyHCA tool). Soluble-like segments were defined according to an HCA score value between −1 and 3.5.

### 2.3. Description of Sequence and Structural Features

Our final dataset consisted of proteins from AFDB v1, encompassing at least one long (>30 a.a.), globular soluble-like (−1 ≤ HCA score ≤ 3.5) foldable segments, entirely made of residues with very low (VL) or very high (VH) 3D prediction confidence (pLDDT ≤ 50 and pLDDT > 90, respectively). We considered four different features to characterize the amino acid sequence and AF2 3D models of these segments, as detailed below. For each feature, we defined a threshold value based on the distribution of these full-VH segments and delimiting an interval encompassing at least 95% of them. These threshold values were further used for the dataset description by binary trees. 

#### 2.3.1. Per-Residue Disorder Prediction 

Disorder was predicted using the IUPred2A [39] *long* disorder predictor on the whole protein sequences. IUPred2A calculates a per-residue score between 0 and 1 that reflects the estimated stabilizing effect of other residues on each residue of one amino acid sequence. The coverage of the FS by disorder was then calculated (in percentage of the segment length), considered as disordered amino acids having a score above 0.5. The coverage threshold was set to 33.4%. The number of FS with a value below this threshold were as follows: 14,077 segments over 30,644 and 10,827 segments over 11,395 in case of full-VL and full-VH, respectively.

#### 2.3.2. Known Homologs

The multiple sequence alignments used to build the AlphaFold2 models were not provided in AFDB repositories. Therefore, a search for known homologs in the reduced Big Fantastic Database (BFD) was performed using *jackhmmer* (from HMMER 3.3.2 [40], http://hmmer.org/, accessed on 14 September 2022). The parameters (e-value threshold of 0.0001, 1 iteration) were those used by AF2 in the similarity search step. The Big Fantastic Database (BFD) [1] (https://bfd.mmseqs.com, accessed on 24 May 2022) is a database containing 2.5 billion clustered protein sequences. It is the most comprehensive database used by AF2 in order to build multiple sequence alignments, gathering sequences from genomic and metagenomic databases (UniprotKB [41] and metaclust [42] and datasets assembled with Plass [43]). The reduced version of BFD contains only representative sequences of each cluster (65,984,053 sequences). This one was downloaded following the recommendations given on the AF2 github (https://github.com/deepmind/alphafold, accessed on 24 May 2022). In this work, the sequence similarity search was performed on the whole protein sequences. The number of aligned sequences per FS position was then calculated and averaged over the length of the FS. The mean number threshold was set to 23.5 BFD homologs per segment residue. The number of FS with a value above this threshold were as follows: 3347 segments over 30,644 and 10,829 segments over 11,395 in case of full-VL and full-VH, respectively.

#### 2.3.3. Secondary Structure Assignment

Secondary structures were assigned from the coordinates of the AF2 3D structure models (PDB files, full-length proteins) using the DSSP program [44] available in the biopython module v1.78 for python v3.6.3. All amino acids found in alpha helices (encoded as “H” in DSSP), 3–10 helices (“G”), Pi helices (“I”), strands (“E”), and isolated beta-bridge residues (“B”) were considered to participate in regular secondary structures (RSS). The percentage of the FS residues participating in a RSS was then calculated. The number of FS with at least 1 RSS were as follows: 10,993 segments out of 30,644 and 11,393 segments out of 11,395 in case of full-VL and full-VH, respectively. 

#### 2.3.4. Solvent Accessibility

Using the same module, the residues relative accessible surface area was calculated. This value was obtained by normalizing the residue accessible surface area (ASA) by the maximum ASA for the residue, computed on Gly-X-Gly tripeptides (where X is the residue of interest). By default, DSSP referred to the Sander and Rost scale for maximum ASA values per residue [45]. We considered a residue to be solvent accessible if the relative ASA was above 0.36 (based on Rost and Sander [45]). The percentage of accessible residues was calculated on each FS. The feature threshold was set to 82.9%. The number of FS with a value below this threshold were as follows: 1908 segments over 30,644 and 10,823 segments over 11,395 in case of full-VL and full-VH, respectively. 

#### 2.3.5. 3D Structure Comparison

The Dali server ([46], http://ekhidna2.biocenter.helsinki.fi/dali, accessed on 14 September 2022) was used to compare the AF2 3D structure models of the foldable segments with PDB experimental 3D structures.

#### 2.3.6. Figure Creation

3D structures were visualized with the UCSF Chimera software [47]. HCA plots were drawn using the DrawHCA program (http://osbornite.impmc.upmc.fr/hca/hca-seq.html, accessed on 14 September 2022). Hydrophobic clusters (HC) affinities for RSS were extracted from HCDB [36]. Binary tree diagrams were created using the R package *ggparty* (https://github.com/martin-borkovec/ggparty, accessed on 14 September 2022).

## 3. Results

### 3.1. General Features of Full-VL and Full-VH Segments from AFDB v1

**Figure 1:**
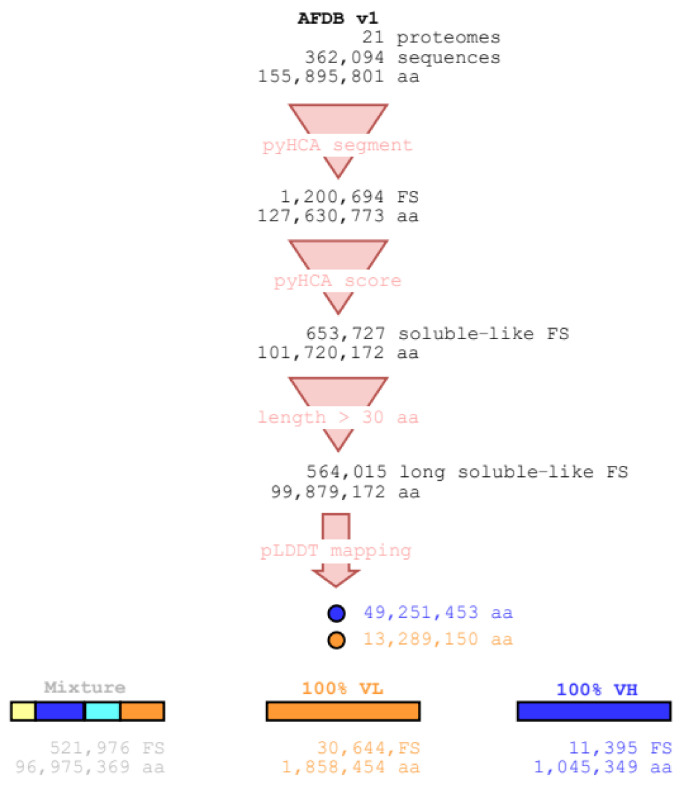
The technical flow for definition of the long soluble-like full-VH and full-VL foldable segments from AFDB v1 by using the pyHCA tool. The number of foldable segments (FS) and the number of residues (aa) are indicated at each step of the flow. The dataset further analyzed in this study consists of the full-VL and full-VH segments. For quantitative details about each of the 21 proteomes, see Supplementary Table S1.

Figure 1 illustrates the technical flow used in this study to extract 30,644 full-VL and 11,395 full-VH long soluble-like foldable segments from AFDB v1 using the pyHCA tool.

Details for each of the 21 proteomes are given in Supplementary Table S1. Most of the residues in AFDB v1 (64.1%) are included in long soluble-like foldable segments (from 55.5% up to 73.9% in the proteomes of *Leishmania infantum* and *E*. *coli*, respectively). These segments are mainly composed of residues with a very high pLDDT value (49.3% VH, 13.3% VL). This trend is also observed for each proteome, except for *Plasmodium falciparum* (23.8% VH, 40.4% VL). However, the set of the full-VL segments is larger than the set of full-VH segments, both in the number of segments and in the number of residues (Figure 1). This trend is observed for each of the 17 eukaryotic proteomes, where at least 9.1% VL residues included in a long soluble-like foldable segment are part of a full-VL segment (up to 22.4% and 24.0% for *Leishmania infantum* and *Plasmodium falciparum*, respectively). On the contrary, less than 6.3% of the VL residues included in a long soluble-like foldable segment are part of a full-VL segment for prokaryotic proteomes, where only a few cases of full-VL segments were found (1, 8, 14, and 29 segments for the archaeon *Methanocaldococcus jannaschii* and the bacteria *Escherichia coli*, *Staphylococcus aureus,* and *Mycobacterium tuberculosis* respectively). Furthermore, for eukaryotic proteomes, less than 2.6% of the VH residues included in a long soluble-like foldable segment are part of a full-VH segment (from 3.7% up to 8.1% for the prokaryotic proteomes). In AFDB v1, the mean length of full-VL segments (60.7 amino acids) is smaller than the mean length of full-VH segments (91.7 aa). This trend is observed for each of the 21 proteomes.

**Figure 2:**
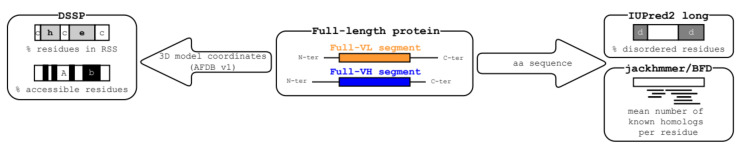
The technical flow for feature description of the segment dataset. Each AFDB v1 full-length protein comprising at least one full-VL or one full-VH long soluble-like foldable segment was analyzed by different tools (DSSP on the 3D coordinates, IUPred2 *long*, and *jackhmmer* on the amino acid sequence) allowing for calculation of four quantitative features describing each segment. Labels used for the different tools are: (i) for DSSP secondary structure assignment: h, helix; e, strand (extended); c, coil; (ii) for DSSP solvent accessibility: A, accessible, b, buried; (iii) for IUPred2 long: d, disorder.

Figure 2 illustrates the technical flow used in this study for the description of the AF2 3D models and protein sequences for the full-VL and full-VH segment datasets. We described each segment by four quantitative features and explored their distribution for each dataset.

### 3.2. Full-VH Segments

**Figure 3:**
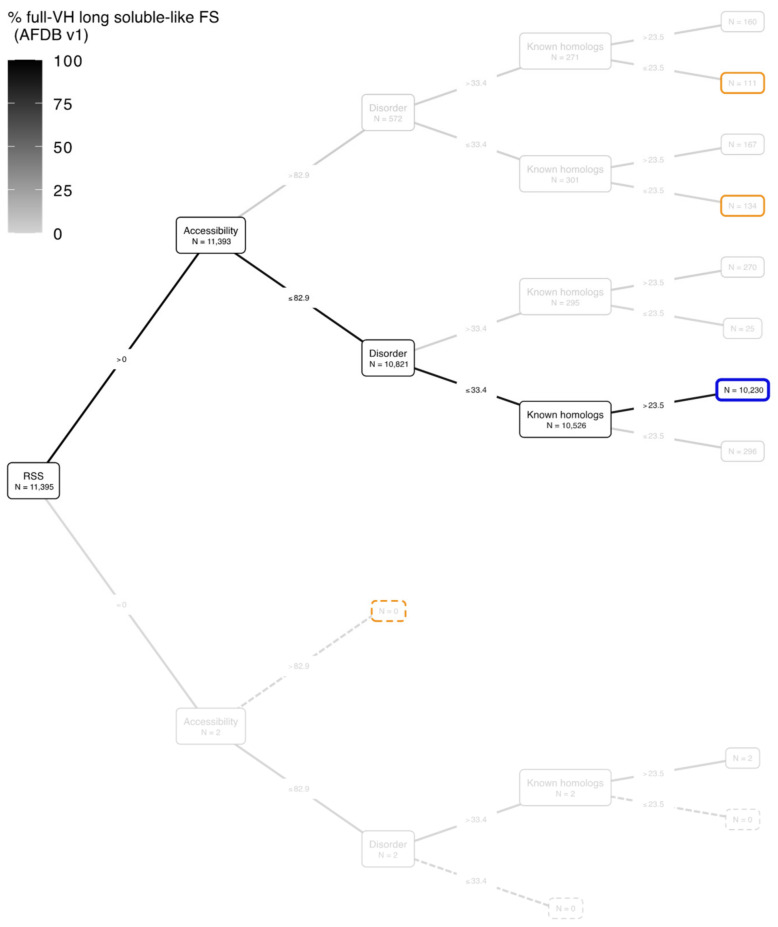
Binary tree diagram of the full-VH segments according to the feature thresholds. The four levels of the tree (from the root on the left to the last internal nodes on the right) correspond to the four features describing the segments (see Figure 2 for the technical flow), as follows: percentage of segment residues participating in a regular secondary structure (RSS), percentage of segment residues accessible to the solvent (Accessibility), percentage of segment residues predicted to be disordered (Disorder), the mean number of BFD homologs per segment residue (Known homologs). The binary conditions based on each feature threshold are indicated on the edges of the tree (for details, see Section 2). The number of foldable segments with a given feature below or above each threshold is indicated in the internal and terminal nodes. The total number of full-VH segments is indicated within the root node. The terminal nodes corresponding to the most abundant subsets of full-VH segments (this figure) and full-VL segments (Section 3.3) are highlighted in blue and orange, respectively. For quantitative details about each of the 21 proteomes, see Supplementary Figure S1.

**Figure 4:**
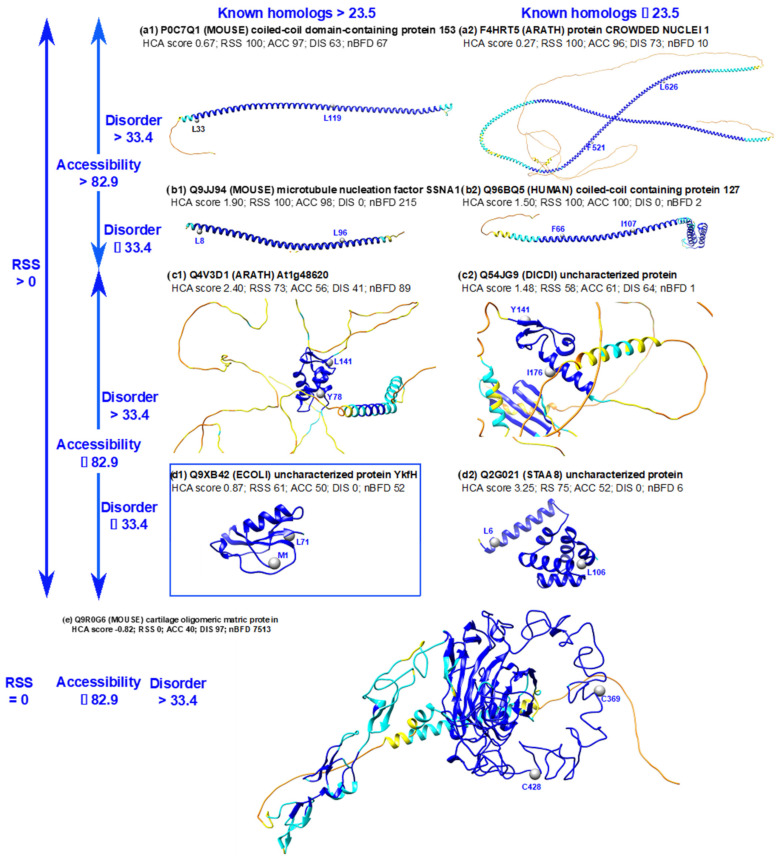
Examples of full-VH soluble-like foldable segments, distinguished according to the four features. The examples were extracted from the binary tree diagram shown in Figure 3. The AF2 3D structure models are colored according to pLDDT values, with the positions of the first and last amino acids of the full-VH soluble-like foldable segments indicated. The corresponding HCA score values are also reported, as well as those of the four features. The example extracted from the most populated leaf in Figure 3 is boxed in blue. HCA plots of the corresponding sequences are illustrated in Supplementary Figure S2. Subfigures a show examples with RSS > 0, Accessibility > 82.9, Disorder > 33.4 and BFD homologs per position > 23.5 (**a1**) and ≤ 23.5 (**a2**). Subfigures b show examples with RSS > 0, Accessibility > 82.9, Disorder ≤ 33.4 and BFD homologs per position > 23.5 (**b1**) and ≤ 23.5 (**b2**). Subfigures c show examples with RSS > 0, Accessibility ≤ 82.9, Disorder > 33.4 and BFD homologs per position > 23.5 (**c1**) and ≤ 23.5 (**c2**). Subfigures d show examples with RSS > 0, Accessibility ≤ 82.9, Disorder ≤ 33.4 and BFD homologs per position > 23.5 (**d1**) and ≤ 23.5 (**d2**). Subfigure (**e**) shows one of the two similar cases with RSS = 0, Accessibility ≤ 82.9, Disorder > 33.4.

Figure 3 depicts the classification of the 11,395 full-VH segments using a binary tree based on the features used to describe the 3D models and the amino acid sequences (see Figure 2 and Section 2 for details). Representative examples of the different categories are shown in Figure 4.

Quantitative thresholds were defined for each feature based on 95% full-VH, except for the proportion of segment residues participating in a RSS, as assigned by DSSP from the AF2 3D models (see Section 2 for details). For this 3D feature, we considered two classes of segments based on the presence/absence of RSS. All the long soluble-like foldable segments whose residues all have a very high pLDDT value (full-VH segments) are associated with the presence of RSS, except for two cases, corresponding to thrombospondin (TSP) repeats (Figure 4e). As observed in the experimental 3D structures that can serve as templates for homology modeling (pdb entries 1yo8 and 3fby), TSP repeats are folded domains with calcium ions bound into the core through acidic (aspartate) residues. The foldable segments delineated here contain conserved cysteine residues that form interdomain disulfide-bridges, providing tight interactions in the wire architecture typical of the TSP-2 signature domain [48].

The most abundant category of full-VH long soluble-like foldable segments (10,230 full-VH segments over 11,395, boxed in blue in Figure 3 and Figure 4(d1)) corresponds to folded domains with low predicted disorder and a high number of BFD homologs. Domains were considered as folded as they contain RSS assembled together and have relative low solvent accessibility due to the involvement of a large number of amino acids in a hydrophobic core. Supplementary Figure S2 provides details of the HCA plots of the foldable segments whose 3D structures are shown in Figure 4. The folded domains contain ~1/3 strong hydrophobic amino acids distributed in clusters, which correspond to the positions of RSS. A significant number of cases also exist with a smaller number of BFD homologs (296 segments, Figure 3 and Figure 4(d2)). Here, the consideration of experimental 3D structures as templates can explain the accurate AF2 prediction (pdb:1sed for the example shown in Figure 4(d2)). Other interesting cases are those of folded domains corresponding to sequences predicted to be disordered for a large part, but which are clearly not (Figure 4(c1,c2) corresponding to histone fold, 29% identity with pdb 2lso-A, and to a case with no obvious similarity with known 3D structures, respectively). Finally, the cases of accurate AF2 predictions associated with models globally accessible to the solvent concern long helices, typical of coiled-coil assembly, whose sequences are predicted as disordered or not (Figure 4(a1,a2,b1,b2)). When no experimental 3D structure is available, the AF2 prediction is supported by a sufficiently informative periodic pattern and self-organizing structure, regardless of the number of BFD homologs.

### 3.3. Full-VL Segments

**Figure 5:**
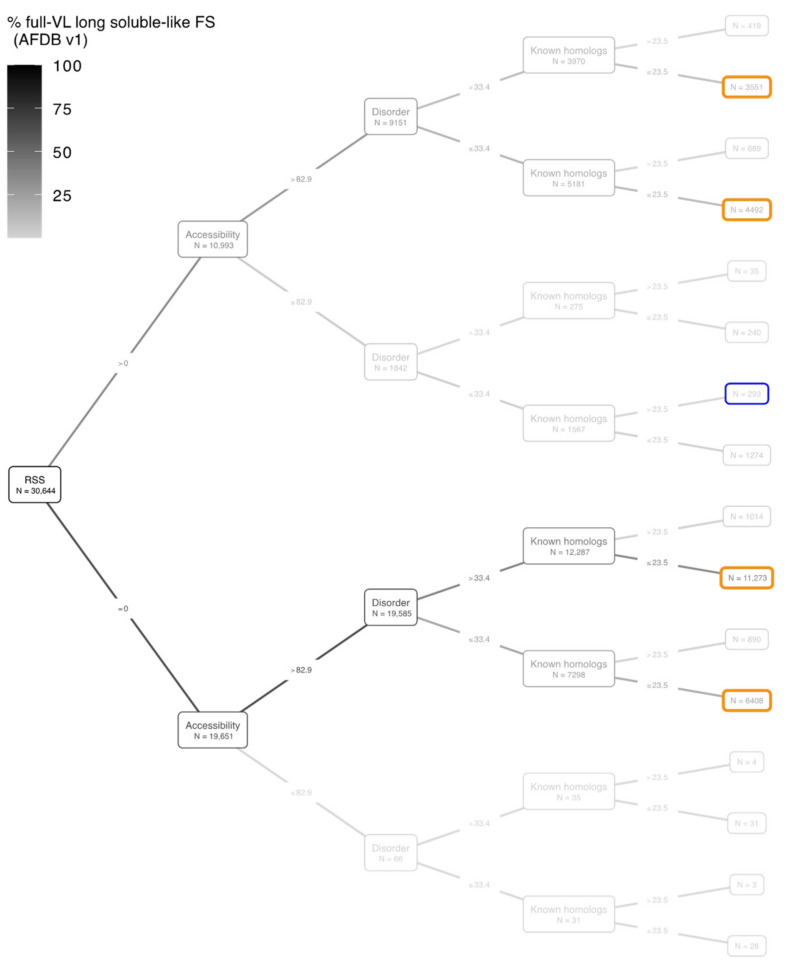
Binary tree diagram of the full-VL segments according to the feature thresholds. The four levels of the tree (from the root on the left to the last internal nodes on the right) correspond to the four features describing the segments (see Figure 2 for technical flow), as follows: percentage of segment residues participating in a regular secondary structure (RSS), percentage of segment residues accessible to the solvent (Accessibility), percentage of segment residues predicted to be disordered (Disorder), mean number of BFD homologs per segment residue (Known homologs). The binary conditions based on each feature threshold are indicated on the edges of the tree (for details, see Section 2). The number of foldable segments with a given feature below or above each threshold is indicated within the internal and terminal nodes. The total number of full-VL segments is indicated in the root node. The terminal nodes corresponding to the most abundant subsets of full-VL segments (this figure) and full-VH segments (Figure 3) are highlighted in orange and blue, respectively. For quantitative details about each of the 21 proteomes, see Supplementary Figure S3.

**Figure 6:**
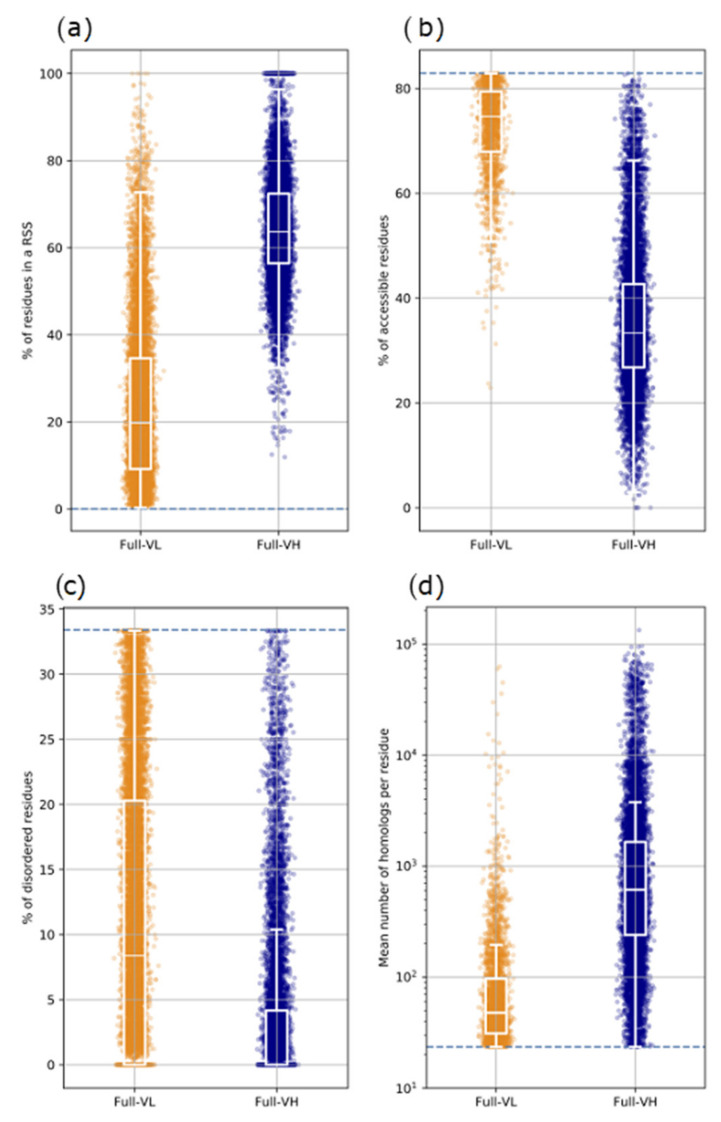
Distribution of features of 3D models and amino acid sequences for full-VH (blue) and full-VL (orange) long soluble-like foldable segments from AFDB v1 (21 proteomes). For the structural feature corresponding to the percentage of segment residues participating in a regular secondary structure (RSS) (**a**), only segments with at least 1 RSS as assigned by DSSP from the full-length protein 3D coordinates are shown (see Section 2 for quantitative details). For each feature in (**b**–**d**), the blue dashed line indicates the threshold value defined based on 95% of the full-VH segments (see Section 2 for details). For both full-VL and full-VH segments, only values falling in these intervals are shown.

Figure 5 shows the binary tree diagram of full-VL, long soluble-like foldable segments, according to the same threshold values as in Figure 3 for full-VH segments. The full-VL segments are much more dispersed across the different categories than the full-VH segments (see boxes in Figure 3 and Figure 5). Four categories are populated by at least 10% of the full-VL segments. In contrast, there was only one in category in this case for full-VH segment, including 90% of them. Another notable point is that the mean values of the four features (RSS, Accessibility, Disorder, Known homologs) differ significantly between full-VH and full-VL segments, even when considering a same binary class (Figure 6). In particular, (i) full-VL segments with at least one RSS contain on average fewer residues participating in a RSS than similar full-VH segments (Figure 6a); (ii) full-VL segments with accessibility less than 82.9% are more accessible to solvent than similar full-VH segments (Figure 6b); (iii) full-VL segments with disorder less than 33.4% are predicted to be more disordered than similar full-VH segments (Figure 6c); finally, the full-VL segments with at least 23.5 known homologs per site in BFD have fewer homologs than similar full-VH segments (Figure 6d).

#### 3.3.1. Full-VL Segments with AF2 Well-folded Models

**Figure 7:**
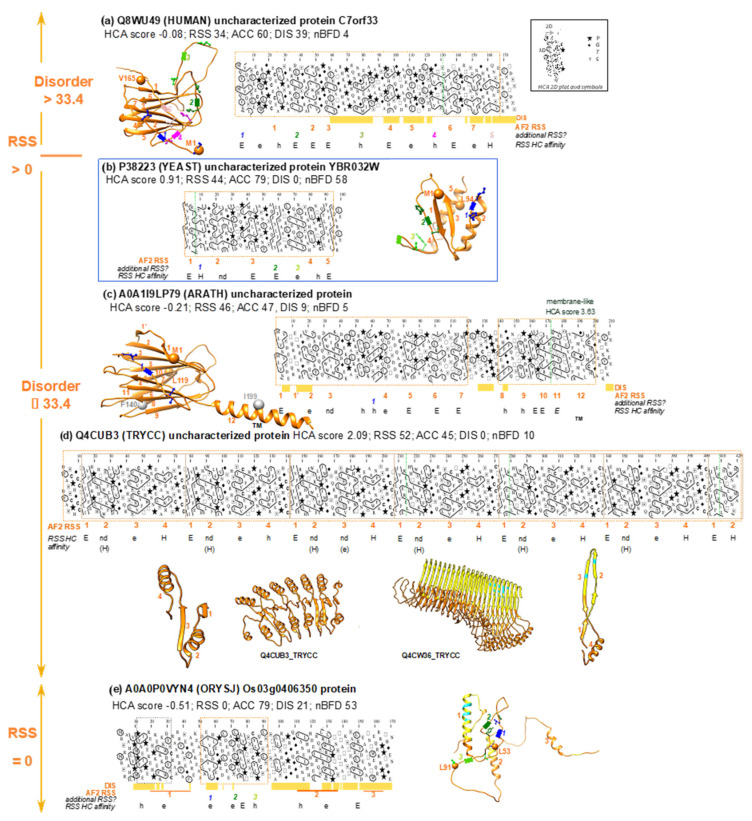
Examples of full-VL soluble-like foldable segments corresponding to folded AF2 predictions. Examples were extracted from the binary tree diagram shown in Figure 5. AF2 3D structure models, colored according to the pLDDT values, are shown, along with the positions of the first and last amino acids of the full-VL soluble-like foldable segments (orange balls). The values of the four features are indicated, along with the HCA scores. HCA plots of sequences of the full-VL soluble-like foldable segments are also shown (orange, dashed boxes). How to read sequences (1D) and secondary structures (2D) is shown in the inset, as well as the special symbols used to designate four amino acids with respect to their particular structural behavior. Regular secondary structures (RSS), as observed in the AF2 3D structure models, are designated with orange numbers, which are also reported below the HCA plot in order to indicate the correspondence with hydrophobic clusters. RSS predicted only according to the presence of hydrophobic clusters are reported in other colors, and their positions are indicated on the AF2 3D structure models (with the first and last amino acids shown in atomic details). The hydrophobic cluster affinities for RSSs, calculated using only the binary pattern information, are indicated, as extracted from HCDB v2 [36]. The upper (H,E) and lower (h,e) cases stand for strong and weak preferences, respectively. H stands for alpha-helix, E for beta-strand. Nd stands for hydrophobic clusters for which there are insufficient statistics in HCDB for the assignment of RSS affinity. TM stands for Transmembrane. IUPred2 long disorder predictions (DIS) are indicated in orange. Hydrophobic clusters corresponding to two successive regular secondary structures are broken down into their components (vertical lines). The sequence repeat in panel (**d**) is boxed on the HCA plot, whereas the basic unit of the repeat was extracted from the 3D structures (shown at the left and right ends). The 3D structure at right illustrates the AF2 prediction for a member of the same family as the protein sequence shown on the left. The blue box corresponds to the sequence included in the leaf that is the most populated in the full-VH tree shown in Figure 3. Subfigures (**a**) to (**d**) correspond to examples with RSS > 0, disorder ≥ 33.4 (**a**) or disorder < 33.4 (**b**–**d**). Subfigure (**e**) corresponds to an example without RSS.

The category that is most populated for full-VH segments, i.e., 3D models with low solvent accessibility and tight contacts between the RSS, accounts for a substantial number of full-VL cases, although not predominant (293 segments: Figure 5, blue box). These AF2 predictions correspond to well-folded 3D structures, as illustrated with the yeast uncharacterized protein YBR032W (UniProt P38223, Figure 7b, blue box). This was predicted as an alpha + beta fold, but no significant structural similarity could be detected in the PDB database by the Dali server.

Such AF2 predictions cannot be reported with high confidence for several reasons. They could correspond to the adopted structures, but represent novel folds, with amino acid contacts not yet described in the folds used for the AF2 machine learning step and insufficient depth of the multiple sequence alignment. Conversely, RSS could also be misassembled or insufficiently relative to what is happening in the actual structure.

Logically, about five times as many cases are found with a low number of BFD homologs (1274 segments: Figure 5). This reinforces the observation that while assigning a low confidence score, AF2 can propose models even when little evolutionary information is available (Figure 7c,d). A first example (UniProt A0A1I9LP79, Figure 7c) corresponds to an uncharacterized protein from *Arabidopsis thaliana*, whose 3D structure is predicted as a 12-stranded beta-sandwich. A Dali-server search in the PDB database revealed multiple hits with similar structures but with a lower strand content (Z-scores up to 6.6 and sequence identities below 15% (e.g., pdb:4q7g-A, Z-score of 6.6, 8% identity)). Examination of the HCA plot indicated that all the hydrophobic clusters match the regular secondary structures predicted by AF2. This suggests that the basic secondary structure elements are indeed present in the proposed model, arranged correctly or not. However, no conclusion can be drawn in the absence of a sufficient number of homologs (mean BFD homologs per position: 5.08, mean sequence identity > 60%). A second intriguing example is an uncharacterized protein from *Trypanosoma cruzi* (Q4CUB3), consisting of a repeated motif of 70 amino acids (mean BFD homologs per position: 10.16, identity > 80%) (Figure 7d). This is predicted to form a repeated beta-alpha-beta-alpha motif, with the two helices arranged on either side of a central beta sheet of parallel beta-strands, forming an elongated structure with a continuous hydrophobic core. A Dali search revealed structural alignments with different tandem-repeat structures (Z-scores up to 4.4, with sequences identity below 10%), belonging to distinct structural families (armadillo repeats (pdb:6dee-A, Z-score: 4.3, 7% identity), right-handed beta-helix (pdb:5zru-A, Z-score: 4.1, 3% identity; 1bhe-A, Z-score: 4.1, 5% identity), heat repeats (pdb:5loi-A, Z-score: 4.0, 9% identity)). In addition, AF2 predictions made for some homologous sequences correspond to a different repeat fold, always predicted with a low to very low level of accuracy (e.g., Q4CW36_TRYCC, >80% mean identity on the repeated sequences, AF2 prediction corresponding to a right-handed beta helix, at right on Figure 7d). This suggests that this repeat module may correspond to a novel 3D structure, which deserves to be explored experimentally.

A third example (UniProt Q8WU49, Figure 7a) illustrates a case containing amino acids predicted to be disordered, in contrast to the former. It corresponds to the uncharacterized human protein C7orf33, which is taxonomically restricted to primates (mean BFD homologs per position: 4.49, mean identity 76%). The 3D structure predicted by AF2 corresponds to a beta-sandwich, with seven strands. A Dali search yielded many results with similar structures (Z-scores up to 5.9 and sequence identities below 15% (e.g., pdb:6eon-A, Z-score 5.7, 8% identity)). Examination of the HCA plot indicated that not all the hydrophobic clusters present in the sequence correspond to the regular secondary structures predicted by AF2. Instead, there are at least five hydrophobic clusters that correspond in the AF2 model to large, unstructured coils. Many of these clusters have strong affinity for the extended (beta-strand) state, as deduced from our hydrophobic cluster dictionary [36]. This suggests that the 3D structure of this protein could incorporate these clusters as additional regular secondary structures. Alternatively, as part of this sequence is predicted to disordered by IUPred2, it is also possible that this sequence corresponds to a disordered compact domain, helping to maintain a metastable/transient interface for target recognition, as discussed for the C-terminal domain of protein 4.1G [49].

A last category of full-VL, long soluble-like foldable segments with poor solvent accessibility are the cases without RSS. Most of these cases correspond to unfolded segments in contact with other, well-folded protein regions under consideration, making them comparable to the principal category described below. However, a few cases correspond to segments that show a tendency to form a hydrophobic core without the presence of true secondary structures (see for instance the case of a protein from *Oryza sativa* in Figure 7e).

These examples indicate that such foldable domains, with very low AF2 pLDDT values but a presence of regular secondary structures interacting with each other, may correspond to original, well-folded structures. These are thus prime targets for experimental investigation, especially in the absence of sufficiently divergent homologous sequences. These include tandem repeats, which are relatively poorly represented in the PDB compared to other folds [50].

#### 3.3.2. Full-VL Segments with AF2 Unfolded Models

The most abundant category of the full-VL segments (orange boxes in Figure 5) corresponds to unfolded 3D models (encompassing more than 82.9% residues considered solvent accessible by DSSP). These are predicted as disordered or not by IUPred2 and have a low number of known homologs in BFD. This supports the general observation that VL residues are mostly associated with disorder, as no or very few unassembled RSS can be predicted by AF2. The fact that cases with few BFD homologs are about ten times more numerous than cases with a high number of BFD homologs supports the assignment of these segments to the “disorder” category, because IDR sequences are known to be less conserved. However, the HCA score values and the content in hydrophobic clusters suggest that these segments contain conditional order. Nevertheless, it cannot be ruled out that AF2 fails to predict RSS that can assemble into stable, well-folded 3D structures due to the lack of evolutionary information (or, for cases with a high number of BFD homologs, to insufficient depth of multiple sequence alignments). Such cases are referred to as “hidden” (unconditional) order. These hypotheses of conditional or unconditional order cannot be unequivocally demonstrated without the use of experimentation. Nevertheless, we give below some examples supported by experiments that confirm these hypotheses.

**Figure 8:**
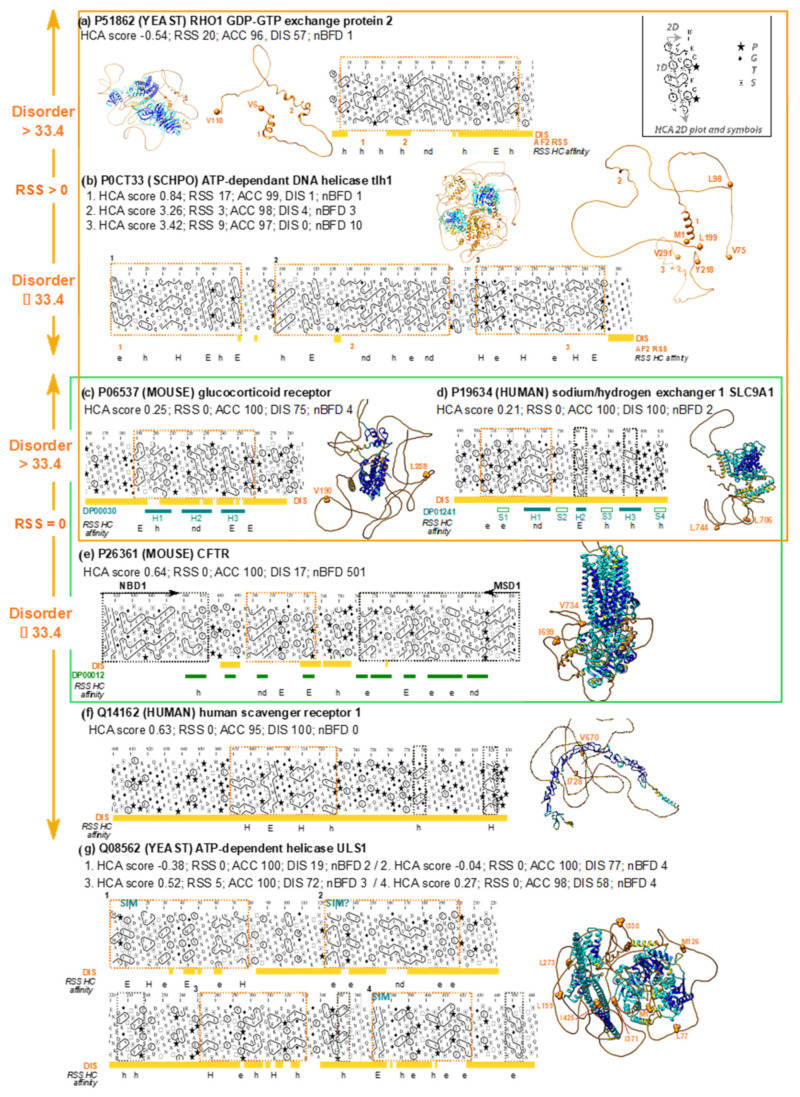
Examples of full-VL soluble-like foldable segments corresponding to unfolded AF2 predictions. See legend in Figure 7. The green box illustrates cases of disordered sequences with transient regular structures (highlighted in green below the HCA plot), documented in the DisProt database (accession number in green at left). SIM stands for Sumo-Interacting Motif. Orange boxes correspond to sequences included in the most populated leaf of the full-VL tree shown in Figure 5. Subfigures a and b correspond to examples with RSS coverage (predicted by AF2) > 0 and disorder coverage (IUPred2 predictions) > 33.4 (**a**) and ≤ 33.4 (**b**). Subfigures c to f correspond to examples RSS coverage = 0 and disorder coverage > 33.4 (**c**,**d**) and ≤ 33.4 (**e**,**f**). Subfigure (**g**) corresponds to an example with multiple full-VL soluble-like segments, some of which including SLiMs.

The first category (conditional order) is further supported by the fact that some instances are annotated in the DisProt database (Figure 8, green box). This is illustrated by a first example (Figure 8c) corresponding to a foldable segment of the mouse glucocorticoid receptor (GCR, UniProt P06537), including its core transactivation domain (DisProt DP00030, 94.2% identity with human GCR). This domain is intrinsically disordered but forms three helices that are ~30% pre-populated [51]. These three helices correspond to the positions of hydrophobic clusters on the HCA plot.

A second example (Figure 8d) is the foldable segment of the human sodium/hydrogen exchanger 1 (SLC9A1, UniProt P19634), located in its intrinsically disordered intracellular distal tail (aa 686–815, DisProt DP01241). NMR performed on two distant homologs suggested the presence of transient secondary structures and a role in molecular recognition [52]. This role was further supported by a point mutation introduced in the region that disrupts the putative binding feature and impairs trafficking to the plasma membrane [52]. These secondary structures correspond to the positions of hydrophobic clusters on the HCA plots, the first one belonging to the foldable segment described here. 

A third example (Figure 8e) is the foldable domain present in the middle of the regulatory (R) region of mouse Cystic Fibrosis Transmembrane conductance Regulator (CFTR, UniProt P26361), a chloride channel belonging to the ABC transporter superfamily (DisProt DP00012, 64% identity with human CFTR). The entire R region of CFTR is a well-known example of an intrinsically disordered sequence whose phosphorylation regulates channel activity [53]. The R region has been shown to interact with the nucleotide-binding domain 1 (NBD1) via multiple transient helices [54]. One of them is included in the foldable region considered here, which is located in the middle of the R region, while the two N- and C-terminal part of the R domain are embedded in the foldable segments of the preceding (Nucleotide Binding Domain 1) and succeeding (Membrane-Spanning Domain 1) folded domains, respectively.

Only a small fraction of the foldable segments corresponding to such AF2 predictions (i.e., low pLDDT values, no regular secondary structures, HCA scores typical of folded, soluble domains and high IUPred2 coverage) correspond to sequences included in DisProt, with experimental evidence of conditional disorder. This suggests that the remaining segments, which are numerous, may be interesting targets for experimental studies. One such example is the human scavenger receptor F member 1 (SCARF1 (SREC_HUMAN), UniProt Q14162, Figure 8f), which plays a key role in the binding and endocytosis of endogenous and exogenous ligand. The importance of SCARF1 in immunological processes was demonstrated using a SCARF1-deficient mice model, which developed systemic lupus erythematosus-like autoimmune disease [55]. A foldable segment (aa 670–728, HCA score: 0.62, IUPred2 coverage 100%) with three hydrophobic clusters typical of an alpha-beta-alpha motif can be found in its large, otherwise, intrinsically disordered cytoplasmic domain (Figure 8d), for which a role in signaling has been suggested but this function has yet to be elucidated [55]. The foldable segment highlighted here is a good candidate for further exploration of conditional order, even though this remains to be supported at the experimental level.

Short linear motifs (SLiMs) [56] are a priori excluded from this study because their lengths are below the threshold fixed here (30 amino acids) and as they often contain only a single hydrophobic cluster [30]. Such cases are associated with higher HCA scores (Bruley et al. [32]). However, some SLiMs can be embedded in larger foldable segments [30], allowing their detection in the present dataset. This is illustrated by four foldable segments detected in the N-terminal region of yeast ULS1 (UniProt Q08562, Figure 8g). This ATP-dependent helicase is required for end-joining inhibition at telomeres and interacting with the silencing regulator Sir4 [57]. SUMO-interacting motifs can be found within the first and fourth foldable segments, while a third can be suspected in the second foldable segment. The advantage of the HCA-based approach is to propose a prediction, through the boundaries of the foldable domains, of the structurally coherent neighborhood of the interacting modules, and thus highlight the sequences that confer flexibility, adaptability, and dynamic character to the IDRs.

Finally, we also observed cases of full-VL, long soluble-like foldable segments with RSS but accessible to solvent (Figure 8a,b). These can be compared to the most populated category without RSS, corresponding to either possible conditional or hidden order. Consideration of disorder predictions can help to distinguish between the different categories.

## 4. Discussion

It is now widely accepted that the low confidence structural predictions of AF2 correspond mainly to disorder [1,4,11]. In agreement with other investigations [4,5], we have recently shown that a large fraction of these sequences are indeed included in non-foldable segments as defined by pyHCA, which can therefore be considered as “full disorder” [32]. However, a substantial part of sequences with very low confidence scores in AF2 also belongs to foldable segments, in particular, those with a density in hydrophobic clusters typical of soluble domains. This led us to further study their structural characteristics, with respect to the type of order they might contain. The non-foldability/foldability of sequences is estimated by pyHCA from the sole information of a single amino acid sequence, independently of the existence of homologs, whose consideration is one of the pillars of AF2 efficiency.

The key lesson that can be drawn from our study is that the long foldable segments predicted as unfolded by AF2 with very low confidence scores (represented in the form of full-length spaghetti, like those of non-foldable segments), in fact most likely contain either conditional order or hidden, non-conditional order.

Conditional order (or disorder) can be considered as a consequence of the marginal stability of the folded state, making us aware that structure can be determined by both the sequence and the environment [58]. Here, we specifically addressed the issue of intrinsically disordered domains (IDDs), since we only considered long segments (>30 amino acids) that, moreover, are likely to correspond to homogeneous structural units, according to the definition of foldable segments. Shorter foldable segments, including a large part of MoRFs, belong to another category, characterized by higher HCA scores [32], which was not explored here. It should be noted that short linear motifs (SLiMs) can be embedded in larger foldable segments, constituting the structural unit that can modulate their interaction properties. For instance, the study of CBP interaction domain (CID) of the p160 transcriptional co-activator NCOA3 revealed that its flanking regions promote binding through short-lived, non-specific hydrophobic contacts with the partner [59]. These hydrophobic contacts are provided by hydrophobic clusters that are part of the foldable segments in which CID is included.

A recent study has shown that AF2 predicts 60% of the conditional order with high accuracy, capturing the folded state [5]. This reinforces the assumption that low scoring corresponds to full disorder. Our study provides a refined analysis and new insights for additional conditional order unidentified by AF2, which represent interesting targets worth investigating an experimental level.

The long, soluble-like full-VL foldable segments studied here may correspond to (i) cases of induced folding without the formation of a folded domain, resulting from the interaction of individual regular secondary structures with a partner, (ii) cases where a folded 3D structure is formed, dependent on the partner to be induced/stabilized, (iii) cases where a folded 3D structure is stably formed, independent of the environment (what we designate as unconditional, hidden order). This unconditional order remains completely invisible in AF2 predictions, presumably due to the lack of homologs or insufficient depth of the multiple sequence alignments used in the machine learning process.

While cases of conditional order can be supported by taking into account the DisProt database, this is not obvious for cases of hidden, non-conditional order. These indeed correspond to the unknow part of the proteomes (also described as dark proteomes). However, the HCA characteristics of these foldable segments with unfolded AF2 models (Figure 8) are comparable to those of well-folded AF2 models from the full-VL (Figure 7) and full-VH (Figure 4) categories. This supports the hypothesis that these foldable segments are still unexplored reservoirs of well-folded 3D structures. Whether these sequences correspond to true orphans, or at least taxonomically restricted genes, or whether they share distant relationships that cannot be detected by current homology detection methods is a difficult question to answer. It requires in particular novel methods going beyond sequence similarities. Recent developments for the detection of distant homologs (e.g., [60]) but also for 3D structure prediction from single protein sequences without known homologs (e.g., [61], based on the protein language model) will thus open new perspectives to decipher these cases.

The distinction between conditional and hidden, non-conditional order is not straightforward, but can be guided by taking into account current disorder predictors, in particular integrating more information on the amino acid composition. Useful information could also be given by the hydrophobic cluster composition (e.g., based on the HCA toolkit), as well as by sequences linking the hydrophobic clusters, which correspond mainly to loops.

Several hypotheses can explain the low confidence scores associated with the folded AF2 model segments. First, the proposed 3D structures should be adopted but are not yet validated by AF2 due to either: original folds/structures, the lack of representation in the databases used for learning, or an insufficient amount of homologous sequences to validate the predicted contacts. This hypothesis was recently supported in particular by Sen and colleagues [62], showing lower AF2 pLDDT values for models of sequences corresponding to unassigned domains, compared to those corresponding to CATH or Pfam entries.

Second, the proposed 3D structures should not be adopted, due to incorrect RSS assembly, with sometimes some RSSs not yet well predicted. Nevertheless, the signature of folding is there and thus, given that these proteins are largely uncharacterized, they constitute interesting targets for experimental validation, and characterization of new functions. Among these uncharacterized sequences are de novo gene candidates, as illustrated with the yeast YBR032W protein in Figure 7b [63]. Other cases are protein repeats, which are widespread periodic units involved in a wide range of functions but are generally difficult to predict due to artifacts resulting from inherent translational symmetry [64]. At the protein level, the structural mechanisms of orphan gene emergence remain to be understood. A fine-grained exploration of foldable segments within the expanding reported cases in eukaryotic proteomes (e.g., Drosophila [65], Oryza [66], Yeast [63]) would shed light on a still open debate related to the suggested disordered nature of de novo proteins, as a first structural intermediate after gene birth (e.g., [67,68,69,70,71]).

## Data Availability

The data that support the findings of the present study are available upon request from the corresponding authors E.D. and I.C.

## Supplementary Materials

The following supporting information can be downloaded at: https://www.mdpi.com/article/10.3390/biom12101467/s1, Figure S1: Full-VH binary tree diagrams per proteome; Figure S2: HCA plots of the sequences of full-VH soluble-like foldable segments; Figure S3: Full-VL binary tree diagrams per proteome; Table S1: Distribution of VH and VL residues within the long soluble-like foldable segments of the AFDB v1 dataset (21 proteomes).
