# Supplementary material for: Digging into the 3D Structure Predictions of AlphaFold2 with Low Confidence: Disorder and Beyond
Source: Biomolecules. 2022 Oct 13;12(10):1467. doi: 10.3390/biom12101467 (PMC9599455; doi:10.3390/biom12101467)
Supplement: Supplementary file 1 [file biomolecules-12-01467-s001.zip › Figure S2.pdf]

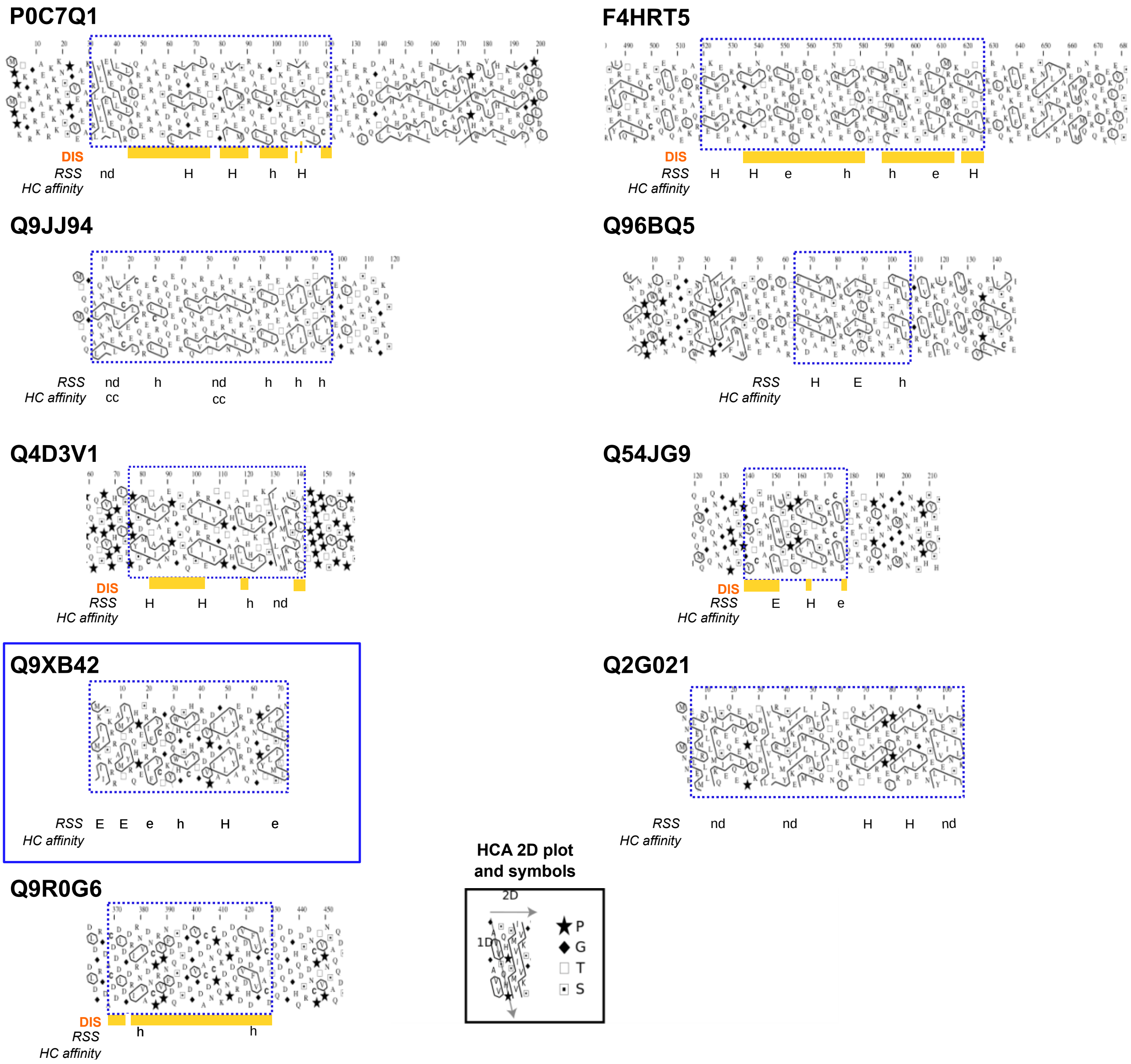

Figure S2. HCA plots of the sequences of full-VH soluble-like foldable segments (blue, dashed lines), whose AF2 3D structure models are depicted in Figure 4.

The blue box corresponds to a sequence included in the most populated leaf of the tree shown in Figure 3. How to read sequences (1D) and secondary structures (2D) are indicated in the inset, together with special symbols used to designate four amino acids with respect to their particular structural behavior.

The hydrophobic cluster affinities for RSSs, calculated using the binary pattern information only, are indicated, as extracted from HCDB v2 [36]. Upper (H,E) and lower (h,e) cases stand for strong and weak preferences, respectively. H stands for alpha-helix, E for beta-strand. Nd designates hydrophobic clusters for which no enough statistics are available in HCDB for RSS affinity assignment. Cc stands for binary patterns encountered in coiled-coils. IUPred2 long disorder predictions (DIS) are also indicated in orange.
