# Supplementary material for: Digging into the 3D Structure Predictions of AlphaFold2 with Low Confidence: Disorder and Beyond
Source: Biomolecules. 2022 Oct 13;12(10):1467. doi: 10.3390/biom12101467 (PMC9599455; doi:10.3390/biom12101467)
Supplement: Supplementary file 1 [file biomolecules-12-01467-s001.zip › Figure_S1.pdf]

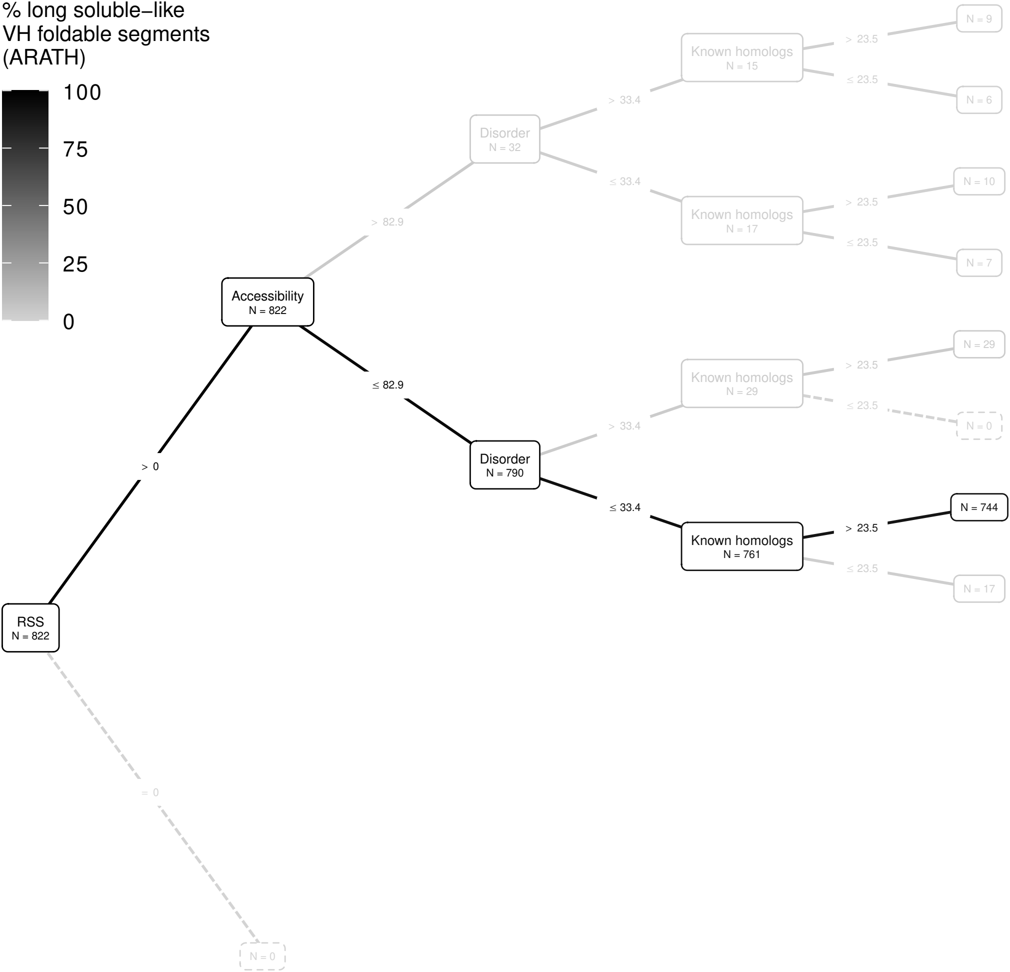

% long soluble−like VH foldable segments (ARATH)
100
75
50
25
0
RSS
N = 822
> 0
= 0
N = 0
Accessibility
N = 822
> 82.9
≤ 82.9
Disorder
N = 32
Disorder
N = 790
> 33.4
≤ 33.4
> 33.4
≤ 33.4
Known homologs
N = 15
Known homologs
N = 17
Known homologs
N = 29
Known homologs
N = 761
> 23.5
≤ 23.5
N = 9
N = 6
N = 10
N = 7
N = 29
N = 0
N = 744
N = 17

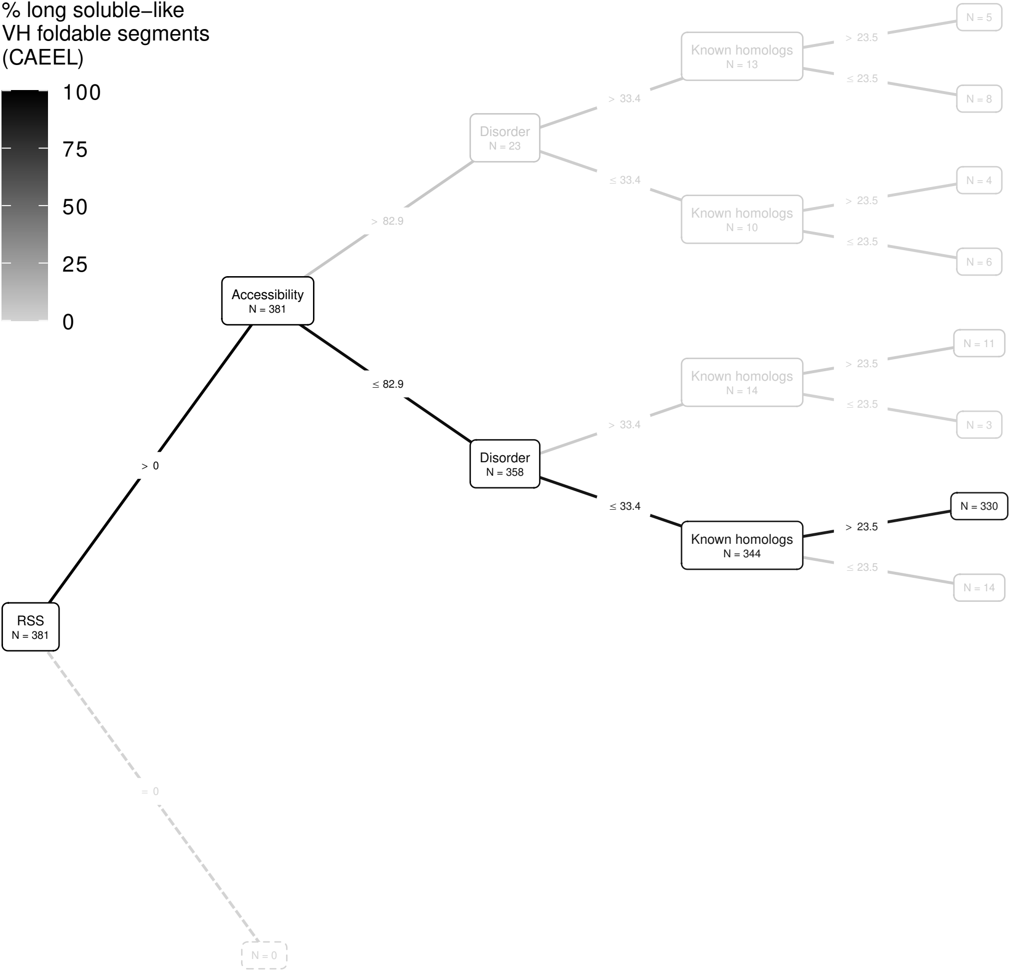

% long soluble–like VH foldable segments (CAEEL)
100
75
50
25
0
RSS
N = 381
> 0
= 0
N = 0
Accessibility
N = 381
> 82.9
≤ 82.9
Disorder
N = 23
Disorder
N = 358
> 33.4
≤ 33.4
> 33.4
≤ 33.4
Known homologs
N = 13
Known homologs
N = 10
Known homologs
N = 14
Known homologs
N = 344
> 23.5
≤ 23.5
N = 5
N = 8
N = 4
N = 6
N = 11
N = 3
N = 330
N = 14

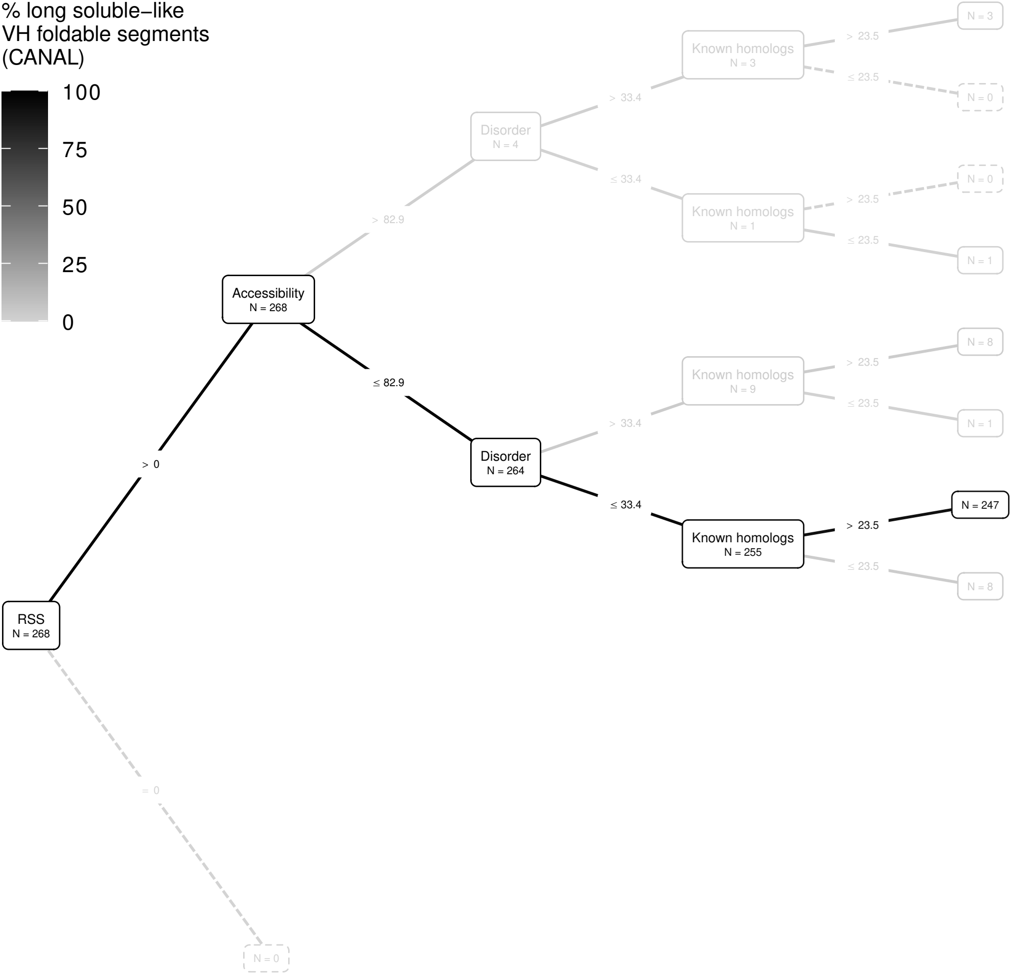
% long soluble–like VH foldable segments (CANAL)
100
75
50
25
0
RSS
N = 268
> 0
= 0
N = 0
Accessibility
N = 268
> 82.9
≤ 82.9
Disorder
N = 4
Disorder
N = 264
> 33.4
≤ 33.4
> 33.4
≤ 33.4
Known homologs
N = 3
Known homologs
N = 1
Known homologs
N = 9
Known homologs
N = 255
> 23.5
≤ 23.5
N = 3
N = 0
N = 0
N = 1
N = 8
N = 1
N = 247
N = 8

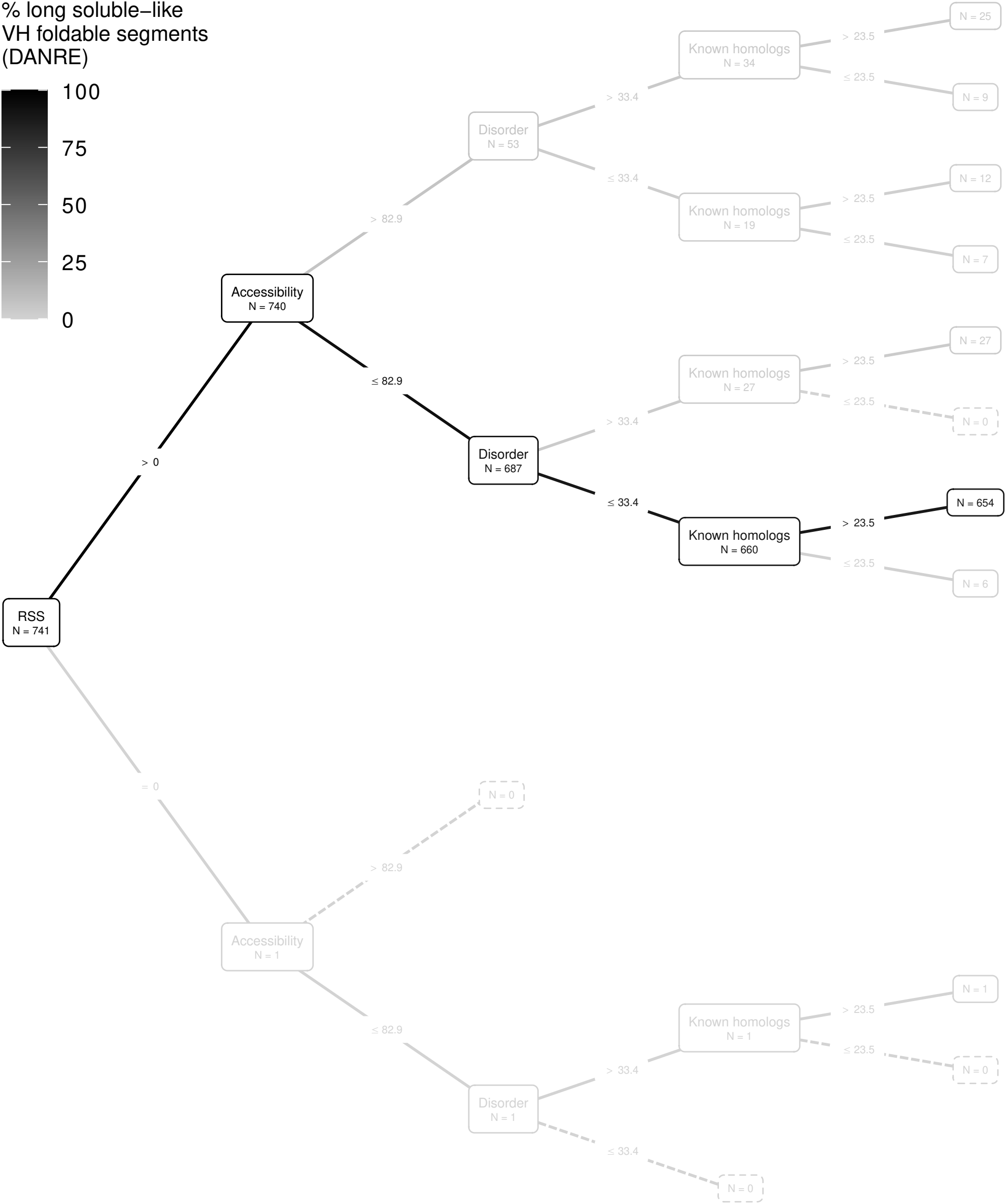

% long soluble−like VH foldable segments (DANRE)
100
75
50
25
0
RSS
N = 741
> 0
= 0
Accessibility
N = 740
> 82.9
≤ 82.9
Disorder
N = 53
Disorder
N = 687
> 33.4
≤ 33.4
Known homologs
N = 34
Known homologs
N = 19
Known homologs
N = 27
Known homologs
N = 660
> 23.5
≤ 23.5
N = 25
N = 9
N = 12
N = 7
N = 27
N = 0
N = 654
N = 6
Accessibility
N = 1
N = 0
Disorder
N = 1
Known homologs
N = 1
N = 1
N = 0
N = 0

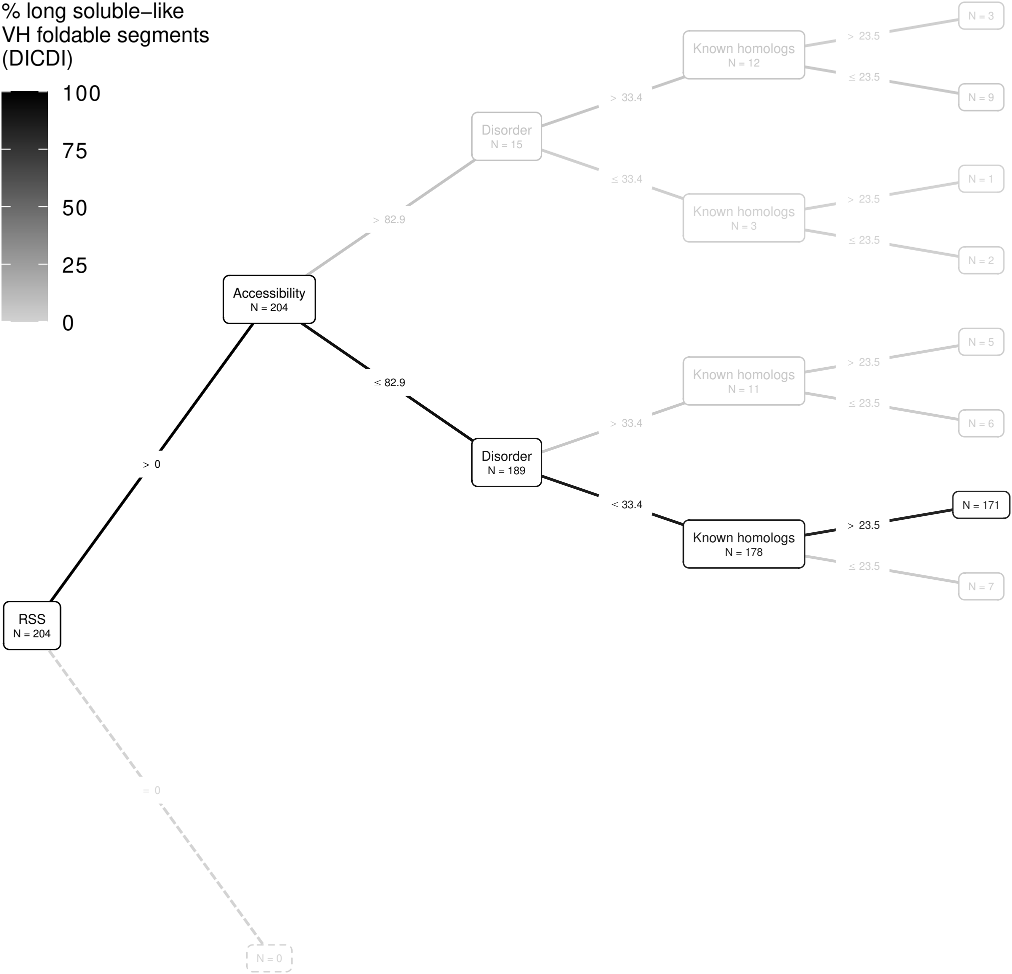
% long soluble−like
VH foldable segments
(DICDI)
100
75
50
25
0
RSS
N = 204
> 0
= 0
N = 0
Accessibility
N = 204
> 82.9
≤ 82.9
Disorder
N = 15
Disorder
N = 189
> 33.4
≤ 33.4
> 33.4
≤ 33.4
Known homologs
N = 12
Known homologs
N = 3
Known homologs
N = 11
Known homologs
N = 178
> 23.5
≤ 23.5
> 23.5
≤ 23.5
> 23.5
≤ 23.5
> 23.5
≤ 23.5
N = 3
N = 9
N = 1
N = 2
N = 5
N = 6
N = 171
N = 7

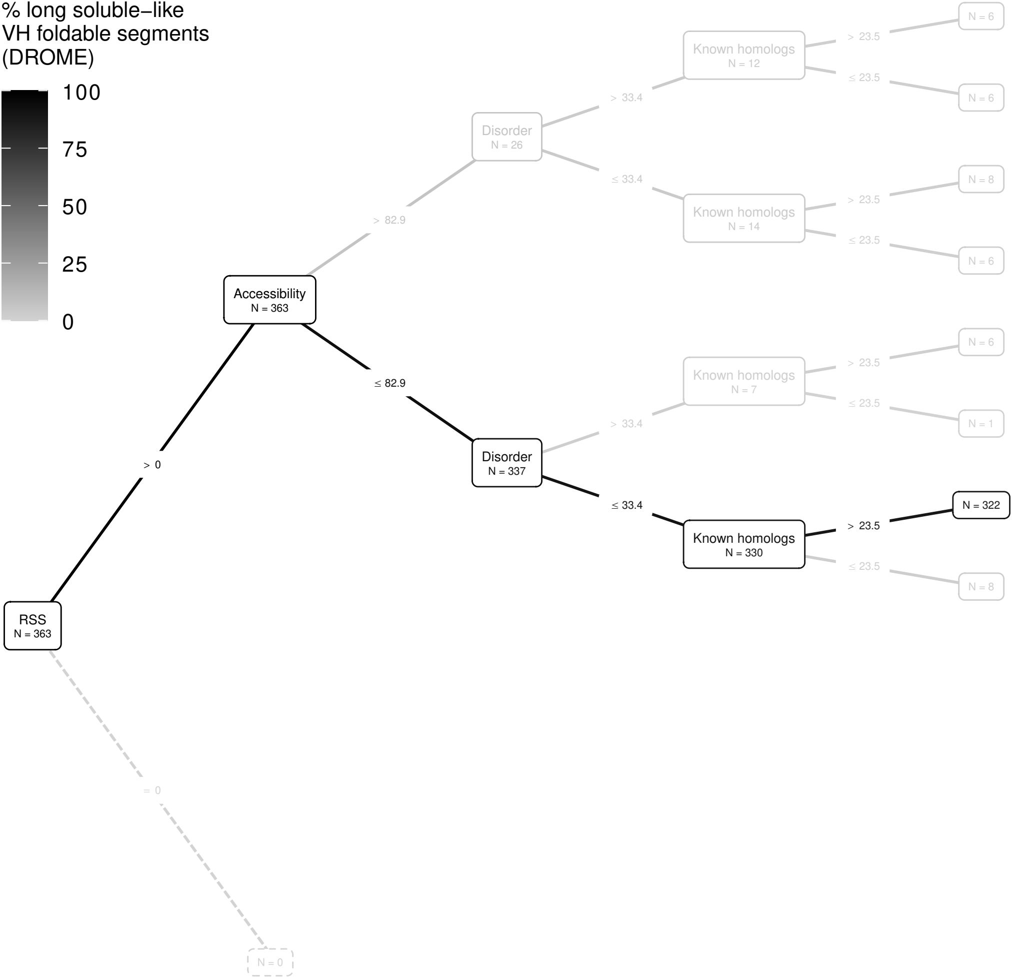
% long soluble−like
VH foldable segments
(DROME)
100
75
50
25
0
RSS
N = 363
> 0
= 0
N = 0
Accessibility
N = 363
> 82.9
≤ 82.9
Disorder
N = 26
Disorder
N = 337
> 33.4
≤ 33.4
Known homologs
N = 12
Known homologs
N = 14
Known homologs
N = 7
Known homologs
N = 330
> 23.5
≤ 23.5
N = 6
N = 6
N = 8
N = 6
N = 6
N = 1
N = 322
N = 8

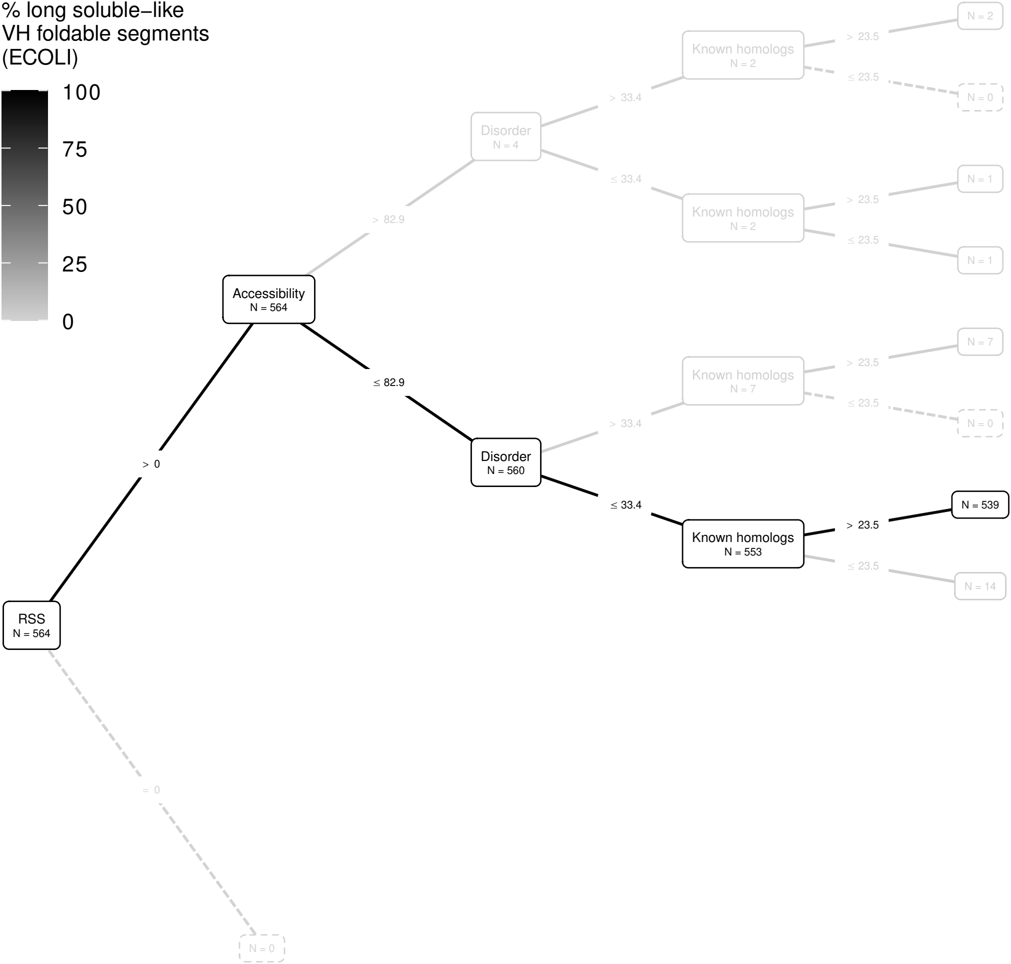

% long soluble−like
VH foldable segments
(ECOLI)
100
75
50
25
0
RSS
N = 564
> 0
= 0
N = 0
Accessibility
N = 564
> 82.9
≤ 82.9
Disorder
N = 4
Disorder
N = 560
> 33.4
≤ 33.4
> 33.4
≤ 33.4
Known homologs
N = 2
Known homologs
N = 2
Known homologs
N = 7
Known homologs
N = 553
> 23.5
≤ 23.5
N = 2
N = 0
N = 1
N = 1
N = 7
N = 0
N = 539
N = 14

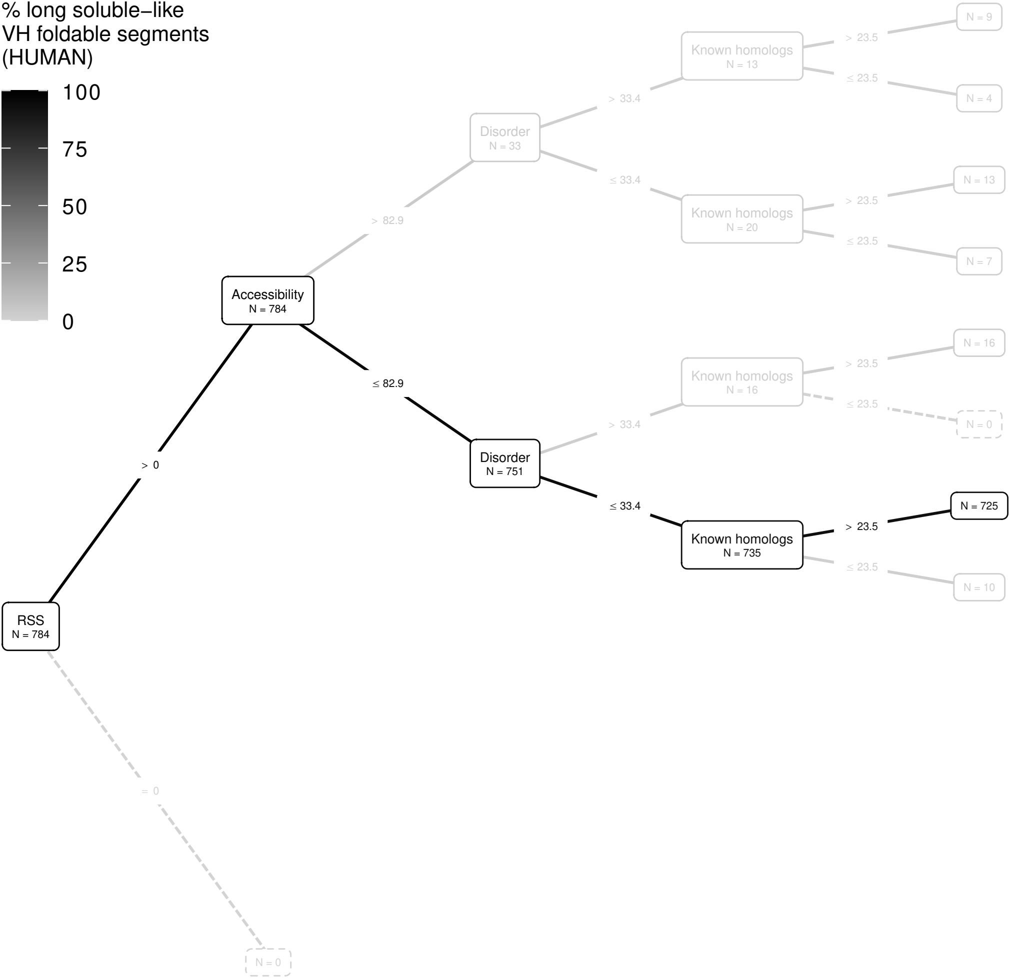
% long soluble–like
VH foldable segments
(HUMAN)
100
75
50
25
0
RSS
N = 784
> 0
= 0
N = 0
Accessibility
N = 784
> 82.9
≤ 82.9
Disorder
N = 33
Disorder
N = 751
> 33.4
≤ 33.4
> 33.4
≤ 33.4
Known homologs
N = 13
Known homologs
N = 20
Known homologs
N = 16
Known homologs
N = 735
> 23.5
≤ 23.5
N = 9
N = 4
N = 13
N = 7
N = 16
N = 0
N = 725
N = 10

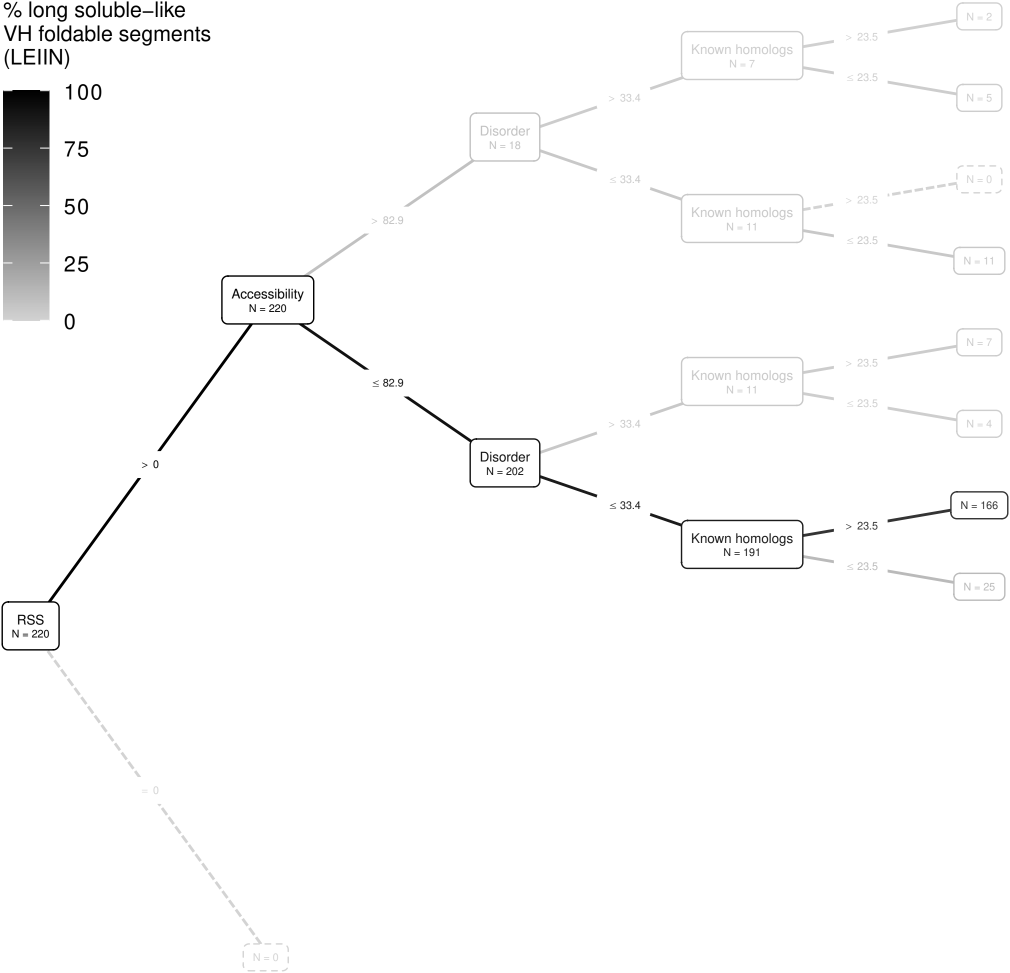
% long soluble–like
VH foldable segments
(LEIIN)
100
75
50
25
0
RSS
N = 220
> 0
= 0
N = 0
Accessibility
N = 220
> 82.9
≤ 82.9
Disorder
N = 18
Disorder
N = 202
> 33.4
≤ 33.4
> 33.4
≤ 33.4
Known homologs
N = 7
Known homologs
N = 11
Known homologs
N = 11
Known homologs
N = 191
> 23.5
≤ 23.5
> 23.5
≤ 23.5
> 23.5
≤ 23.5
> 23.5
≤ 23.5
N = 2
N = 5
N = 0
N = 11
N = 7
N = 4
N = 166
N = 25

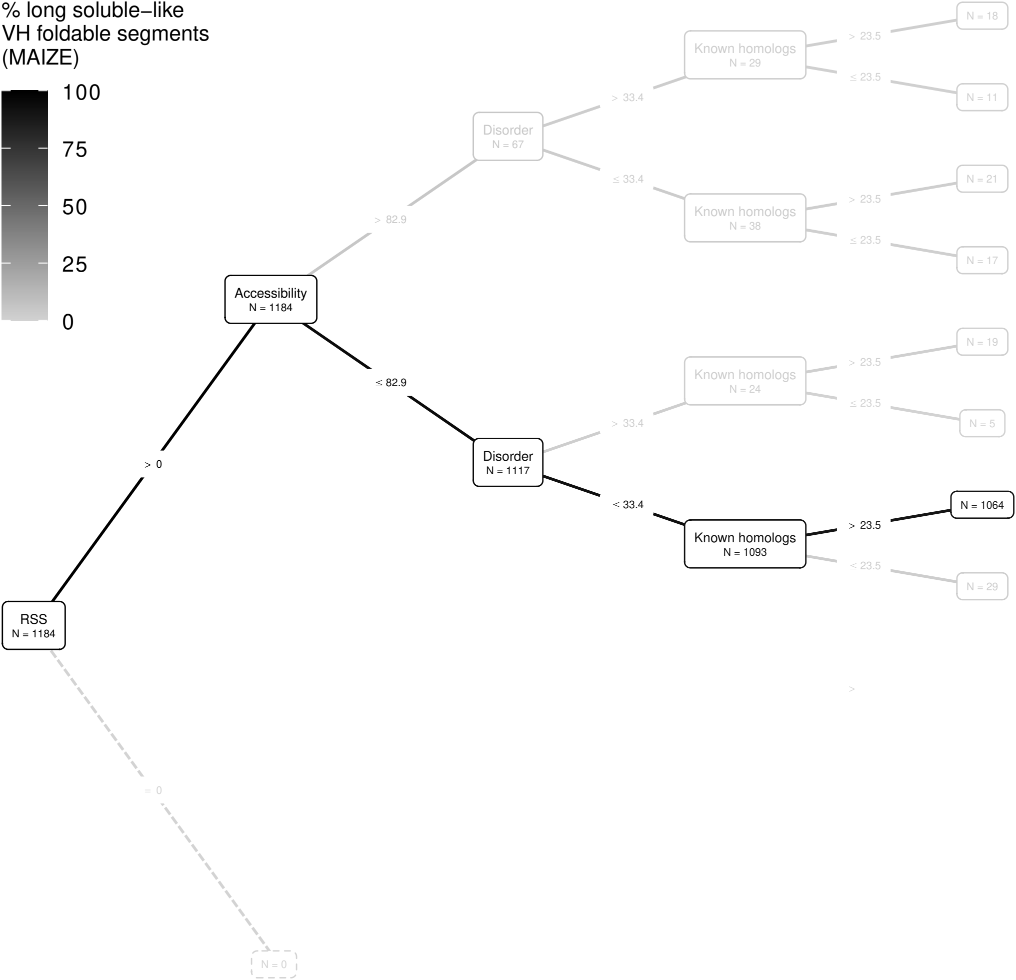

% long soluble–like
VH foldable segments
(MAIZE)
100
75
50
25
0
RSS
N = 1184
> 0
= 0
N = 0
Accessibility
N = 1184
> 82.9
≤ 82.9
Disorder
N = 67
Disorder
N = 1117
> 33.4
≤ 33.4
> 33.4
≤ 33.4
Known homologs
N = 29
Known homologs
N = 38
Known homologs
N = 24
Known homologs
N = 1093
> 23.5
≤ 23.5
N = 18
N = 11
N = 21
N = 17
N = 19
N = 5
N = 1064
N = 29

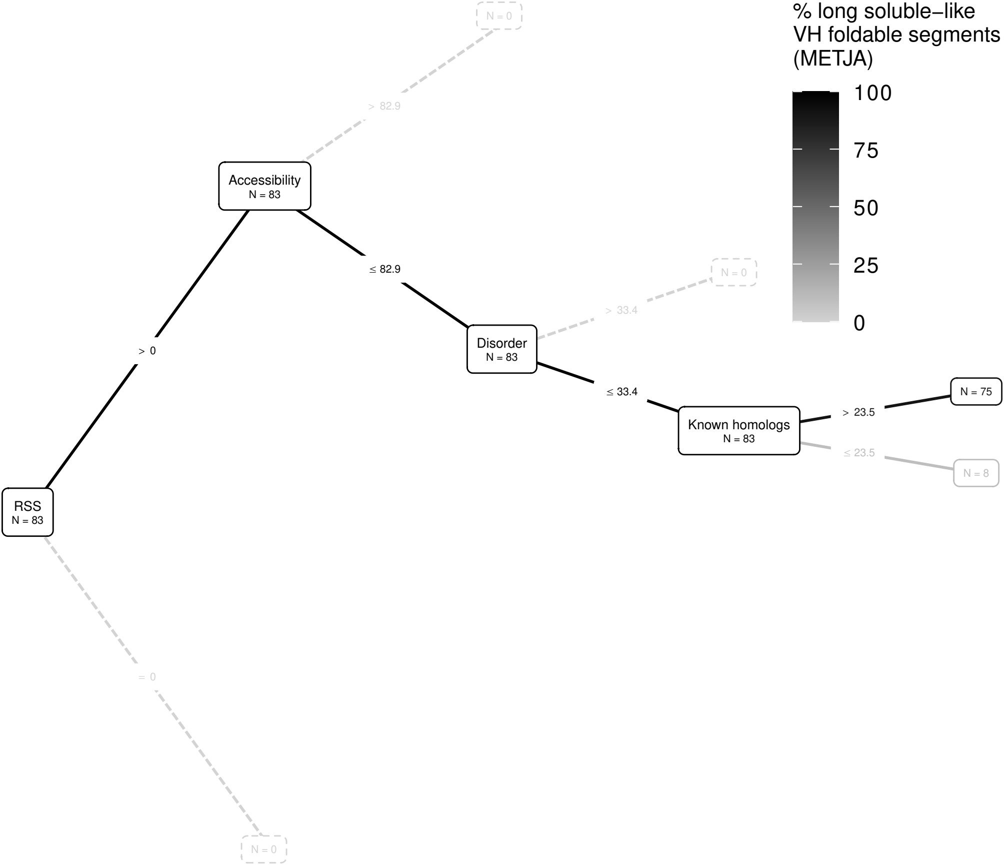

% long soluble−like VH foldable segments (METJA)
100
75
50
25
0
N = 0
> 82.9
Accessibility
N = 83
≤ 82.9
N = 0
> 33.4
> 0
Disorder
N = 83
≤ 33.4
N = 75
> 23.5
Known homologs
N = 83
≤ 23.5
N = 8
RSS
N = 83
= 0
N = 0

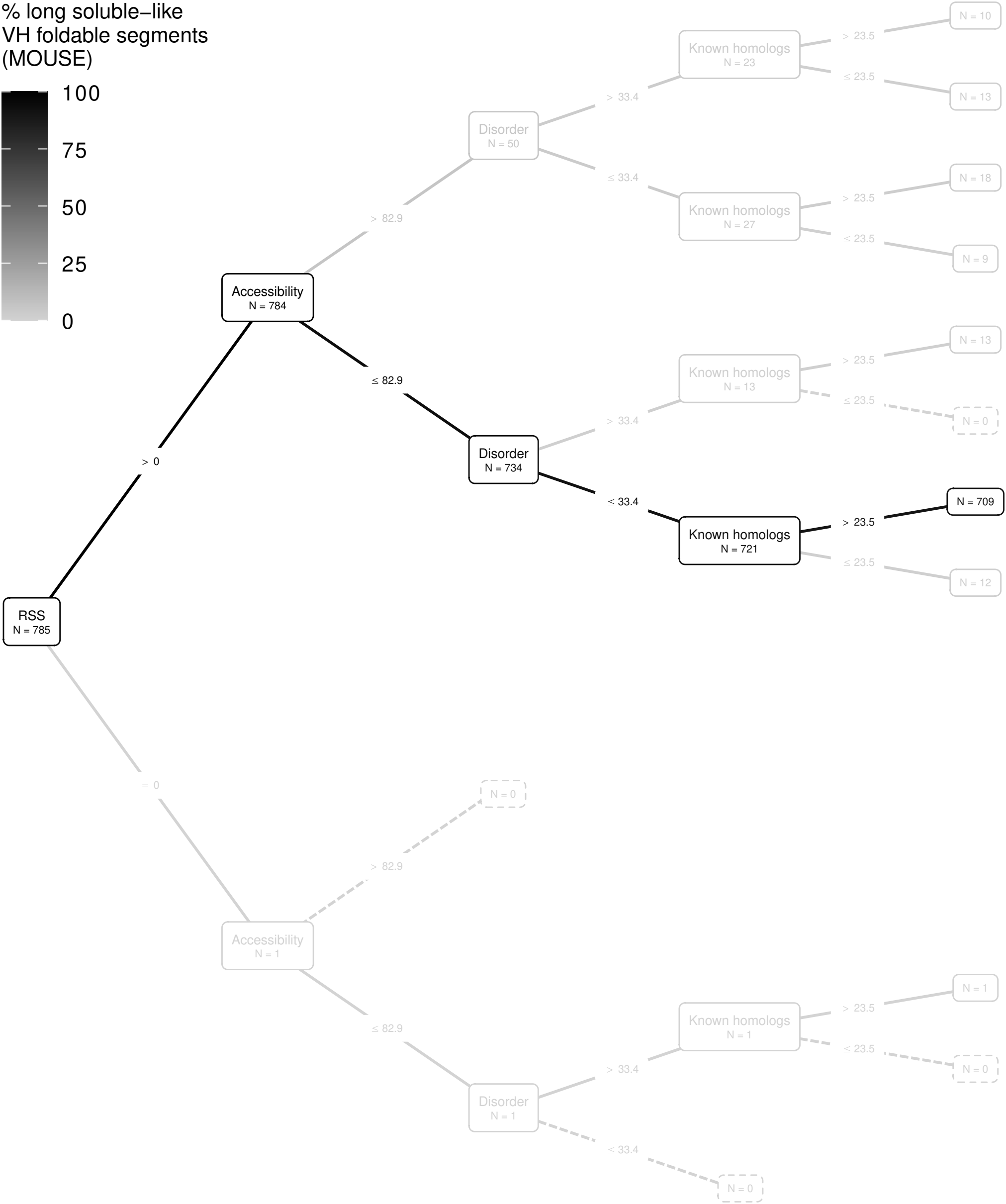

% long soluble−like
VH foldable segments
(MOUSE)
100
75
50
25
0
RSS
N = 785
> 0
= 0
Accessibility
N = 784
> 82.9
≤ 82.9
Disorder
N = 50
Disorder
N = 734
> 33.4
≤ 33.4
Known homologs
N = 23
Known homologs
N = 27
Known homologs
N = 13
Known homologs
N = 721
> 23.5
≤ 23.5
N = 10
N = 13
N = 18
N = 9
N = 13
N = 0
N = 709
N = 12
Accessibility
N = 1
N = 0
Disorder
N = 1
Known homologs
N = 1
N = 1
N = 0
N = 0

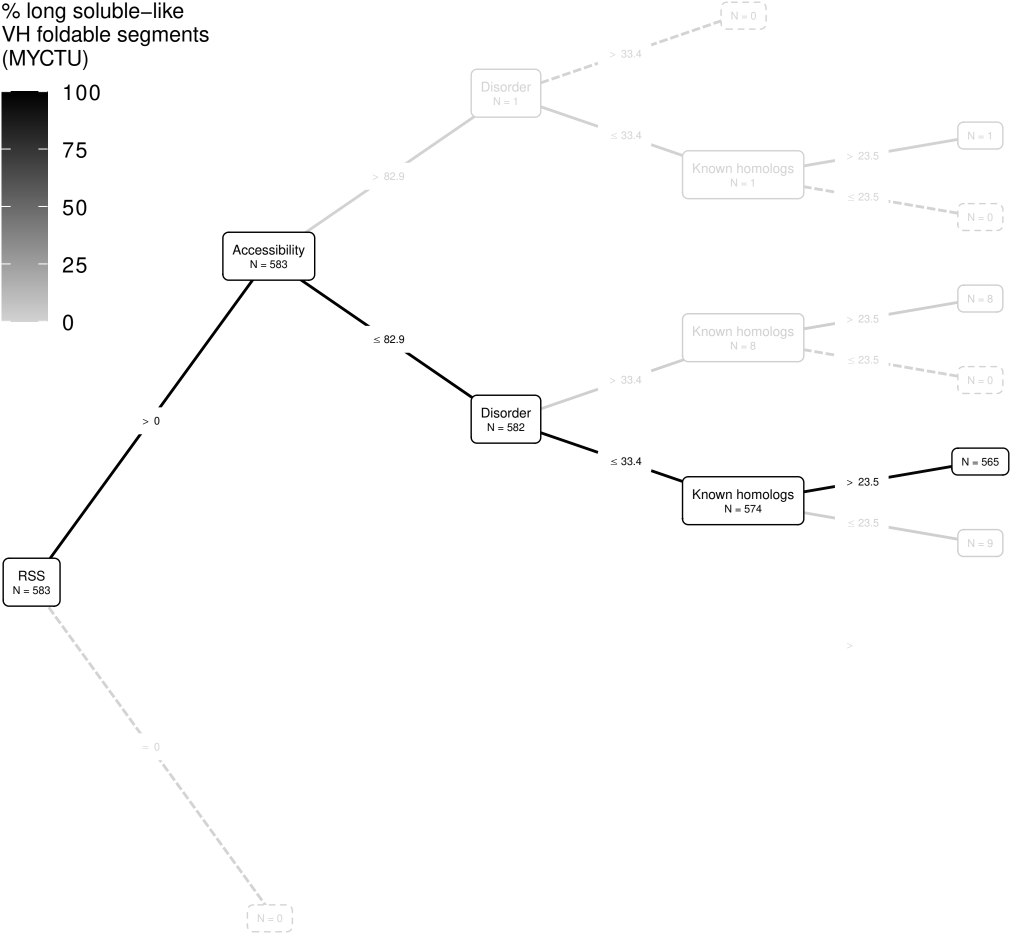
% long soluble–like
VH foldable segments
(MYCTU)
100
75
50
25
0
RSS
N = 583
> 0
= 0
N = 0
Accessibility
N = 583
> 82.9
≤ 82.9
Disorder
N = 1
> 33.4
N = 0
≤ 33.4
Known homologs
N = 1
> 23.5
N = 1
≤ 23.5
N = 0
Disorder
N = 582
> 33.4
Known homologs
N = 8
> 23.5
N = 8
≤ 23.5
N = 0
≤ 33.4
Known homologs
N = 574
> 23.5
N = 565
≤ 23.5
N = 9
>

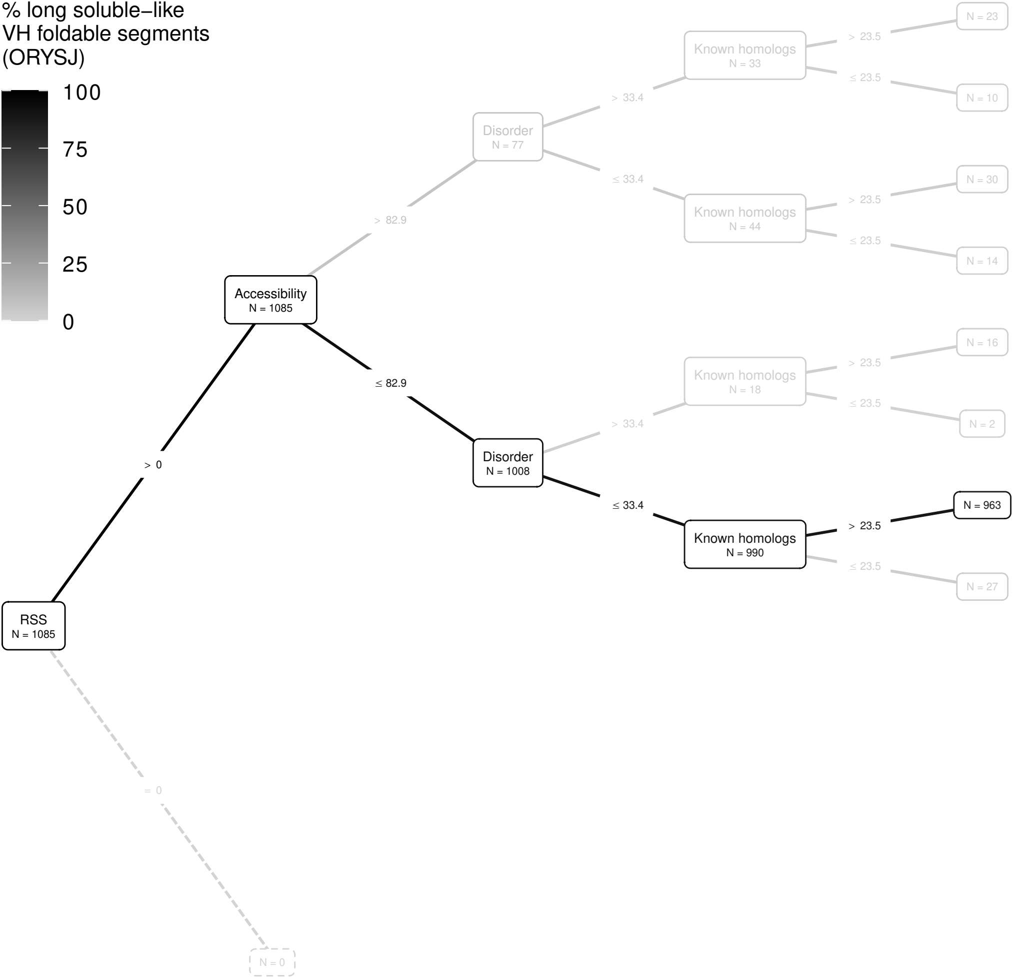
% long soluble–like
VH foldable segments
(ORYSJ)
100
75
50
25
0
RSS
N = 1085
> 0
= 0
N = 0
Accessibility
N = 1085
> 82.9
≤ 82.9
Disorder
N = 77
Disorder
N = 1008
> 33.4
≤ 33.4
> 33.4
≤ 33.4
Known homologs
N = 33
Known homologs
N = 44
Known homologs
N = 18
Known homologs
N = 990
> 23.5
≤ 23.5
N = 23
N = 10
N = 30
N = 14
N = 16
N = 2
N = 963
N = 27

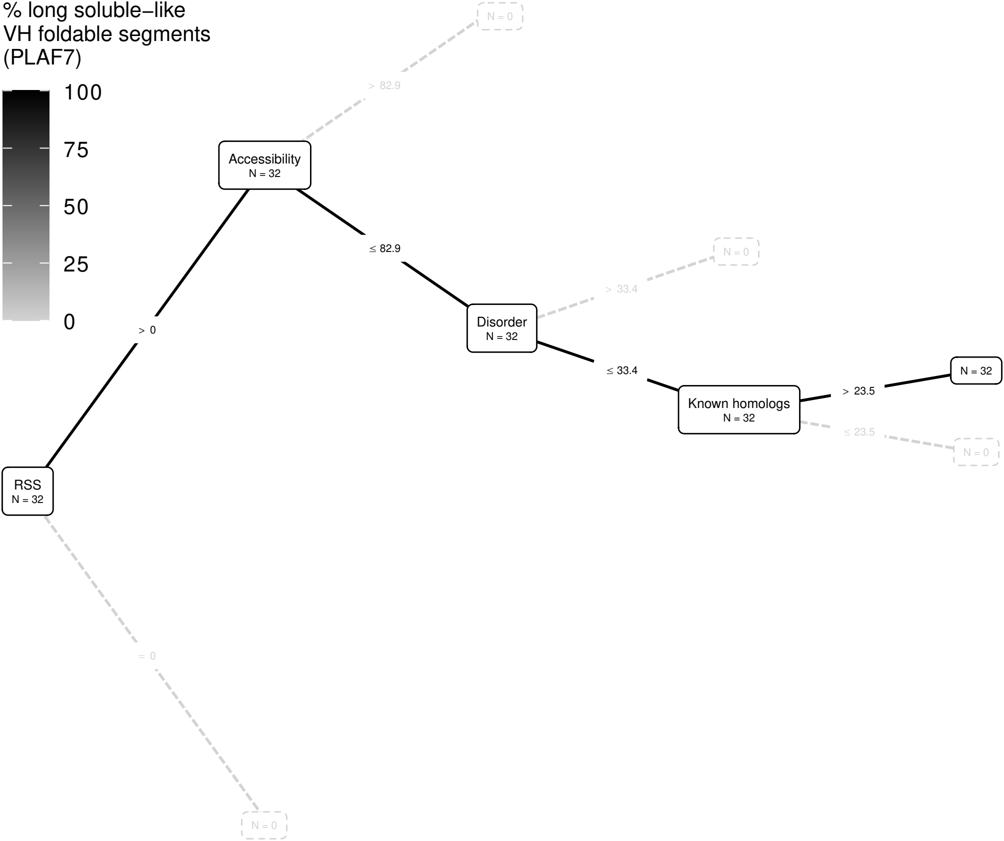

% long soluble−like
VH foldable segments
(PLAF7)
100
75
50
25
0
RSS
N = 32
> 0
= 0
N = 0
Accessibility
N = 32
> 82.9
N = 0
≤ 82.9
Disorder
N = 32
> 33.4
N = 0
≤ 33.4
Known homologs
N = 32
> 23.5
N = 32
≤ 23.5
N = 0

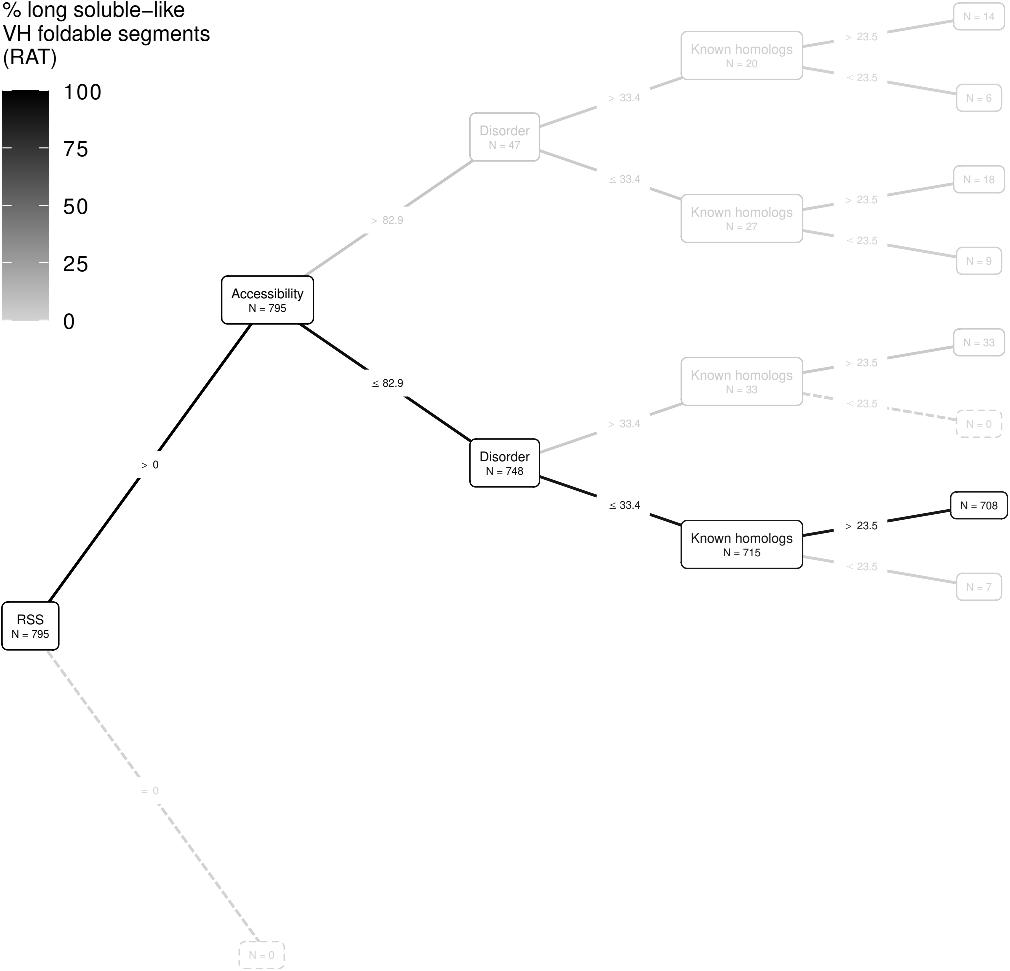
% long soluble–like
VH foldable segments
(RAT)
100
75
50
25
0
RSS
N = 795
> 0
= 0
N = 0
Accessibility
N = 795
> 82.9
≤ 82.9
Disorder
N = 47
Disorder
N = 748
> 33.4
≤ 33.4
> 33.4
≤ 33.4
Known homologs
N = 20
Known homologs
N = 27
Known homologs
N = 33
Known homologs
N = 715
> 23.5
≤ 23.5
> 23.5
≤ 23.5
> 23.5
≤ 23.5
> 23.5
≤ 23.5
N = 14
N = 6
N = 18
N = 9
N = 33
N = 0
N = 708
N = 7

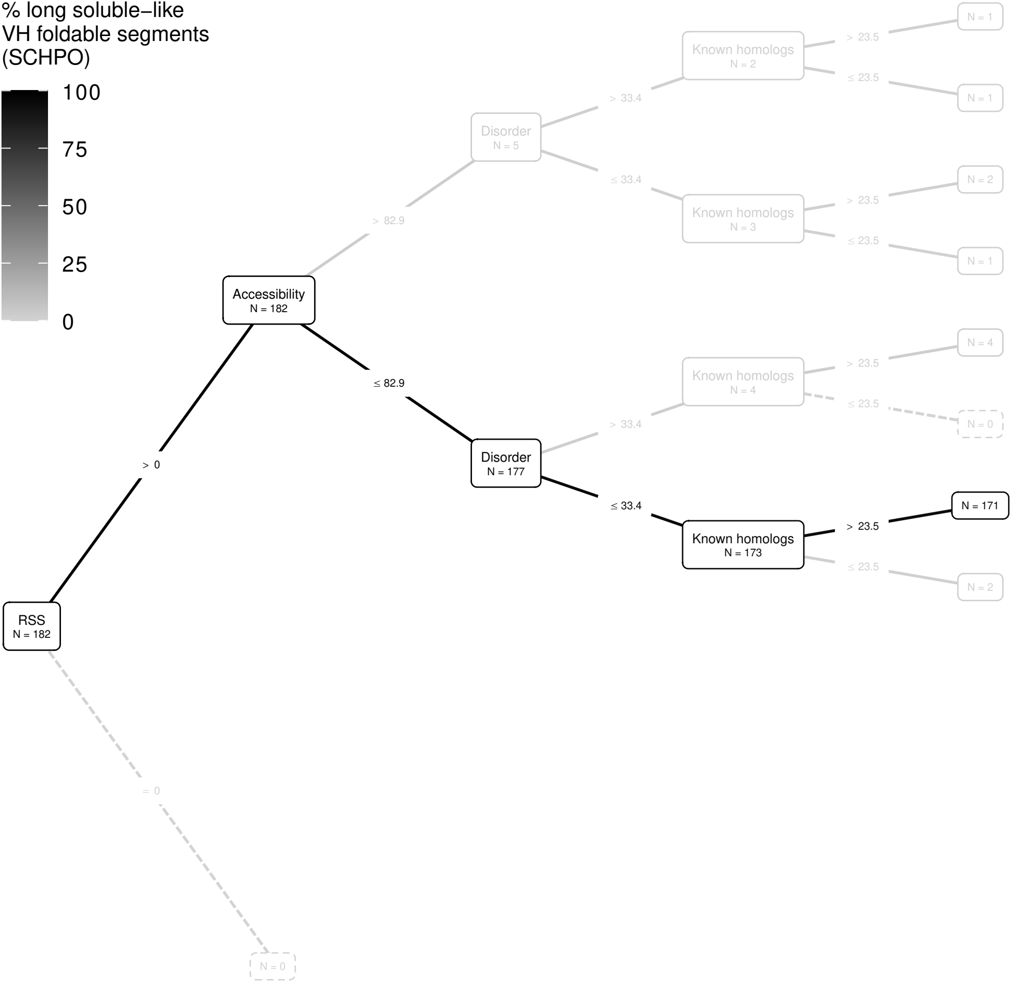

% long soluble–like
VH foldable segments
(SCHPO)
100
75
50
25
0
RSS
N = 182
> 0
= 0
N = 0
Accessibility
N = 182
> 82.9
≤ 82.9
Disorder
N = 5
Disorder
N = 177
> 33.4
≤ 33.4
> 33.4
≤ 33.4
Known homologs
N = 2
Known homologs
N = 3
Known homologs
N = 4
Known homologs
N = 173
> 23.5
≤ 23.5
N = 1
N = 1
N = 2
N = 1
N = 4
N = 0
N = 171
N = 2

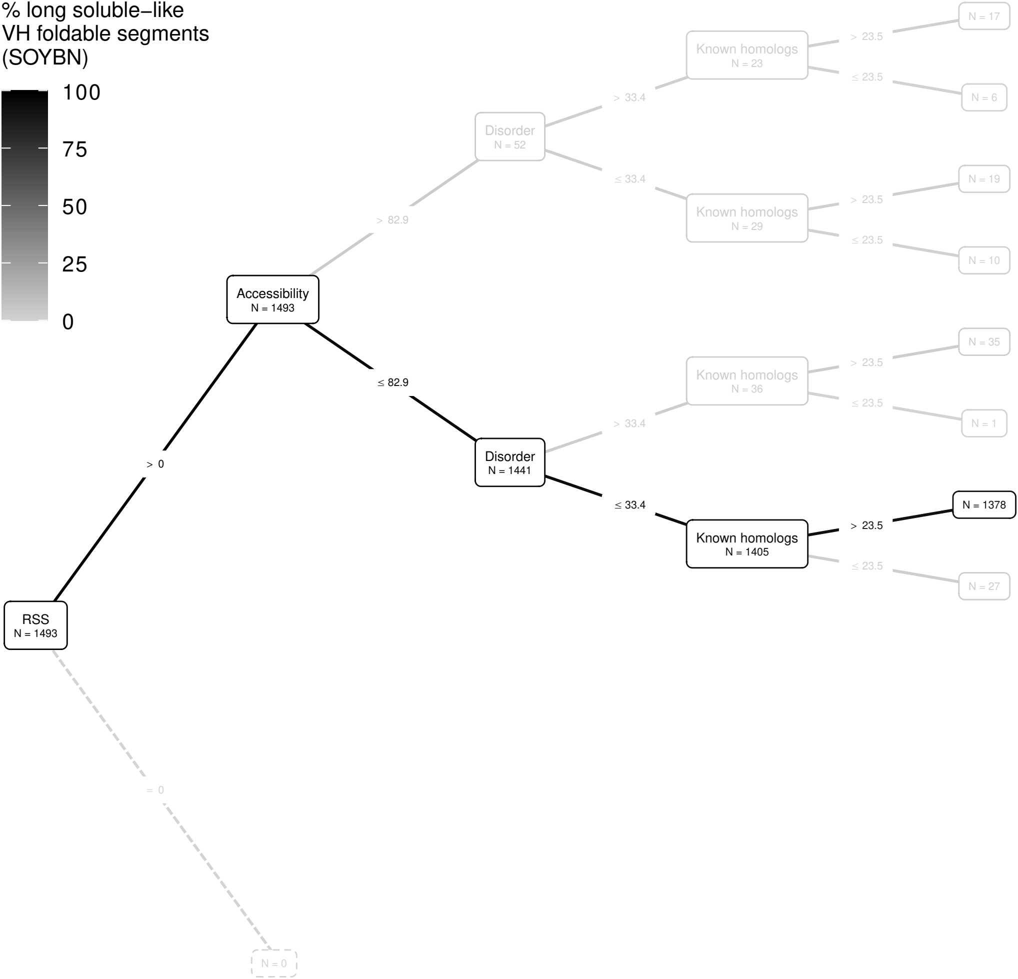

% long soluble–like
VH foldable segments
(SOYBN)
100
75
50
25
0
RSS
N = 1493
> 0
= 0
N = 0
Accessibility
N = 1493
> 82.9
≤ 82.9
Disorder
N = 52
Disorder
N = 1441
> 33.4
≤ 33.4
> 33.4
≤ 33.4
Known homologs
N = 23
Known homologs
N = 29
Known homologs
N = 36
Known homologs
N = 1405
> 23.5
≤ 23.5
N = 17
N = 6
N = 19
N = 10
N = 35
N = 1
N = 1378
N = 27

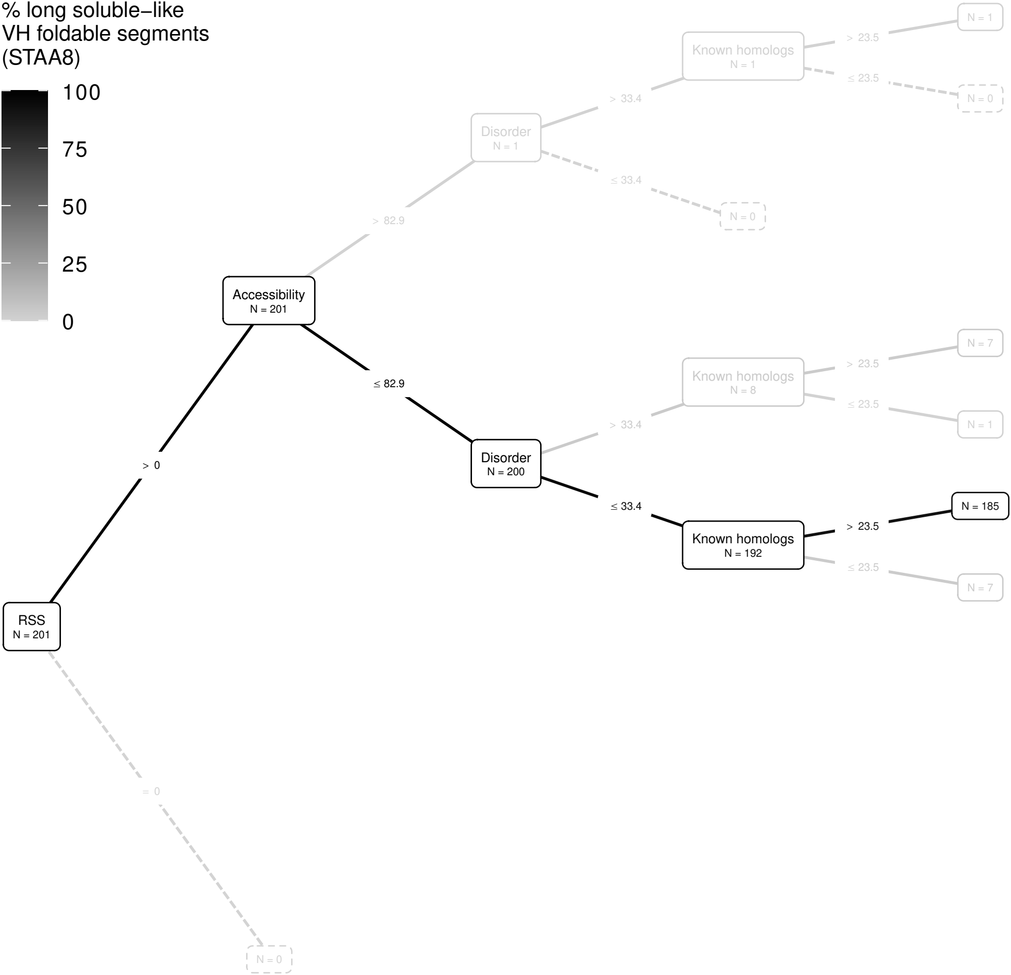

% long soluble–like
VH foldable segments
(STAA8)
100
75
50
25
0
RSS
N = 201
> 0
= 0
N = 0
Accessibility
N = 201
> 82.9
≤ 82.9
Disorder
N = 1
> 33.4
≤ 33.4
Known homologs
N = 1
> 23.5
≤ 23.5
N = 1
N = 0
N = 0
Disorder
N = 200
> 33.4
≤ 33.4
Known homologs
N = 8
> 23.5
≤ 23.5
N = 7
N = 1
Known homologs
N = 192
> 23.5
≤ 23.5
N = 185
N = 7

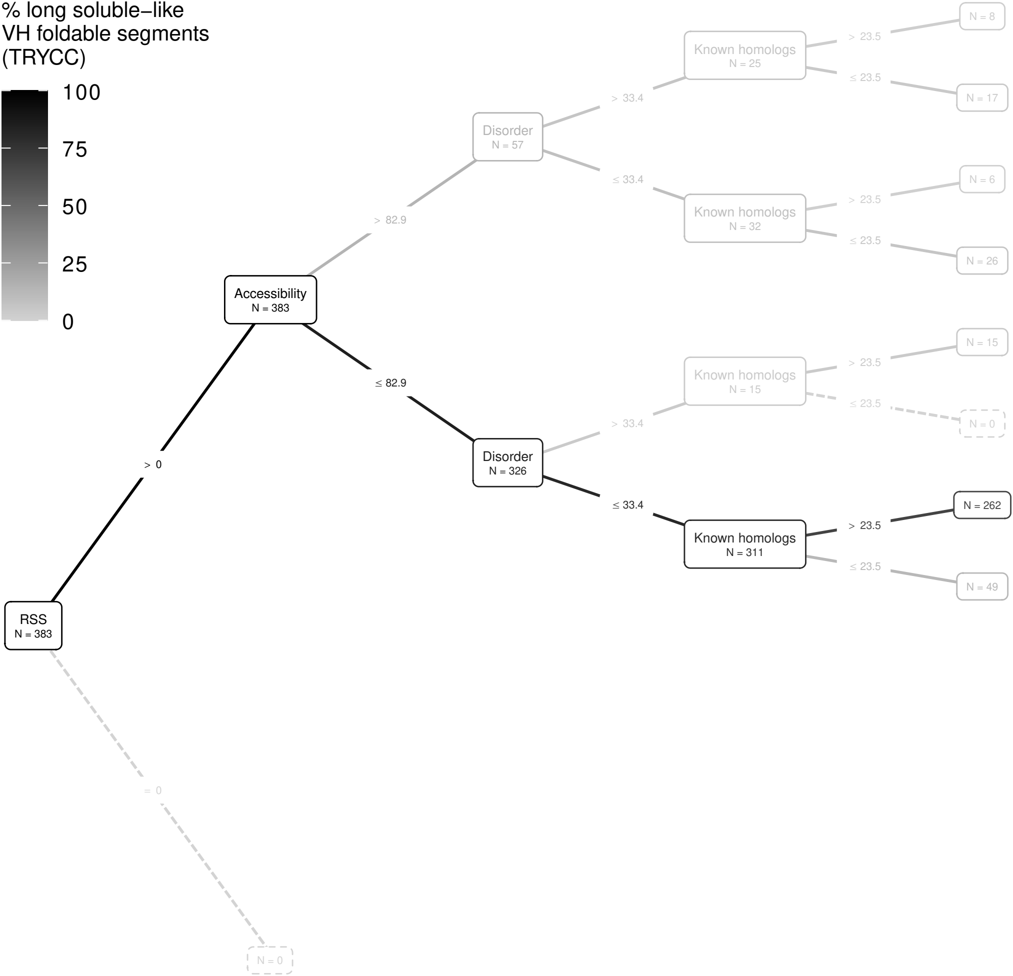
% long soluble–like VH foldable segments (TRYCC)
100
75
50
25
0
RSS
N = 383
> 0
= 0
N = 0
Accessibility
N = 383
> 82.9
≤ 82.9
Disorder
N = 57
Disorder
N = 326
> 33.4
≤ 33.4
> 33.4
≤ 33.4
Known homologs
N = 25
Known homologs
N = 32
Known homologs
N = 15
Known homologs
N = 311
> 23.5
≤ 23.5
> 23.5
≤ 23.5
> 23.5
≤ 23.5
> 23.5
≤ 23.5
N = 8
N = 17
N = 6
N = 26
N = 15
N = 0
N = 262
N = 49

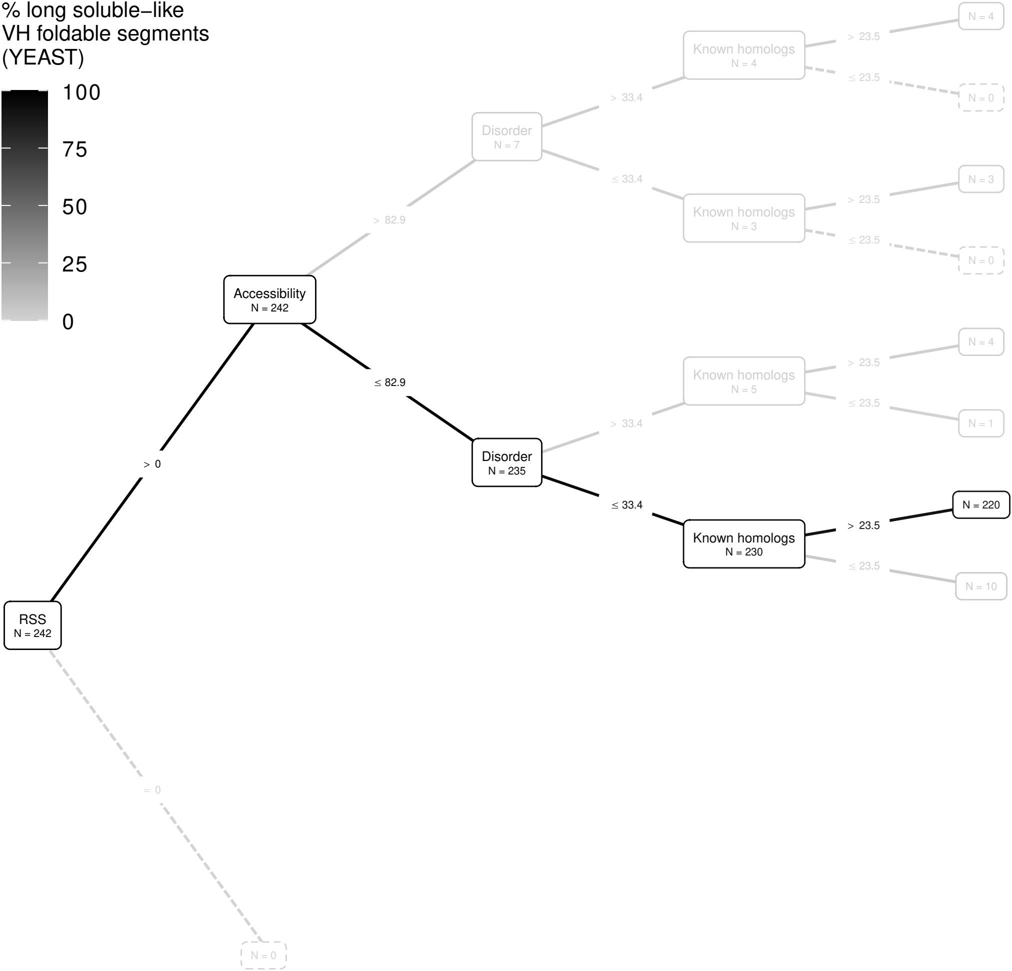
% long soluble−like
VH foldable segments
(YEAST)
100
75
50
25
0
RSS
N = 242
> 0
= 0
N = 0
Accessibility
N = 242
> 82.9
≤ 82.9
Disorder
N = 7
Disorder
N = 235
> 33.4
≤ 33.4
> 33.4
≤ 33.4
Known homologs
N = 4
Known homologs
N = 3
Known homologs
N = 5
Known homologs
N = 230
> 23.5
≤ 23.5
> 23.5
≤ 23.5
> 23.5
≤ 23.5
> 23.5
≤ 23.5
N = 4
N = 0
N = 3
N = 0
N = 4
N = 1
N = 220
N = 10
