# Supplementary material for: Digging into the 3D Structure Predictions of AlphaFold2 with Low Confidence: Disorder and Beyond
Source: Biomolecules. 2022 Oct 13;12(10):1467. doi: 10.3390/biom12101467 (PMC9599455; doi:10.3390/biom12101467)
Supplement: Supplementary file 1 [file biomolecules-12-01467-s001.zip › Figure_S3.pdf]

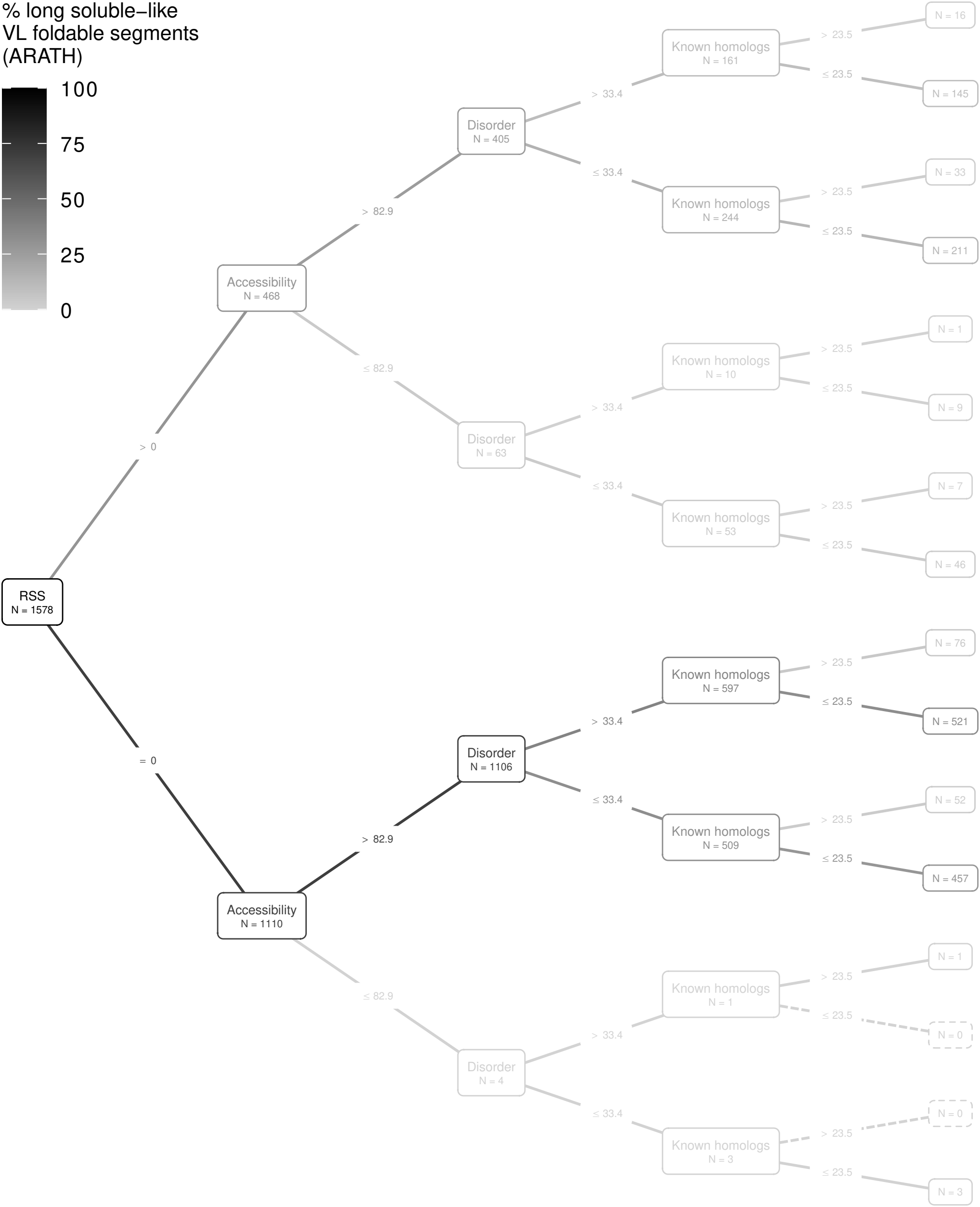

% long soluble−like
VL foldable segments
(ARATH)
100
75
50
25
0
RSS
N = 1578
> 0
= 0
Accessibility
N = 468
Accessibility
N = 1110
> 82.9
≤ 82.9
Disorder
N = 405
Disorder
N = 63
Disorder
N = 1106
Disorder
N = 4
> 33.4
≤ 33.4
Known homologs
N = 161
Known homologs
N = 244
Known homologs
N = 10
Known homologs
N = 53
Known homologs
N = 597
Known homologs
N = 509
Known homologs
N = 1
Known homologs
N = 3
> 23.5
≤ 23.5
N = 16
N = 145
N = 33
N = 211
N = 1
N = 9
N = 7
N = 46
N = 76
N = 521
N = 52
N = 457
N = 1
N = 0
N = 0
N = 3

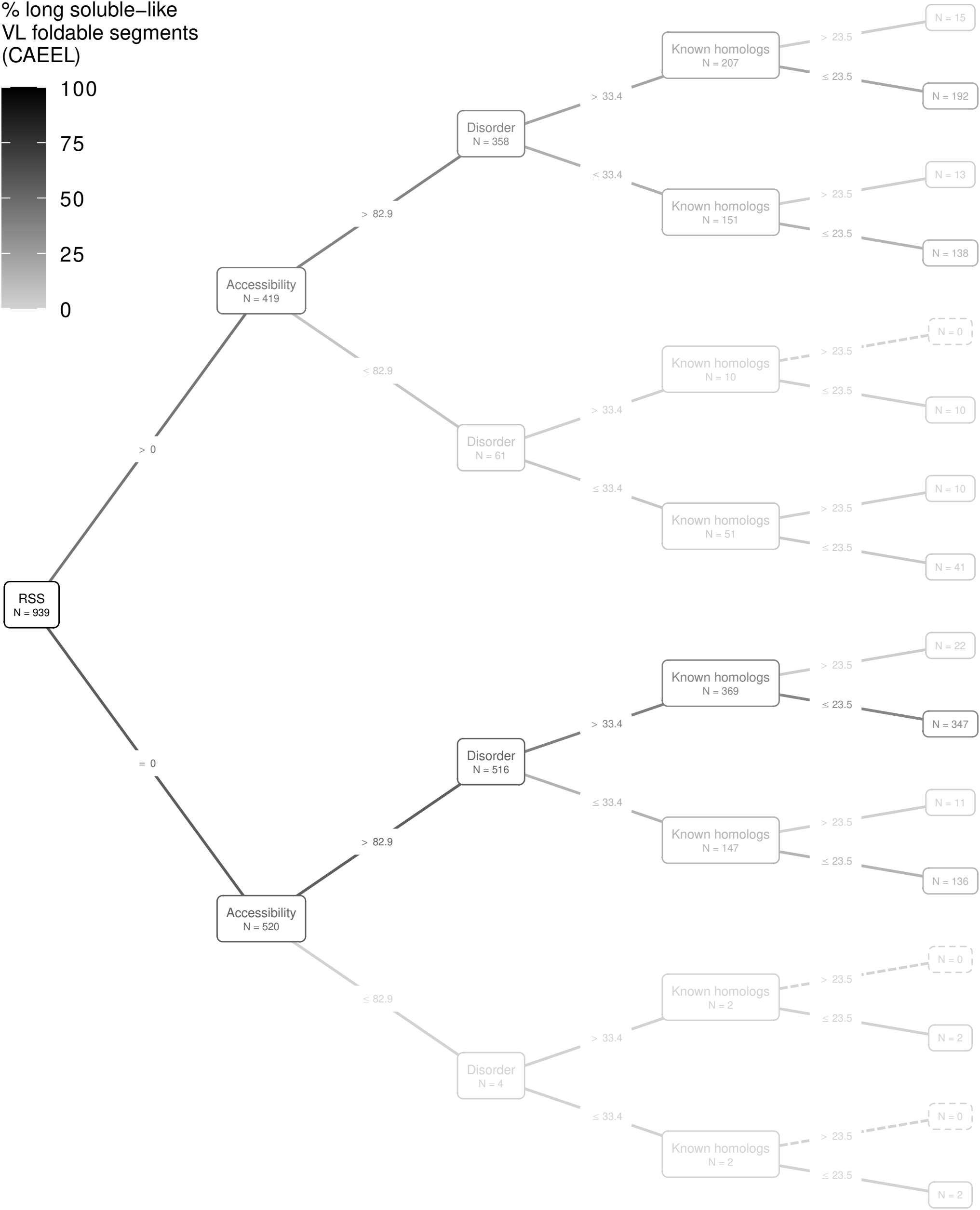
% long soluble−like VL foldable segments (CAEEL)
100
75
50
25
0
RSS N = 939
> 0
= 0
Accessibility N = 419
Accessibility N = 520
> 82.9
≤ 82.9
Disorder N = 358
Disorder N = 61
Disorder N = 516
Disorder N = 4
> 33.4
≤ 33.4
Known homologs N = 207
Known homologs N = 151
Known homologs N = 10
Known homologs N = 51
Known homologs N = 369
Known homologs N = 147
Known homologs N = 2
Known homologs N = 2
> 23.5
≤ 23.5
N = 15
N = 192
N = 13
N = 138
N = 0
N = 10
N = 10
N = 41
N = 22
N = 347
N = 11
N = 136
N = 0
N = 2
N = 0
N = 2

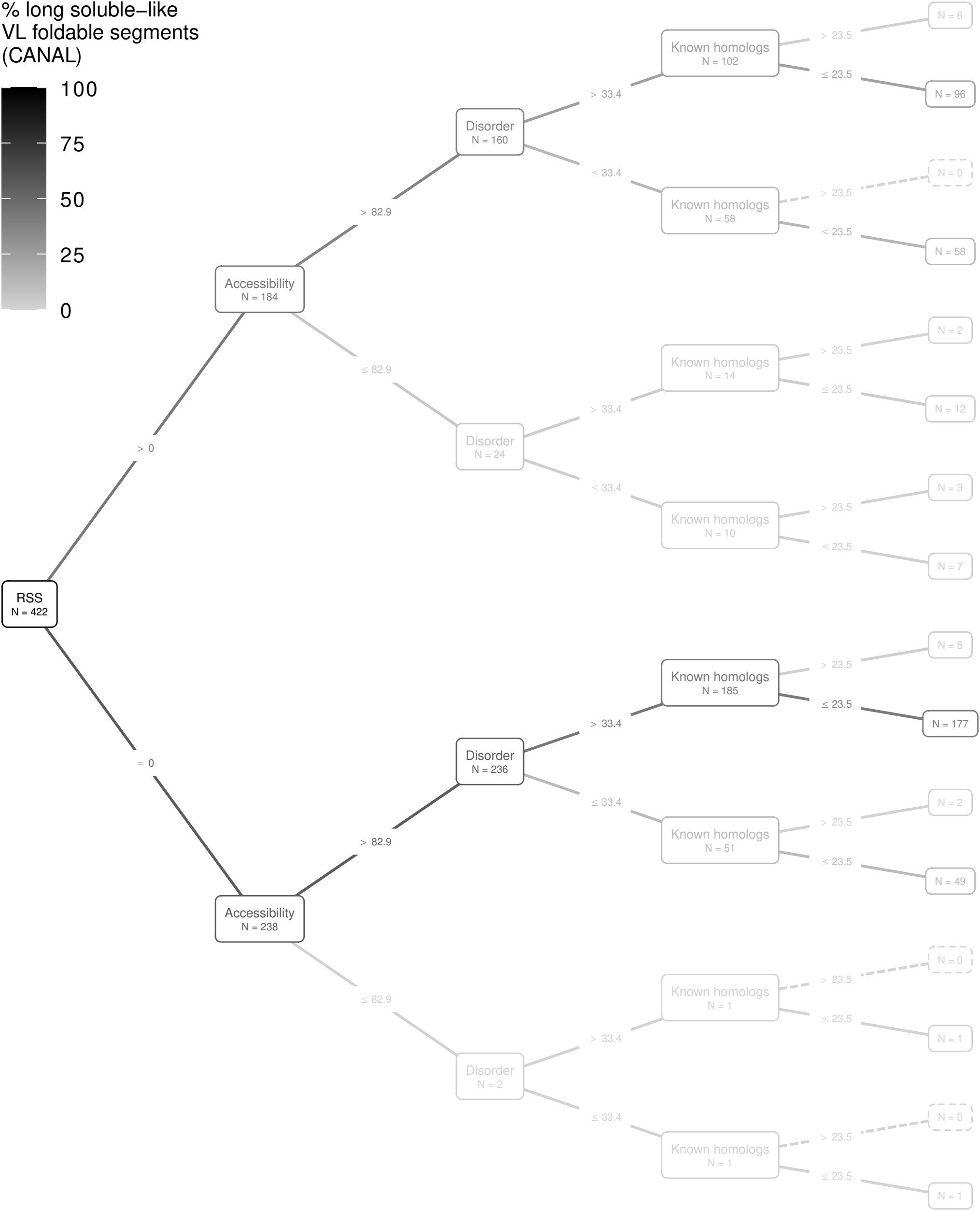
% long soluble−like
VL foldable segments
(CANAL)
100
75
50
25
0
RSS
N = 422
> 0
= 0
Accessibility
N = 184
Accessibility
N = 238
> 82.9
≤ 82.9
> 82.9
≤ 82.9
Disorder
N = 160
Disorder
N = 24
Disorder
N = 236
Disorder
N = 2
> 33.4
≤ 33.4
> 33.4
≤ 33.4
> 33.4
≤ 33.4
> 33.4
≤ 33.4
Known homologs
N = 102
Known homologs
N = 58
Known homologs
N = 14
Known homologs
N = 10
Known homologs
N = 185
Known homologs
N = 51
Known homologs
N = 1
Known homologs
N = 1
> 23.5
≤ 23.5
N = 6
N = 96
N = 0
N = 58
N = 2
N = 12
N = 3
N = 7
N = 8
N = 177
N = 2
N = 49
N = 0
N = 1
N = 0
N = 1

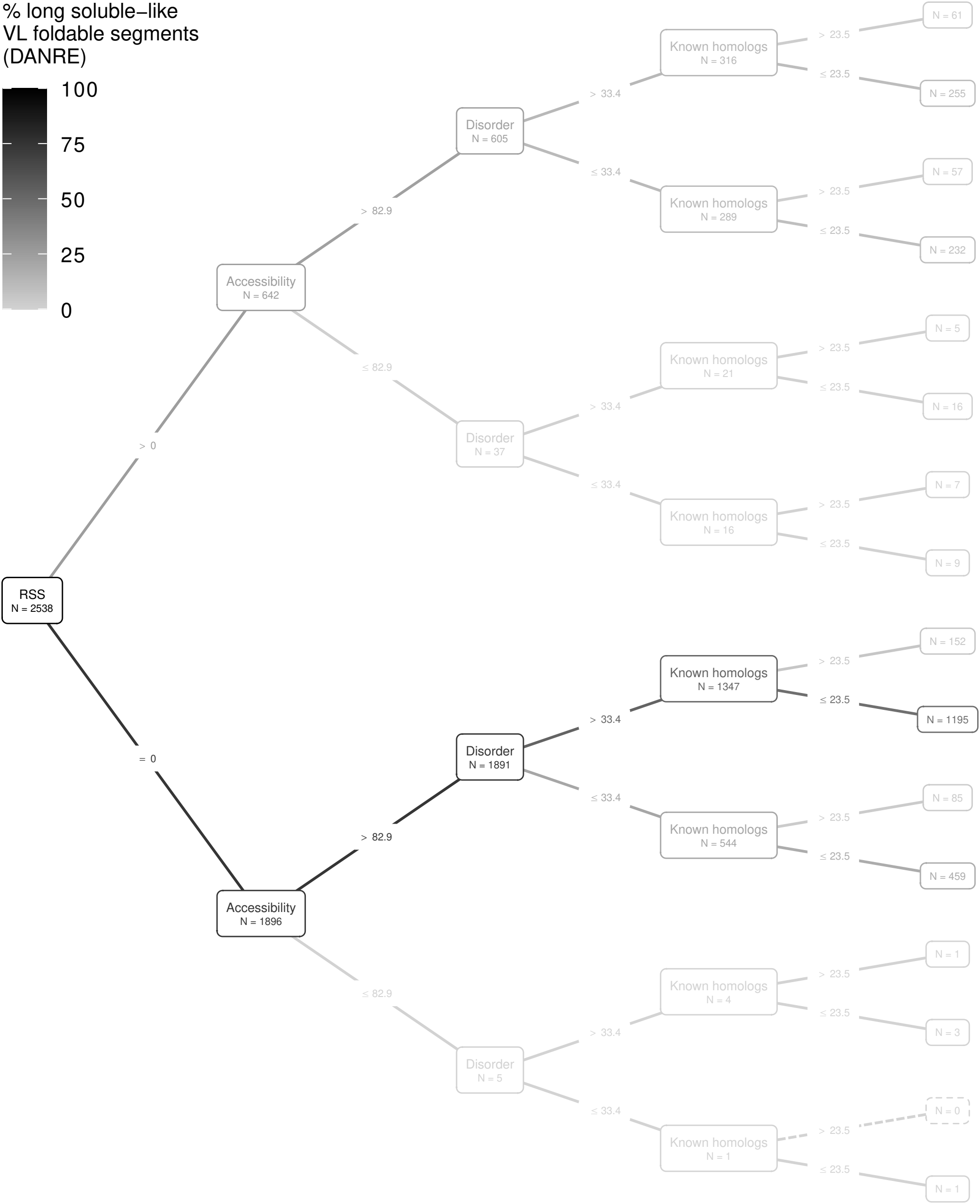
% long soluble−like
VL foldable segments
(DANRE)
100
75
50
25
0
RSS
N = 2538
> 0
= 0
Accessibility
N = 642
Accessibility
N = 1896
> 82.9
≤ 82.9
> 82.9
≤ 82.9
Disorder
N = 605
Disorder
N = 37
Disorder
N = 1891
Disorder
N = 5
> 33.4
≤ 33.4
> 33.4
≤ 33.4
> 33.4
≤ 33.4
> 33.4
≤ 33.4
Known homologs
N = 316
Known homologs
N = 289
Known homologs
N = 21
Known homologs
N = 16
Known homologs
N = 1347
Known homologs
N = 544
Known homologs
N = 4
Known homologs
N = 1
> 23.5
≤ 23.5
N = 61
N = 255
N = 57
N = 232
N = 5
N = 16
N = 7
N = 9
N = 152
N = 1195
N = 85
N = 459
N = 1
N = 3
N = 0
N = 1

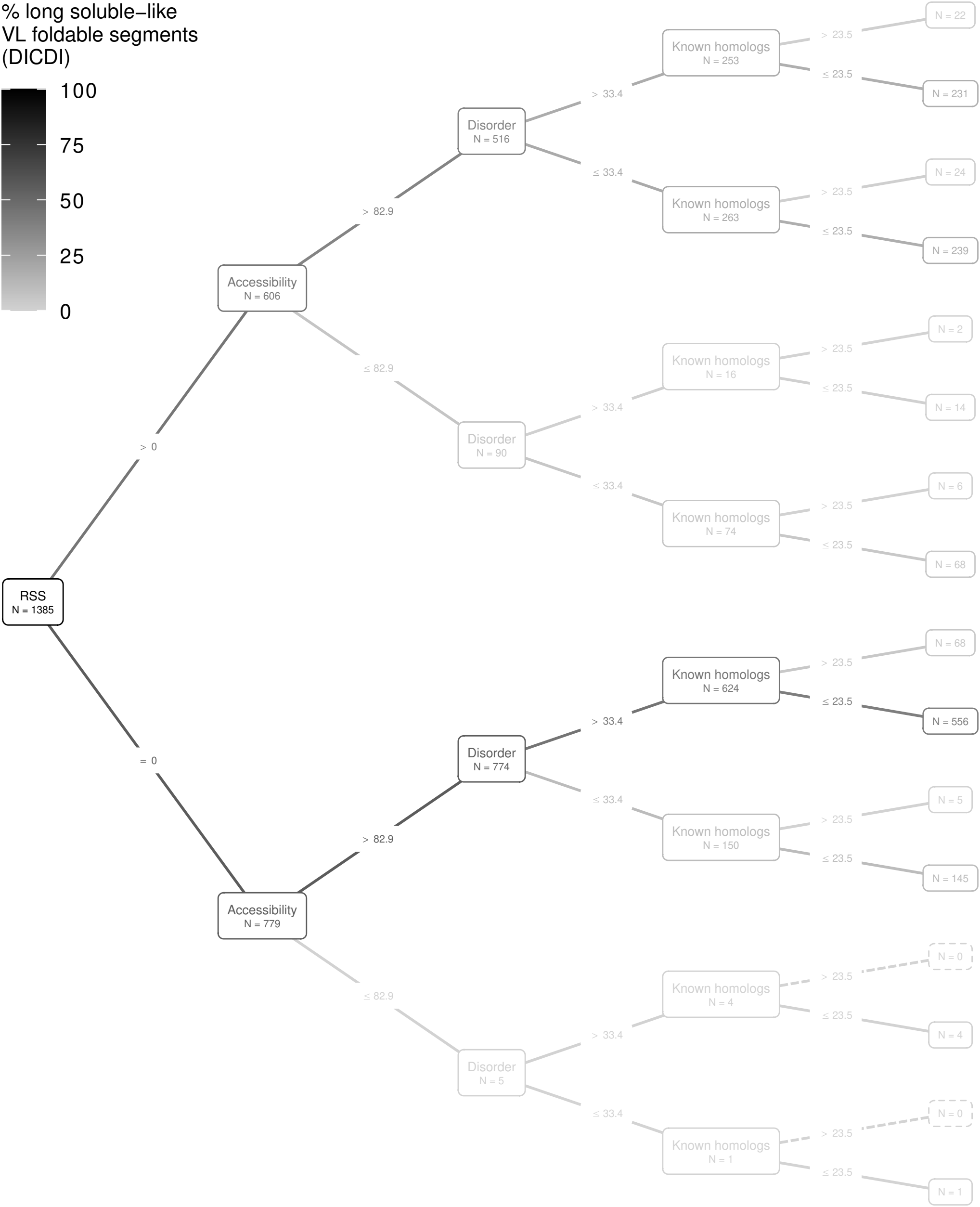

% long soluble−like
VL foldable segments
(DICDI)
100
75
50
25
0
RSS
N = 1385
> 0
= 0
Accessibility
N = 606
Accessibility
N = 779
> 82.9
≤ 82.9
Disorder
N = 516
Disorder
N = 90
Disorder
N = 774
Disorder
N = 5
> 33.4
≤ 33.4
Known homologs
N = 253
Known homologs
N = 263
Known homologs
N = 16
Known homologs
N = 74
Known homologs
N = 624
Known homologs
N = 150
Known homologs
N = 4
Known homologs
N = 1
> 23.5
≤ 23.5
N = 22
N = 231
N = 24
N = 239
N = 2
N = 14
N = 6
N = 68
N = 68
N = 556
N = 5
N = 145
N = 0
N = 4
N = 0
N = 1

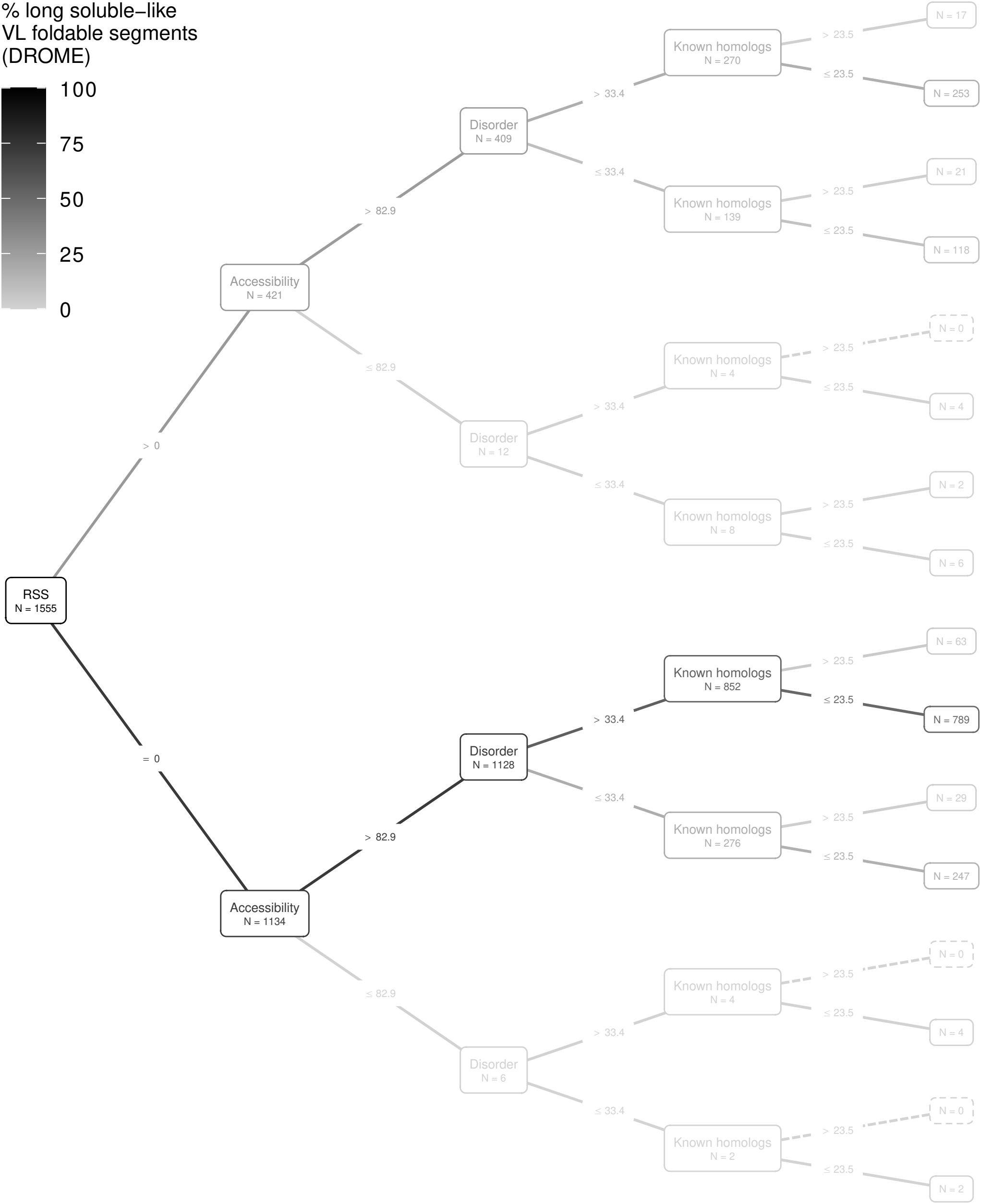
% long soluble−like
VL foldable segments
(DROME)
100
75
50
25
0
RSS
N = 1555
> 0
= 0
Accessibility
N = 421
Accessibility
N = 1134
> 82.9
≤ 82.9
Disorder
N = 409
Disorder
N = 12
Disorder
N = 1128
Disorder
N = 6
> 33.4
≤ 33.4
Known homologs
N = 270
Known homologs
N = 139
Known homologs
N = 4
Known homologs
N = 8
Known homologs
N = 852
Known homologs
N = 276
Known homologs
N = 4
Known homologs
N = 2
> 23.5
≤ 23.5
N = 17
N = 253
N = 21
N = 118
N = 0
N = 4
N = 2
N = 6
N = 63
N = 789
N = 29
N = 247
N = 0
N = 4
N = 0
N = 2

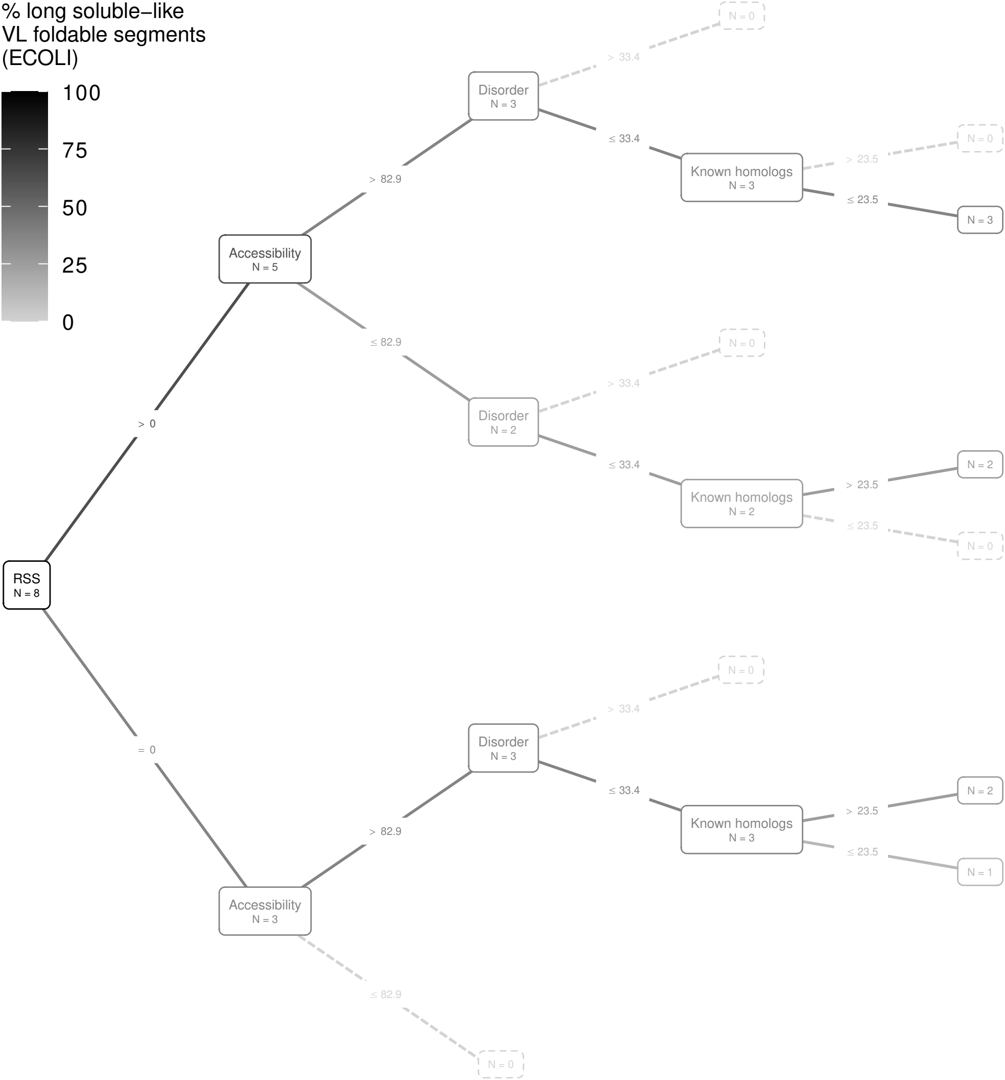

% long soluble−like
VL foldable segments
(ECOLI)
100
75
50
25
0
RSS
N = 8
> 0
= 0
Accessibility
N = 5
Accessibility
N = 3
> 82.9
≤ 82.9
Disorder
N = 3
Disorder
N = 2
Disorder
N = 3
> 33.4
≤ 33.4
Known homologs
N = 3
Known homologs
N = 2
> 23.5
≤ 23.5
N = 0
N = 3
N = 2
N = 1

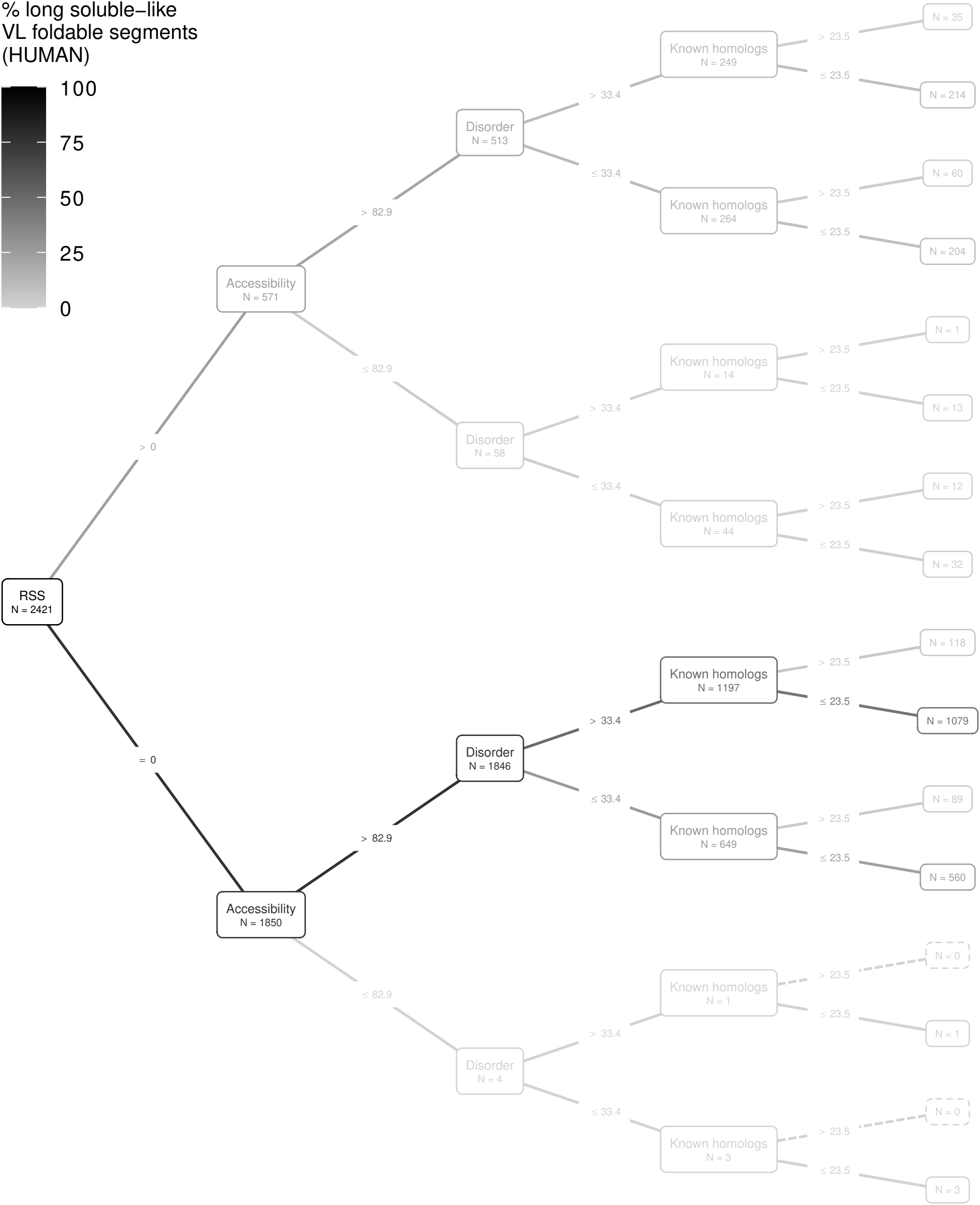

% long soluble−like VL foldable segments (HUMAN)
100
75
50
25
0
RSS N = 2421
> 0
= 0
Accessibility N = 571
Accessibility N = 1850
> 82.9
≤ 82.9
Disorder N = 513
Disorder N = 58
Disorder N = 1846
Disorder N = 4
> 33.4
≤ 33.4
Known homologs N = 249
Known homologs N = 264
Known homologs N = 14
Known homologs N = 44
Known homologs N = 1197
Known homologs N = 649
Known homologs N = 1
Known homologs N = 3
> 23.5
≤ 23.5
N = 35
N = 214
N = 60
N = 204
N = 1
N = 13
N = 12
N = 32
N = 118
N = 1079
N = 89
N = 560
N = 0
N = 1
N = 0
N = 3

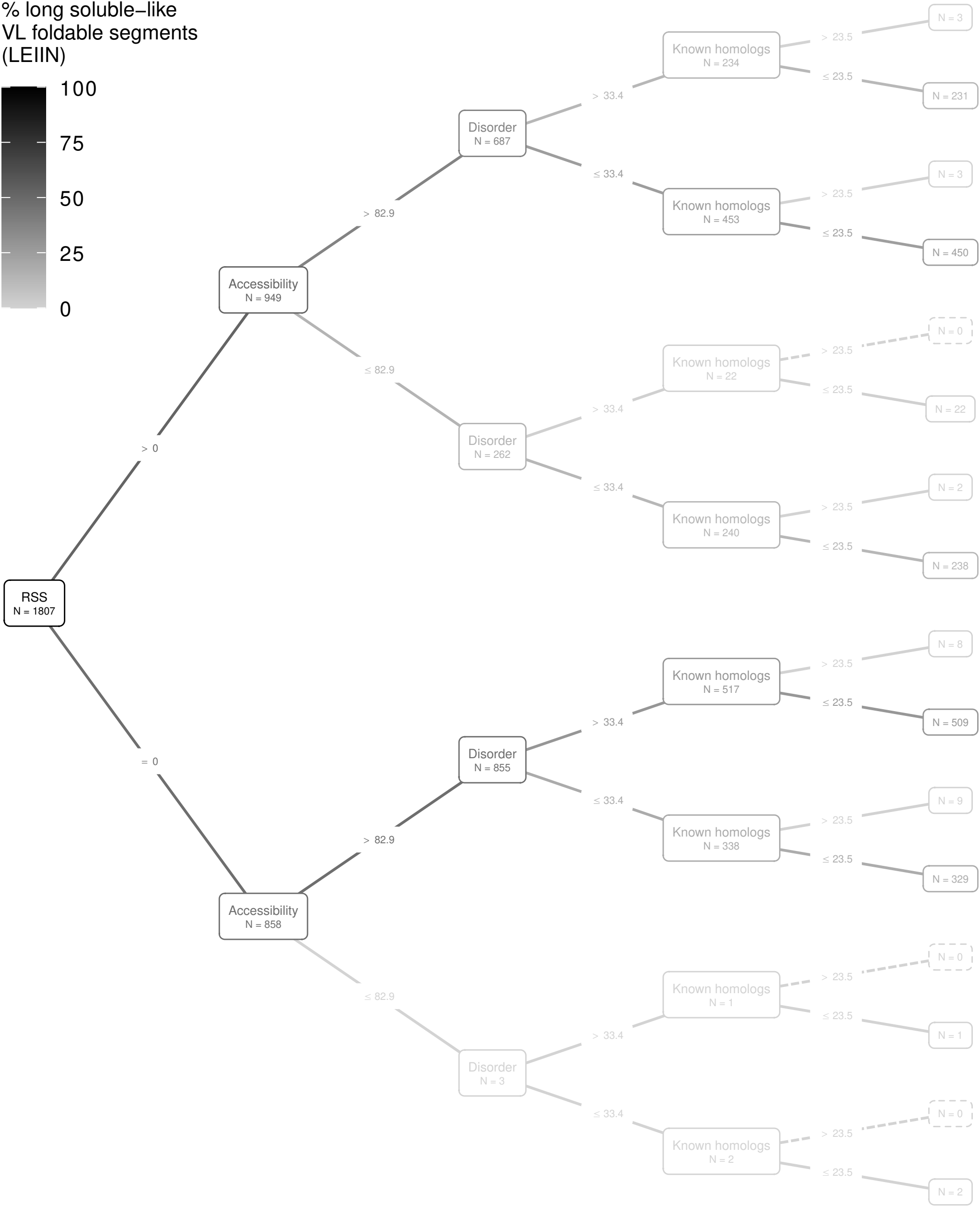
% long soluble−like VL foldable segments (LEIIN)
100
75
50
25
0
RSS
N = 1807
> 0
= 0
Accessibility
N = 949
Accessibility
N = 858
> 82.9
≤ 82.9
Disorder
N = 687
Disorder
N = 262
Disorder
N = 855
Disorder
N = 3
> 33.4
≤ 33.4
Known homologs
N = 234
Known homologs
N = 453
Known homologs
N = 22
Known homologs
N = 240
Known homologs
N = 517
Known homologs
N = 338
Known homologs
N = 1
Known homologs
N = 2
> 23.5
≤ 23.5
N = 3
N = 231
N = 3
N = 450
N = 0
N = 22
N = 2
N = 238
N = 8
N = 509
N = 9
N = 329
N = 0
N = 1
N = 0
N = 2

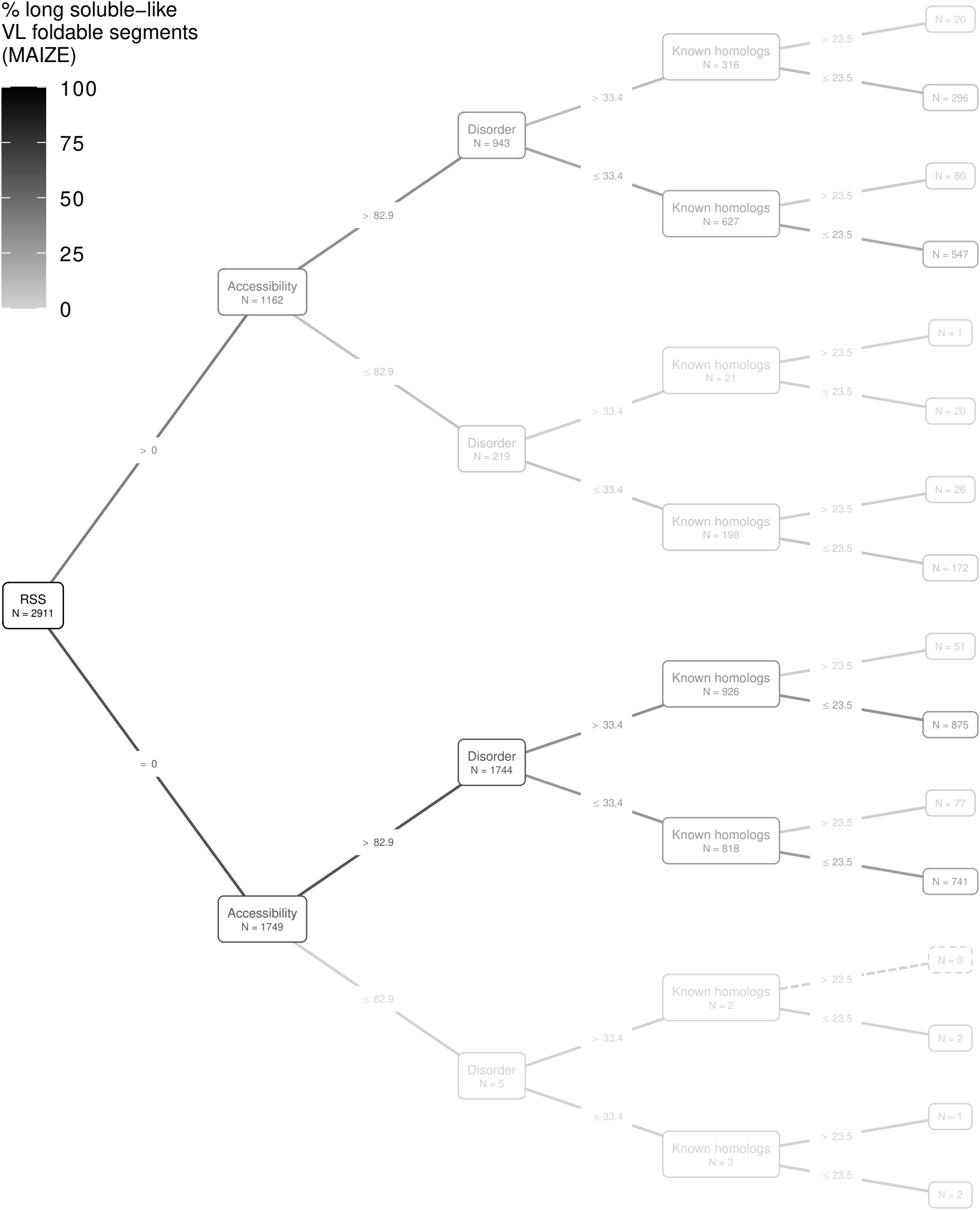

% long soluble−like
VL foldable segments
(MAIZE)
100
75
50
25
0
RSS
N = 2911
> 0
= 0
Accessibility
N = 1162
> 82.9
≤ 82.9
Disorder
N = 943
> 33.4
≤ 33.4
Known homologs
N = 316
> 23.5
≤ 23.5
N = 20
N = 296
Known homologs
N = 627
> 23.5
≤ 23.5
N = 80
N = 547
Disorder
N = 219
> 33.4
≤ 33.4
Known homologs
N = 21
> 23.5
≤ 23.5
N = 1
N = 20
Known homologs
N = 198
> 23.5
≤ 23.5
N = 26
N = 172
Accessibility
N = 1749
> 82.9
≤ 82.9
Disorder
N = 1744
> 33.4
≤ 33.4
Known homologs
N = 926
> 23.5
≤ 23.5
N = 51
N = 875
Known homologs
N = 818
> 23.5
≤ 23.5
N = 77
N = 741
Disorder
N = 5
> 33.4
≤ 33.4
Known homologs
N = 2
> 23.5
≤ 23.5
N = 0
N = 2
Known homologs
N = 3
> 23.5
≤ 23.5
N = 1
N = 2

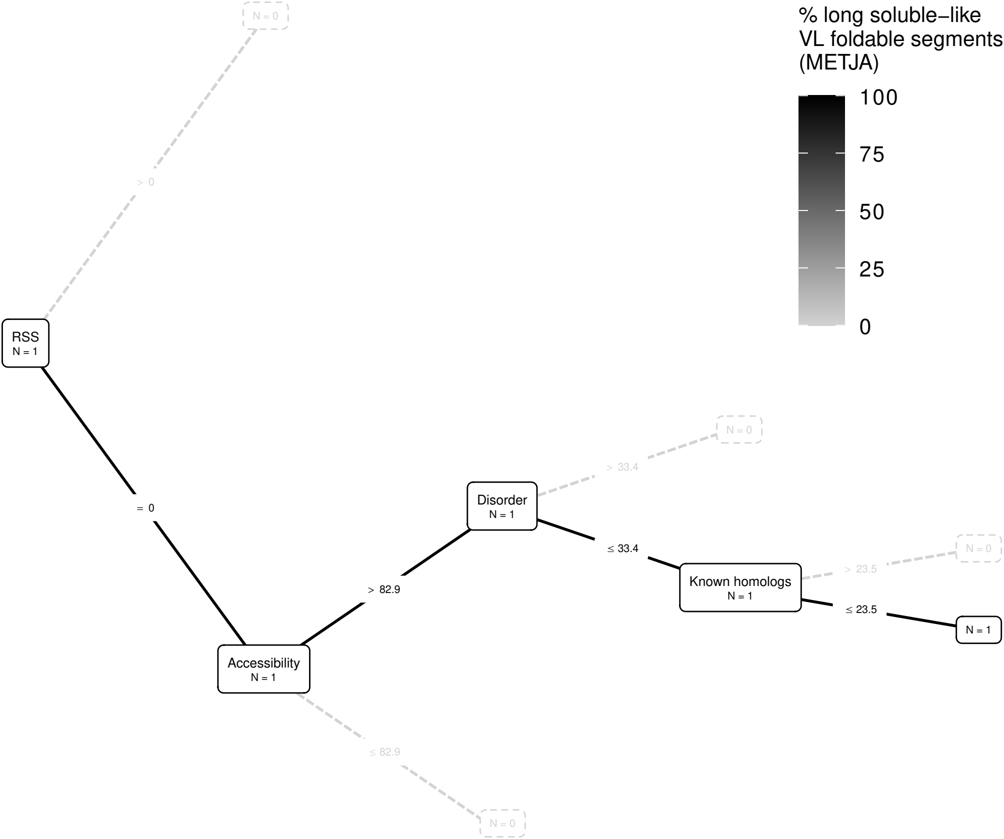
% long soluble−like
VL foldable segments
(METJA)
100
75
50
25
0
N = 0
> 0
RSS
N = 1
= 0
Accessibility
N = 1
> 82.9
≤ 82.9
N = 0
Disorder
N = 1
> 33.4
N = 0
≤ 33.4
Known homologs
N = 1
> 23.5
N = 0
≤ 23.5
N = 1

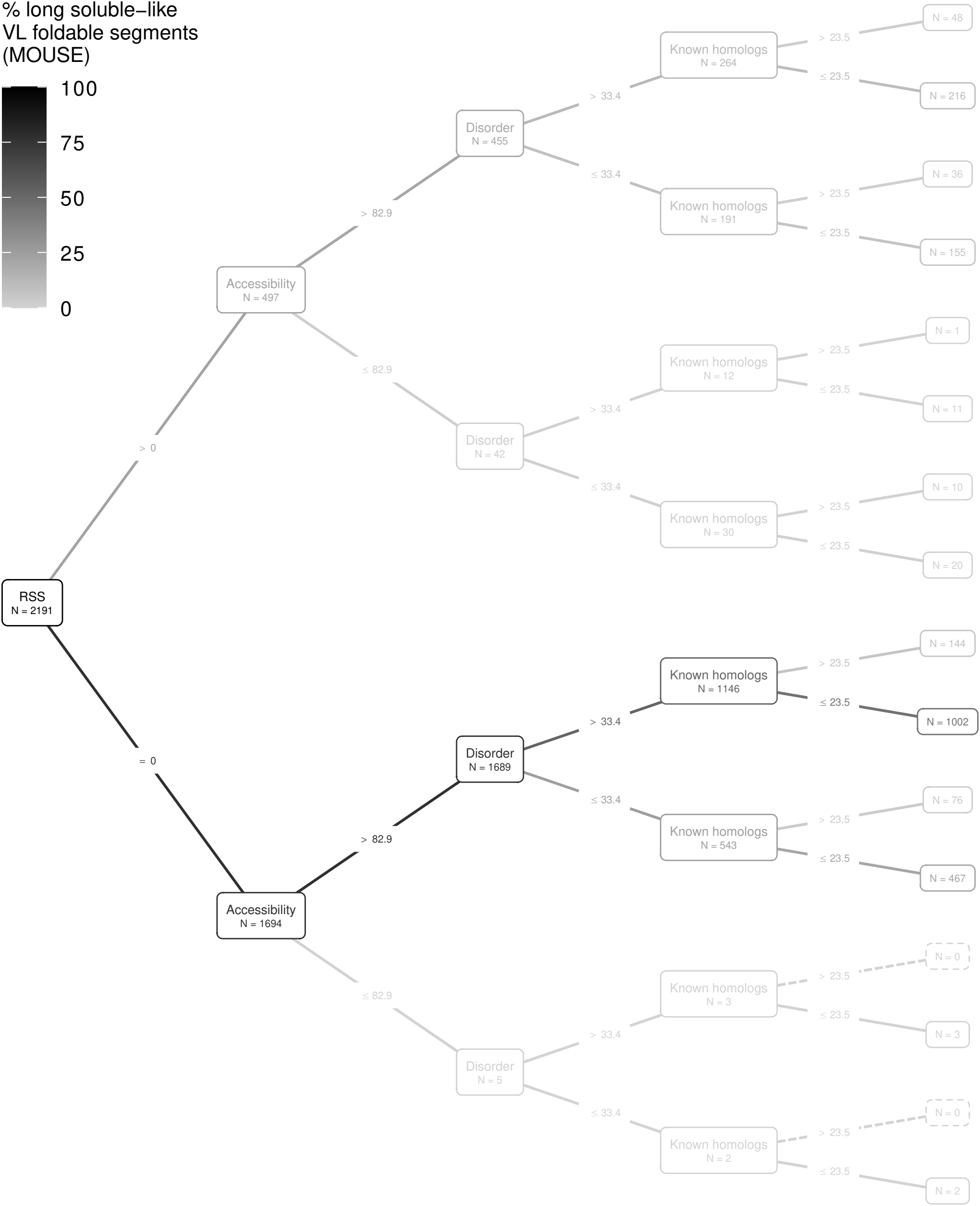
% long soluble−like VL foldable segments (MOUSE)
100
75
50
25
0
RSS
N = 2191
> 0
= 0
Accessibility
N = 497
Accessibility
N = 1694
> 82.9
≤ 82.9
Disorder
N = 455
Disorder
N = 42
Disorder
N = 1689
Disorder
N = 5
> 33.4
≤ 33.4
Known homologs
N = 264
N = 191
N = 12
N = 30
N = 1146
N = 543
N = 3
N = 2
> 23.5
≤ 23.5
N = 48
N = 216
N = 36
N = 155
N = 1
N = 11
N = 10
N = 20
N = 144
N = 1002
N = 76
N = 467
N = 0
N = 3
N = 0
N = 2

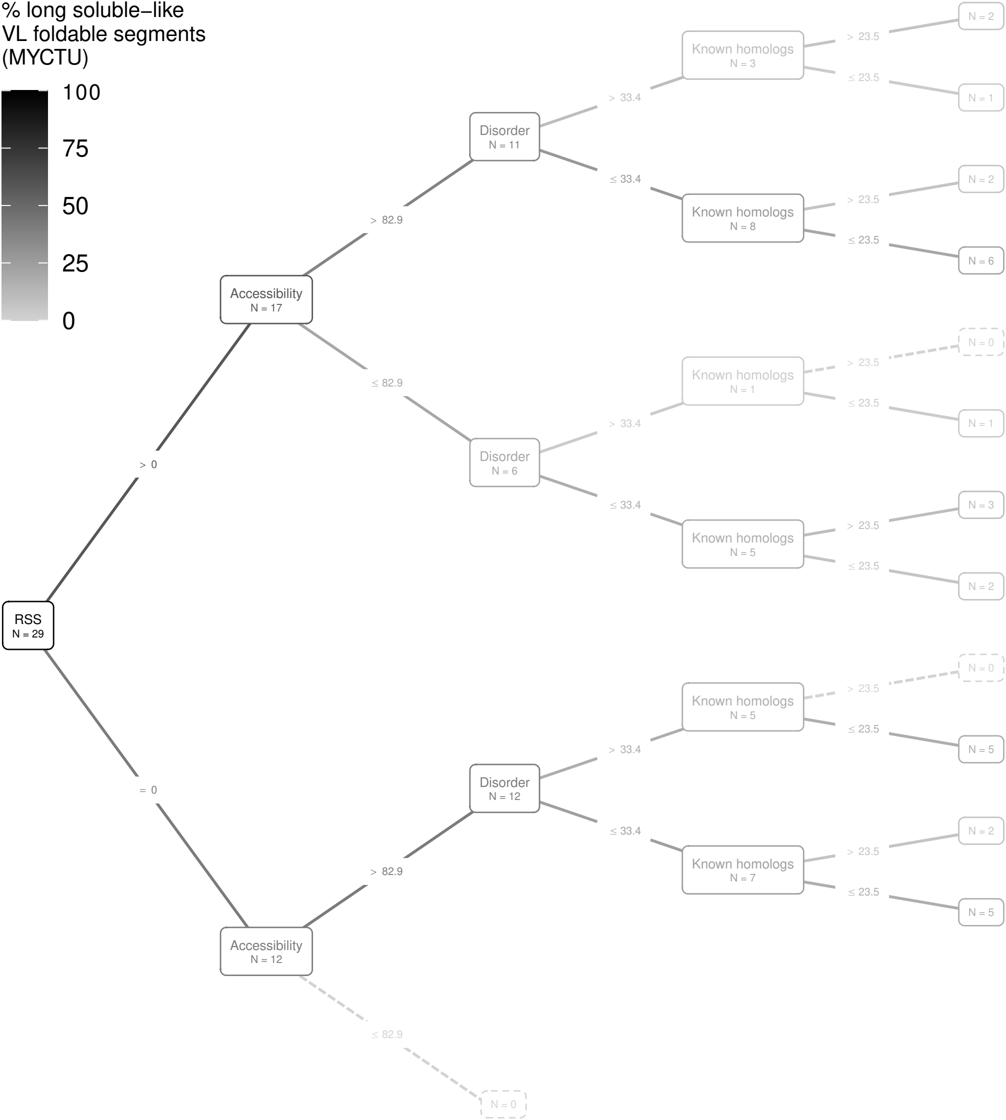

% long soluble–like
VL foldable segments
(MYCTU)
100
75
50
25
0
RSS
N = 29
> 0
= 0
Accessibility
N = 17
Accessibility
N = 12
> 82.9
≤ 82.9
Disorder
N = 11
Disorder
N = 6
Disorder
N = 12
N = 0
> 33.4
≤ 33.4
Known homologs
N = 3
Known homologs
N = 8
Known homologs
N = 1
Known homologs
N = 5
Known homologs
N = 5
Known homologs
N = 7
> 23.5
≤ 23.5
N = 2
N = 1
N = 2
N = 6
N = 0
N = 1
N = 3
N = 2
N = 0
N = 5
N = 2
N = 5

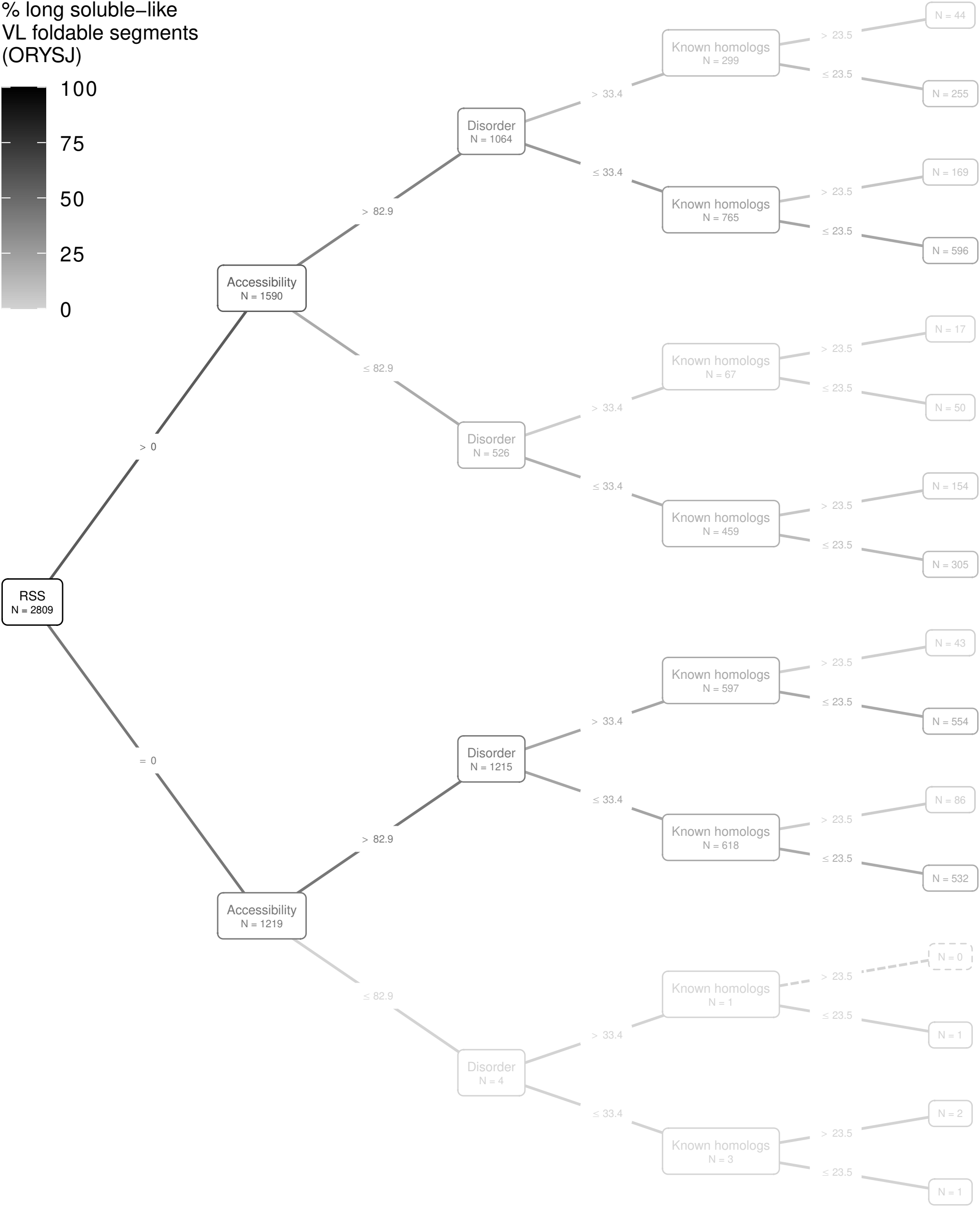

% long soluble−like
VL foldable segments
(ORYSJ)
100
75
50
25
0
RSS
N = 2809
> 0
= 0
Accessibility
N = 1590
Accessibility
N = 1219
> 82.9
≤ 82.9
Disorder
N = 1064
Disorder
N = 526
Disorder
N = 1215
Disorder
N = 4
> 33.4
≤ 33.4
Known homologs
N = 299
Known homologs
N = 765
Known homologs
N = 67
Known homologs
N = 459
Known homologs
N = 597
Known homologs
N = 618
Known homologs
N = 1
Known homologs
N = 3
> 23.5
≤ 23.5
N = 44
N = 255
N = 169
N = 596
N = 17
N = 50
N = 154
N = 305
N = 43
N = 554
N = 86
N = 532
N = 0
N = 1
N = 2
N = 1

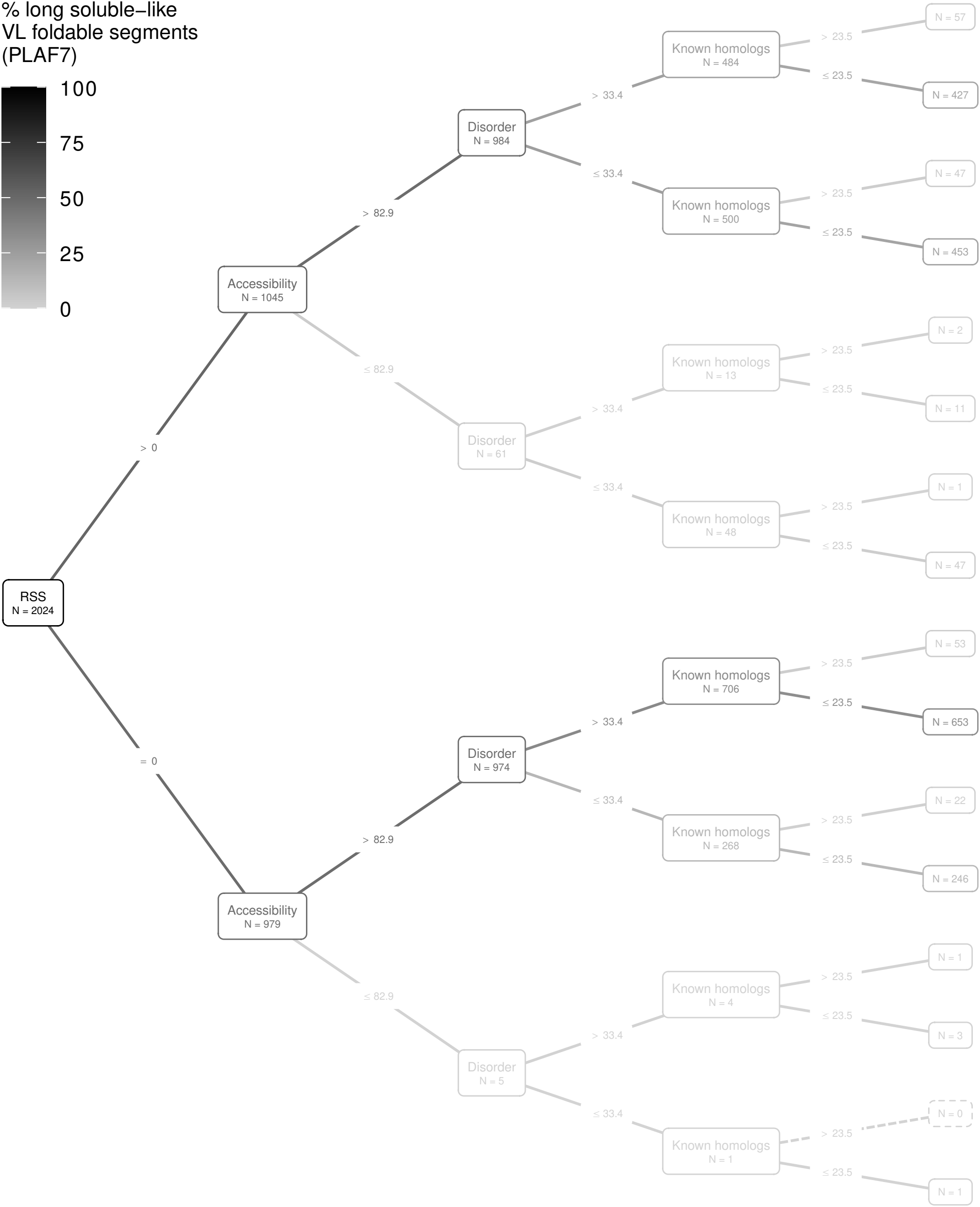
% long soluble−like
VL foldable segments
(PLAF7)
100
75
50
25
0
RSS
N = 2024
> 0
= 0
Accessibility
N = 1045
Accessibility
N = 979
> 82.9
≤ 82.9
> 82.9
≤ 82.9
Disorder
N = 984
Disorder
N = 61
Disorder
N = 974
Disorder
N = 5
> 33.4
≤ 33.4
> 33.4
≤ 33.4
> 33.4
≤ 33.4
> 33.4
≤ 33.4
Known homologs
N = 484
Known homologs
N = 500
Known homologs
N = 13
Known homologs
N = 48
Known homologs
N = 706
Known homologs
N = 268
Known homologs
N = 4
Known homologs
N = 1
> 23.5
≤ 23.5
N = 57
N = 427
N = 47
N = 453
N = 2
N = 11
N = 1
N = 47
N = 53
N = 653
N = 22
N = 246
N = 1
N = 3
N = 0
N = 1

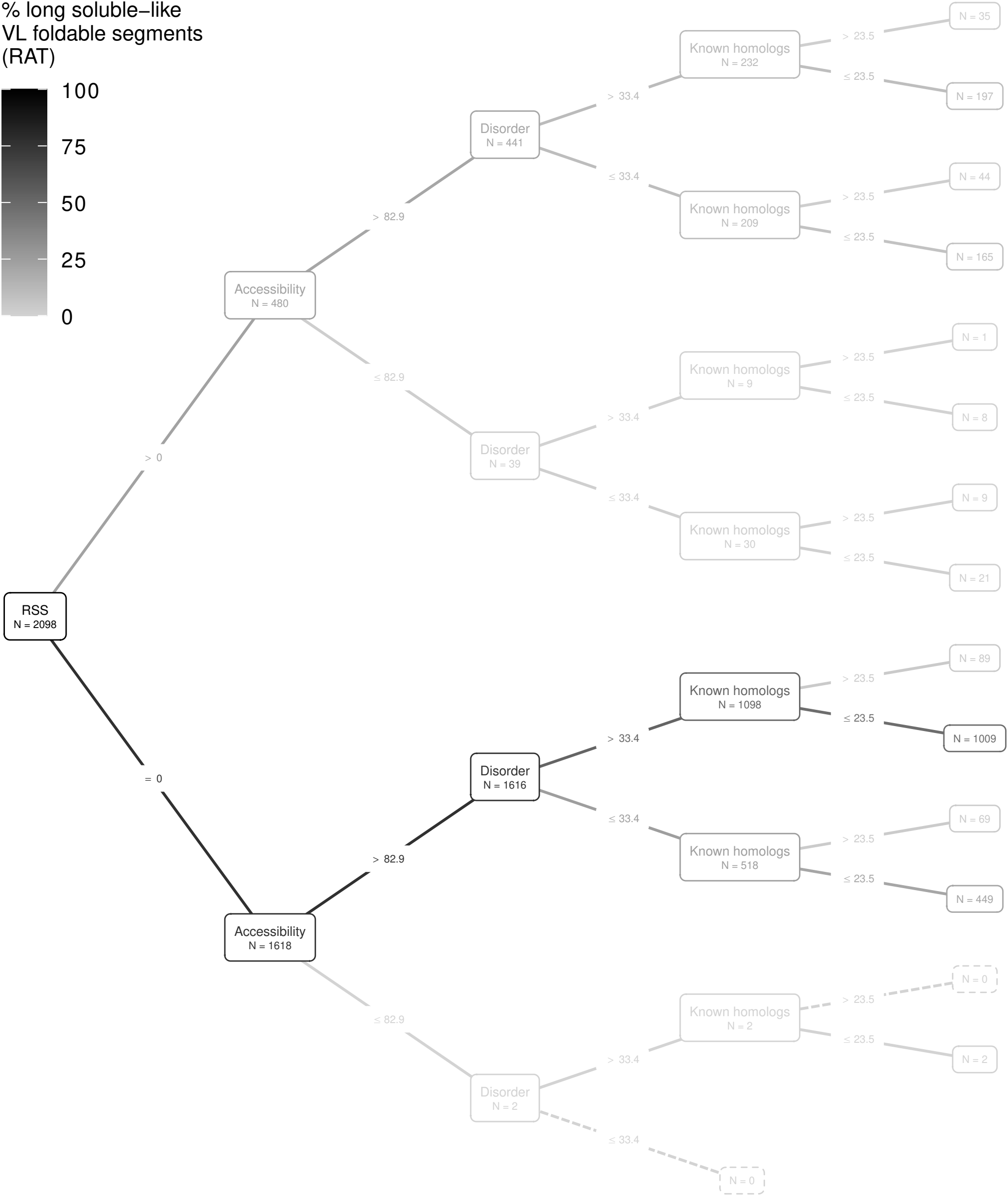

% long soluble−like
VL foldable segments
(RAT)
100
75
50
25
0
RSS
N = 2098
> 0
= 0
Accessibility
N = 480
Accessibility
N = 1618
> 82.9
≤ 82.9
Disorder
N = 441
Disorder
N = 39
Disorder
N = 1616
Disorder
N = 2
> 33.4
≤ 33.4
Known homologs
N = 232
Known homologs
N = 209
Known homologs
N = 9
Known homologs
N = 30
Known homologs
N = 1098
Known homologs
N = 518
Known homologs
N = 2
> 23.5
≤ 23.5
N = 35
N = 197
N = 44
N = 165
N = 1
N = 8
N = 9
N = 21
N = 89
N = 1009
N = 69
N = 449
N = 0
N = 2
N = 0

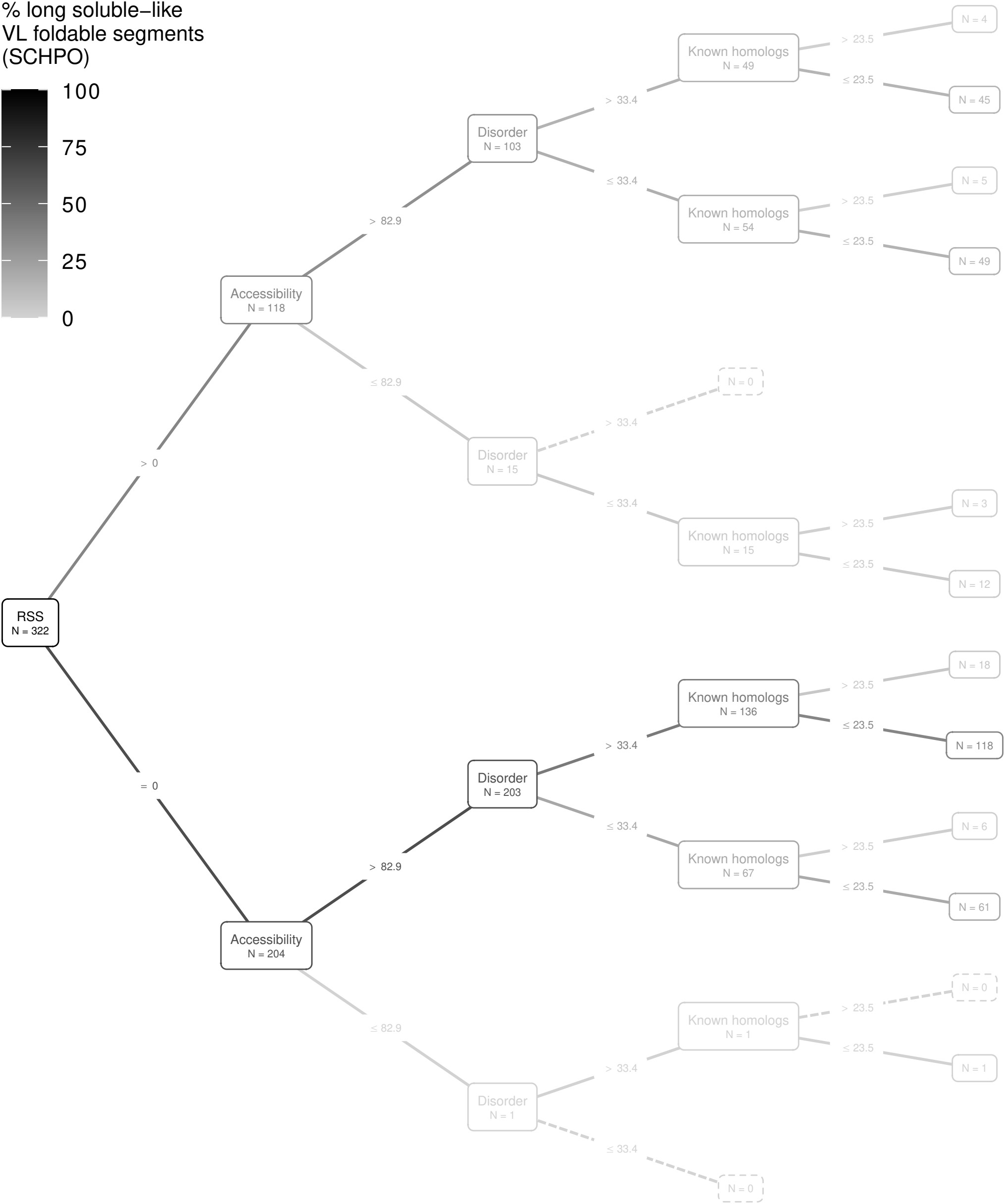

% long soluble−like
VL foldable segments
(SCHPO)
100
75
50
25
0
RSS
N = 322
> 0
= 0
Accessibility
N = 118
Accessibility
N = 204
> 82.9
≤ 82.9
Disorder
N = 103
Disorder
N = 15
Disorder
N = 203
Disorder
N = 1
> 33.4
≤ 33.4
Known homologs
N = 49
Known homologs
N = 54
N = 0
Known homologs
N = 15
Known homologs
N = 136
Known homologs
N = 67
Known homologs
N = 1
N = 0
> 23.5
≤ 23.5
N = 4
N = 45
N = 5
N = 49
N = 3
N = 12
N = 18
N = 118
N = 6
N = 61
N = 0
N = 1

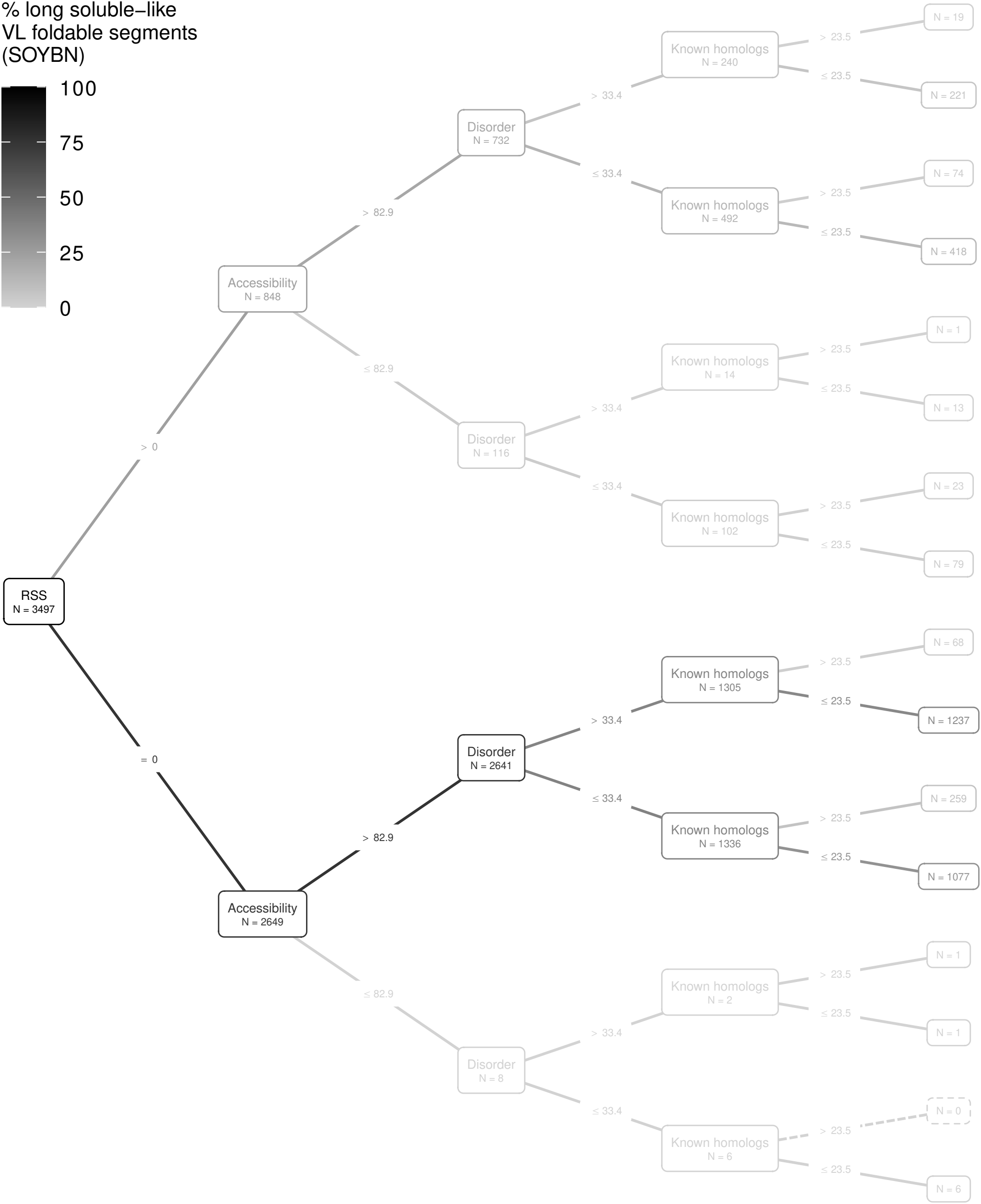
% long soluble−like VL foldable segments (SOYBN)
100
75
50
25
0
RSS
N = 3497
> 0
= 0
Accessibility
N = 848
Accessibility
N = 2649
> 82.9
≤ 82.9
Disorder
N = 732
Disorder
N = 116
Disorder
N = 2641
Disorder
N = 8
> 33.4
≤ 33.4
Known homologs
N = 240
Known homologs
N = 492
Known homologs
N = 14
Known homologs
N = 102
Known homologs
N = 1305
Known homologs
N = 1336
Known homologs
N = 2
Known homologs
N = 6
> 23.5
≤ 23.5
N = 19
N = 221
N = 74
N = 418
N = 1
N = 13
N = 23
N = 79
N = 68
N = 1237
N = 259
N = 1077
N = 1
N = 1
N = 0
N = 6

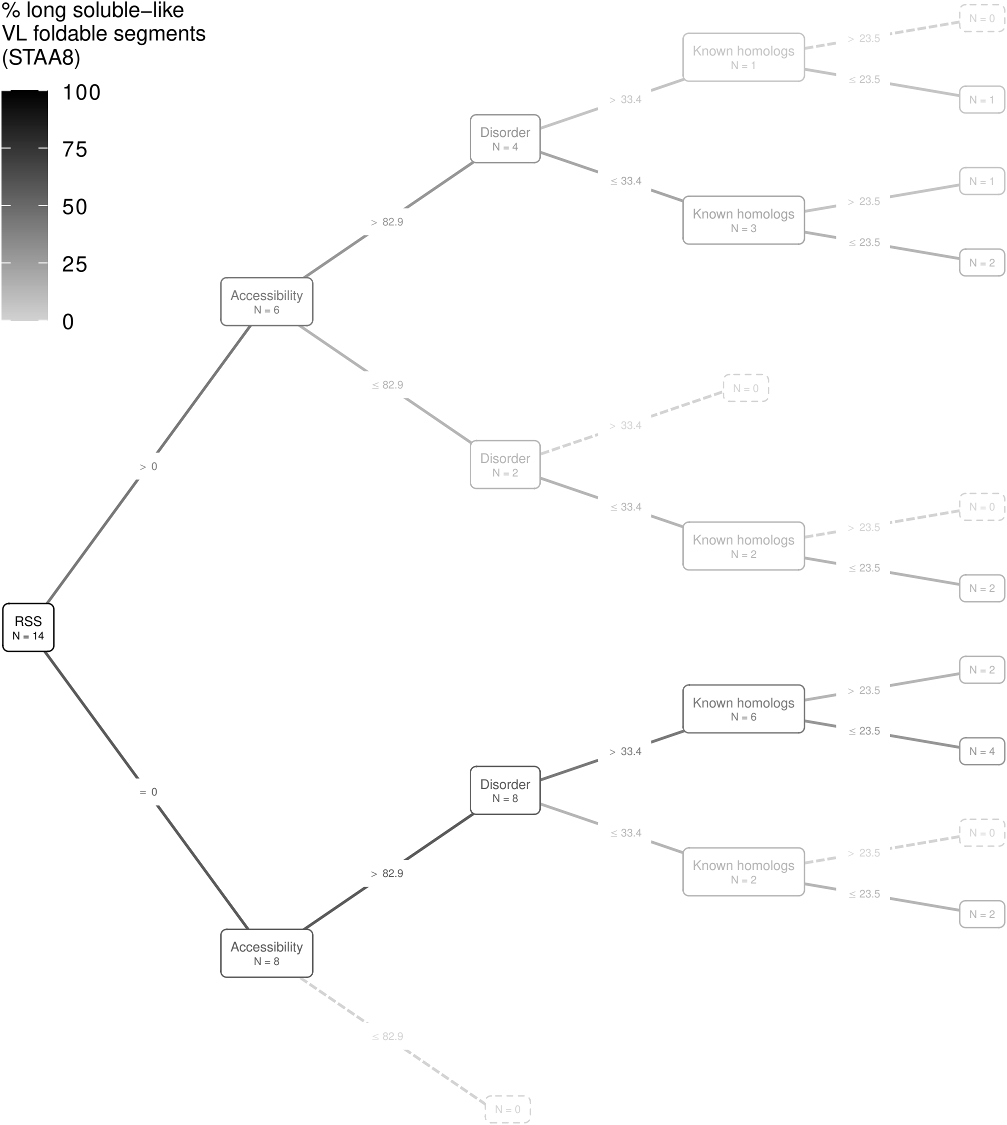

% long soluble−like
VL foldable segments
(STAA8)
100
75
50
25
0
RSS
N = 14
> 0
= 0
Accessibility
N = 6
Accessibility
N = 8
> 82.9
≤ 82.9
Disorder
N = 4
Disorder
N = 2
Disorder
N = 8
N = 0
> 33.4
≤ 33.4
Known homologs
N = 1
Known homologs
N = 3
Known homologs
N = 2
Known homologs
N = 6
Known homologs
N = 2
> 23.5
≤ 23.5
N = 0
N = 1
N = 1
N = 2
N = 0
N = 2
N = 2
N = 4
N = 0
N = 2

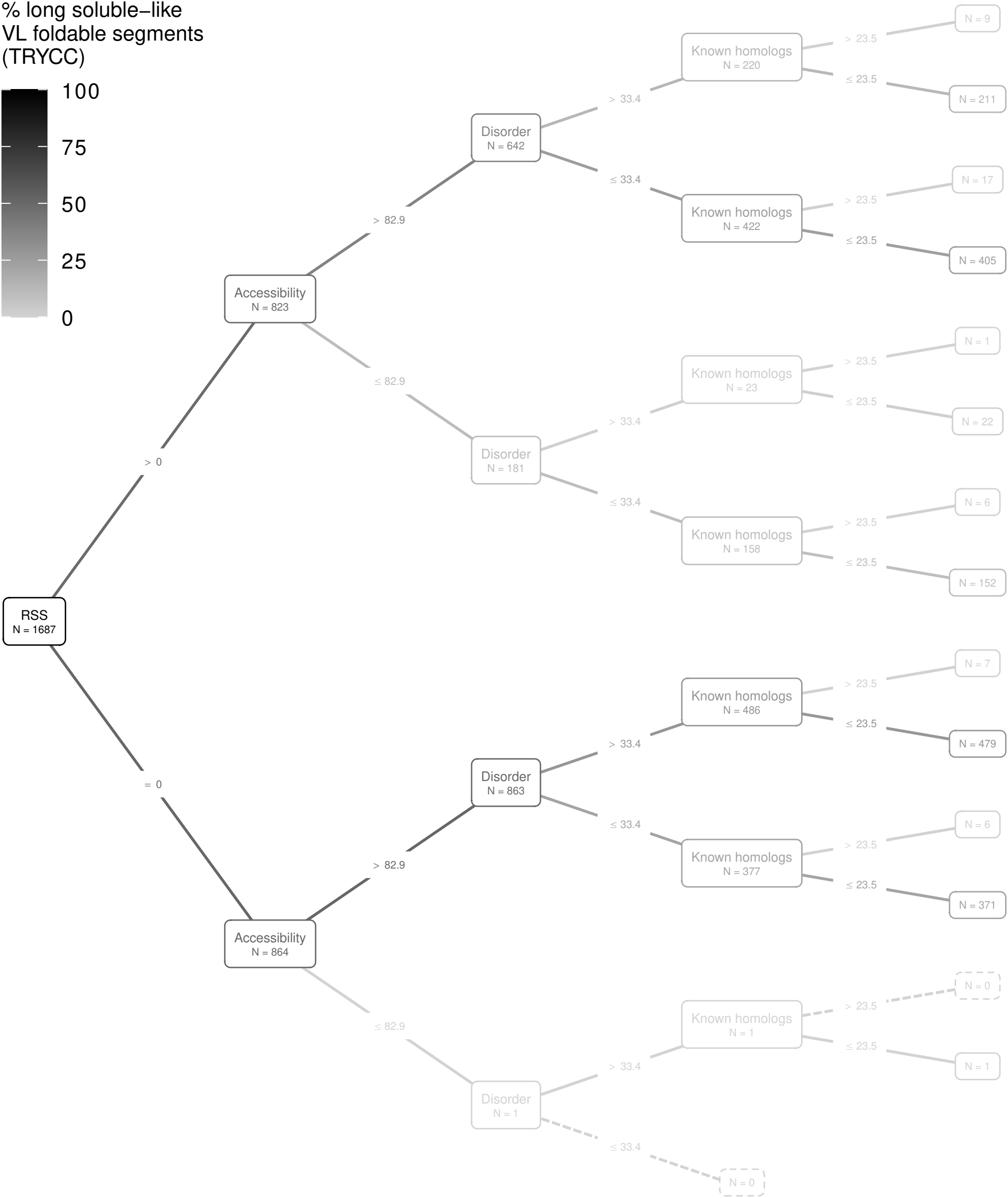
% long soluble−like
VL foldable segments
(TRYCC)
100
75
50
25
0
RSS
N = 1687
> 0
= 0
Accessibility
N = 823
Accessibility
N = 864
> 82.9
≤ 82.9
Disorder
N = 642
Disorder
N = 181
Disorder
N = 863
Disorder
N = 1
> 33.4
≤ 33.4
Known homologs
N = 220
Known homologs
N = 422
Known homologs
N = 23
Known homologs
N = 158
Known homologs
N = 486
Known homologs
N = 377
Known homologs
N = 1
N = 0
> 23.5
≤ 23.5
N = 9
N = 211
N = 17
N = 405
N = 1
N = 22
N = 6
N = 152
N = 7
N = 479
N = 6
N = 371
N = 0
N = 1

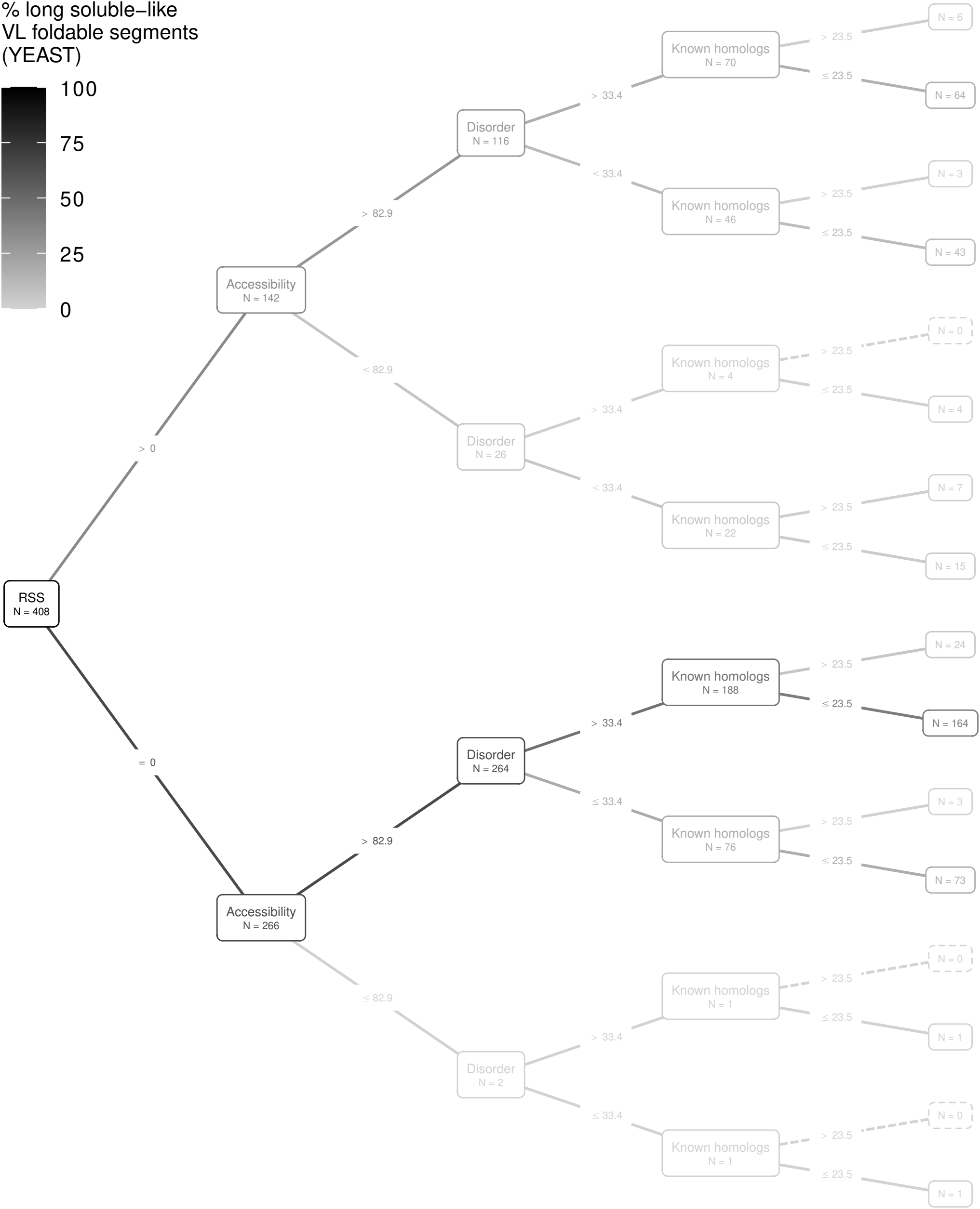
% long soluble−like
VL foldable segments
(YEAST)
100
75
50
25
0
RSS
N = 408
> 0
= 0
Accessibility
N = 142
Accessibility
N = 266
> 82.9
≤ 82.9
Disorder
N = 116
Disorder
N = 26
Disorder
N = 264
Disorder
N = 2
> 33.4
≤ 33.4
Known homologs
N = 70
Known homologs
N = 46
Known homologs
N = 4
Known homologs
N = 22
Known homologs
N = 188
Known homologs
N = 76
Known homologs
N = 1
Known homologs
N = 1
> 23.5
≤ 23.5
N = 6
N = 64
N = 3
N = 43
N = 0
N = 4
N = 7
N = 15
N = 24
N = 164
N = 3
N = 73
N = 0
N = 1
N = 0
N = 1
