# Supplementary material for: Digging into the 3D Structure Predictions of AlphaFold2 with Low Confidence: Disorder and Beyond
Source: Biomolecules. 2022 Oct 13;12(10):1467. doi: 10.3390/biom12101467 (PMC9599455; doi:10.3390/biom12101467)
Supplement: Supplementary file 1 [file biomolecules-12-01467-s001.zip › Table S1.pdf]

Table S1. Distribution of VH and VL residues within the long soluble-like foldable segments of the AFDB v1 dataset (21 proteomes). Minimum and maximum values are underlined (percentages, segment mean length). The proteomes are top-down sorted according to their increasing number of long soluble-like foldable segments (N). *3104 sequences were excluded from our analyses, as sequences longer than 2700 amino acids were not modeled in AFDB v1.

Species (id in AFDB v1)	Proteome id in UniProt	Sequence number*	Residue number (Nr)	Long soluble-like foldable segments							
				N	Residues: number, %Nr	VL residue number (VL_tot)	Full-VL segments: number, mean length	Full-VL segments: residue number, %VL_tot	VH residue number (VH_tot)	Full-VH segments: number, mean length	Full-VH segments: residue number, %VH_tot
Methanocaldococcus jannaschii (METJA)	UP000000805	1 773	497 291	1 493	347 897 (70.0%)	9 664	1 (35.0 aa)	35 (0.4%)	268 766	83 (120.9 aa)	10 037 (3.7%)
Staphylococcus aureus (STAA8)	UP000008816	2 888	787 862	2 746	536 461 (68.1%)	10 916	14 (49.0 aa)	686 (6.3%)	415 301	201 (111.9 aa)	22 487 (5.4%)
Escherichia coli (ECOLI)	UP000000625	4 363	1 349 433	5 262	996 863 (73.9%)	17 116	8 (65.3 aa)	522 (3.1%)	765 865	564 (109.3 aa)	61 666 (8.1%)
Mycobacterium tuberculosis (MYCTU)	UP000001584	3 988	1 313 964	5 939	925 656 (70.4%)	28 335	29 (53.3 aa)	1 547 (5.5%)	683 195	583 (83.0 aa)	48 404 (7.1%)
Schizosaccharomyces pombe (SCHPO)	UP000002485	5 128	2 345 773	7 869	1 664 086 (70.9%)	165 452	322 (57.6 aa)	18 560 (11.2%)	866 109	182 (90.2 aa)	16 419 (1.9%)
Saccharomyces cerevisiae (YEAST)	UP000002311	6 040	2 902 659	9 799	1 928 423 (66.4%)	189 995	408 (53.7 aa)	21 905 (11.5%)	989 289	242 (97.8 aa)	23 661 (2.4%)
Candida albicans (CANAL)	UP000000559	5 974	2 918 918	9 850	1 874 606 (64.2%)	170 807	422 (54.7 aa)	23 073 (13.5%)	1 007 381	268 (95.8 aa)	25 676 (2.6%)
Plasmodium falciparum (PLAF7)	UP000001450	5 187	3 343 123	10 107	1 938 785 (58.0%)	782 869	2 024 (92.8 aa)	187 803 (24.0%)	461 940	32 (116.5 aa)	3 727 (0.8%)
Leishmania infantum (LEIIN)	UP000008153	7 924	4 678 533	19 010	2 596 542 (55.5%)	448 233	1 807 (55.6 aa)	100 425 (22.4%)	998 736	220 (91.6 aa)	20 151 (2.0%)
Dictyostelium discoideum (DICDI)	UP000002195	12 622	6 507 765	20 776	3 971 159 (61.0%)	636 863	1 385 (60.9 aa)	84 384 (13.3%)	1 653 192	204 (87.5 aa)	17 847 (1.1%)
Drosophila melanogaster (DROME)	UP000000803	13 458	6 656 778	23 182	4 008 537 (60.2%)	512 807	1 555 (53.9 aa)	83 778 (16.3%)	2 011 396	363 (87.1 aa)	31 608 (1.6%)
Caenorhabditis elegans (CAEEL)	UP000001940	19 694	7 812 030	26 172	4 939 911 (63.2%)	566 456	939 (55.2 aa)	51 787 (9.1%)	2 370 585	381 (87.4 aa)	33 296 (1.4%)
Trypanosoma cruzi (TRYCC)	UP000002296	19 036	8 959 621	33 746	5 748 836 (64.2%)	899 984	1 687 (60.1 aa)	101 448 (11.3%)	2 248 528	383 (86.7 aa)	33 204 (1.5%)
Rattus norvegicus (RAT)	UP000002494	21 270	10 424 727	36 248	6 363 218 (61.0%)	764 075	2 098 (56.0 aa)	117 567 (15.4%)	3 241 197	795 (93.7 aa)	74 458 (2.3%)
Mus musculus (MOUSE)	UP000000589	21 613	10 879 808	37 408	6 645 979 (61.1%)	793 555	2 191 (56.2 aa)	123 022 (15.5%)	3 391 084	785 (97.6 aa)	76 606 (2.3%)
Homo sapiens (HUMAN)	UP000005640	20 294	10 532 415	37 574	6 504 290 (61.8%)	836 065	2 421 (59.5 aa)	143 962 (17.2%)	3 264 186	784 (93.5 aa)	73 324 (2.2%)
Arabidopsis thaliana (ARATH)	UP000006548	27 434	11 037 703	40 673	7 715 899 (69.9%)	916 293	1 578 (59.1 aa)	93 328 (10.2%)	4 141 019	822 (92.5 aa)	76 000 (1.8%)
Danio rerio (DANRE)	UP000000437	24 661	12 654 340	44 349	8 187 933 (64.7%)	939 278	2 538 (55.8 aa)	141 544 (15.1%)	4 179 325	741 (88.9 aa)	65 910 (1.6%)
Oryza sativa (ORYSJ)	UP000059680	43 649	13 374 028	52 282	8 135 335 (60.8%)	1 109 607	2 809 (61.4 aa)	172 581 (15.6%)	4 125 257	1 085 (87.2 aa)	94 606 (2.3%)
Zea mays (MAIZE)	UP000007305	39 299	15 340 862	59 864	9 901 232 (64.5%)	1 471 243	2 911 (60.1 aa)	174 897 (11.9%)	4 601 754	1 184 (83.9 aa)	99 336 (2.2%)
Glycine max (SOYBN)	UP000008827	55 799	21 578 168	79 666	14 947 524 (69.3%)	2 019 537	3 497 (61.7 aa)	215 600 (10.7%)	7 567 348	1 493 (91.7 aa)	136 926 (1.8%)
**Total**		**362 094**	**155 895 801**	**564 015**	**99 879 172 (64.1%)**	**13 289 150**	**30 644 (60.7 aa)**	**1 858 454 (14.0%)**	**49 251 453**	**11 395 (91.7 aa)**	**1 045 349 (2.1%)**
